# The biosynthetic-secretory pathway, supplemented by recycling routes, determines epithelial membrane polarity

**DOI:** 10.1126/sciadv.ade4620

**Published:** 2023-06-28

**Authors:** Nan Zhang, Hongjie Zhang, Liakot A. Khan, Gholamali Jafari, Yong Eun, Edward Membreno, Verena Gobel

**Affiliations:** ^1^ Mucosal Immunology and Biology Research Center, Developmental Biology and Genetics Core, Massachusetts General Hospital for Children, Harvard Medical School, Boston, MA, USA.; ^2^ Key Laboratory of Zoonosis Research by the Ministry of Education, Institute of Zoonosis, College of Veterinary Medicine, Jilin University, Changchun 130062, China.; ^3^ Faculty of Health Sciences, University of Macau, Taipa, Macau, China.; ^4^ Department of Medicine, NYC Health & Hospitals/Harlem, Columbia University, New York, NY, USA.

## Abstract

In prevailing epithelial polarity models, membrane-based polarity cues (e.g., the partitioning-defective PARs) position apicobasal cellular membrane domains. Intracellular vesicular trafficking expands these domains by sorting polarized cargo toward them. How the polarity cues themselves are polarized in epithelia and how sorting confers long-range apicobasal directionality to vesicles is still unclear. Here, a systems-based approach using two-tiered *C. elegans* genomics-genetics screens identifies trafficking molecules that are not implicated in apical sorting yet polarize apical membrane and PAR complex components. Live tracking of polarized membrane biogenesis indicates that the biosynthetic-secretory pathway, linked to recycling routes, is asymmetrically oriented toward the apical domain during this domain’s biosynthesis, and that this directionality is regulated upstream of PARs and independent of polarized target membrane domains. This alternative mode of membrane polarization could offer solutions to open questions in current models of epithelial polarity and polarized trafficking.

## Teaser

Biosynthetic trafficking polarizes epithelial membranes by asymmetrically inserting the apical domain into the growing membrane.

## INTRODUCTION

Current epithelial polarity models order the highly conserved extracellular-, intracellular-, and membrane-based polarity cues sequentially during the process of cellular polarization: (i) Extracellular matrix signals orient cells within the epithelium; (ii) plasma membrane– and junction-based apical and basolateral core polarity determinants (the partitioning-defective PAR, Crumbs, and Scribble polarity complexes) specify apicobasal domain positions at the plasma membrane; (iii) intracellular vesicular trafficking expands and maintains these domains by supplying polarized membrane components (*1*–*4*). This hierarchy of events is thought to control both the initial polarization of the cell membrane during polarized tissue morphogenesis and the initial asymmetric delivery of membrane components to the plasma membrane during polarized membrane biogenesis itself. Such a polarity model is consistent with the current model of polarized trafficking where vesicles sort apicobasal cargo to cognate recognition sites at target plasma membrane domains, a process that requires these domains’ prior polarization (*1*, *5*–*7*). Unresolved difficulties of these models include the questions of how the membrane-based polarity cues themselves are polarized to domains whose positions they ought to specify, and of how the sorting of polarized cargo confers long-range apicobasal directionality to vesicles.

We previously demonstrated that glycosphingolipids (GSLs), saturated obligate membrane lipids, function in apicobasal membrane polarity in the *Caenorhabditis elegans* (*C. elegans*) intestine. Reducing and restoring GSL biosynthesis reversibly shifts apical membrane components, junctions, PARs, and the lumen between apical and basolateral sides of growing cells that no longer divide and move but still expand their plasma membranes (in vivo apicobasal polarity conversion/reversion) (*8*). GSLs, components of endomembrane and plasma membrane microdomains (rafts), have conserved roles in apical sorting on Golgi and post-Golgi vesicle membranes (*5*), suggesting that polarity conversion arose from a trafficking defect involving apicobasal sorting. However, the ability of trafficking to generate junction-bounded apical domains (lumens) on basolateral membranes and thus override the polarity established by the membrane-based core polarity cues also raised the possibility that trafficking might directly polarize the membrane while synthesizing it, e.g., on biosynthetic routes whose directionality was regulated independently of previously polarized target membrane domains. Early epithelial polarity models had envisioned a similar scenario, where the predetermined (apical or basolateral) directionality of bulk membrane delivery directly polarizes the growing membrane (*9*, *10*).

In support of such an upstream function of trafficking in membrane polarity, we and others subsequently identified components of the conserved post-Golgi vesicle coat clathrin and its AP-1 adaptor by a similar loss-of-function polarity conversion phenotype in the *C. elegans* intestine (*11*, *12*). However, GSLs have a wide range of trafficking-dependent and trafficking-independent functions, and clathrin and AP-1, the former (clathrin) best known for its role in endocytosis, also operate on multiple trafficking routes (*13*, *14*). These molecules might therefore alter membrane polarity by other mechanisms: independent of trafficking, by canonical sorting, on other than biosynthetic trafficking routes. They could, for instance, sort on endocytic-recycling routes that reposition previously delivered, rather than position newly synthesized, membrane components and thus maintain or rearrange the polarity of the membrane rather than polarizing it during its biosynthesis. In favor of this latter possibility, various endocytic- and transcytotic-recycling molecules have been identified in vitro and in vivo that recruit apical PARs to maintain apical/anterior polarity in different cell types and species (*15*–*19*) and position the apical domain in tubular epithelia (*20*–*27*). In contrast, no biosynthetic-secretory trafficking molecules were yet identified in multiple screens on the establishment and maintenance of apicobasal polarity, despite these molecules’ expected contribution to polarity via apicobasal sorting (*19*).

To comprehensively investigate the role of trafficking in membrane polarity, we here used a systems-based approach, taking advantage of the high molecular and functional conservation of vesicular trafficking components. First, we collected all trafficking molecules identified in our genome-scale multicellular (intestinal) and unicellular (excretory canal) *C. elegans* tubulogenesis screens (*8*, *28*) that affected apical membrane biogenesis. Next, we examined these molecules’ ability to modify the GSL-dependent intestinal apicobasal polarity conversion. This approach distinguished unequivocal biosynthetic-secretory (secretory) pathway components among the identified enhancers of GSLs’ polarity function. The analysis of all secretory pathway components collected from the initial screens revealed that SEC-23/SEC-24.1 and multiple (nine) additional secretory carrier molecules, most of them implicated in pre-Golgi trafficking and not in apical sorting, were independently required to determine the polarized distribution of apical membrane and PAR polarity complex components on expanding membranes of wild-type intestines. Conversely, the analysis of the identified GSL suppressors in wild-type membrane biogenesis uncovered polarized endocytic-recycling routes, not required to determine membrane polarity but required to supplement the GSL-dependent biosynthetic route, and revealed previously unknown functions on these routes for the small guanosine triphosphatase (GTPase) RAB-7, DAB-1/Disabled, and the V–adenosine triphosphatase (ATPase) subunit VHA-6. A secretory pathway, linked to polarized recycling routes, whose directionality is regulated upstream of membrane-based polarity cues during de novo polarized membrane biogenesis, suggested an alternative mode of membrane polarization that could address open questions of current models of epithelial polarity and polarized trafficking.

## RESULTS

### An approach to track de novo polarized membrane biogenesis in single cells in vivo

**Fig. 1.**
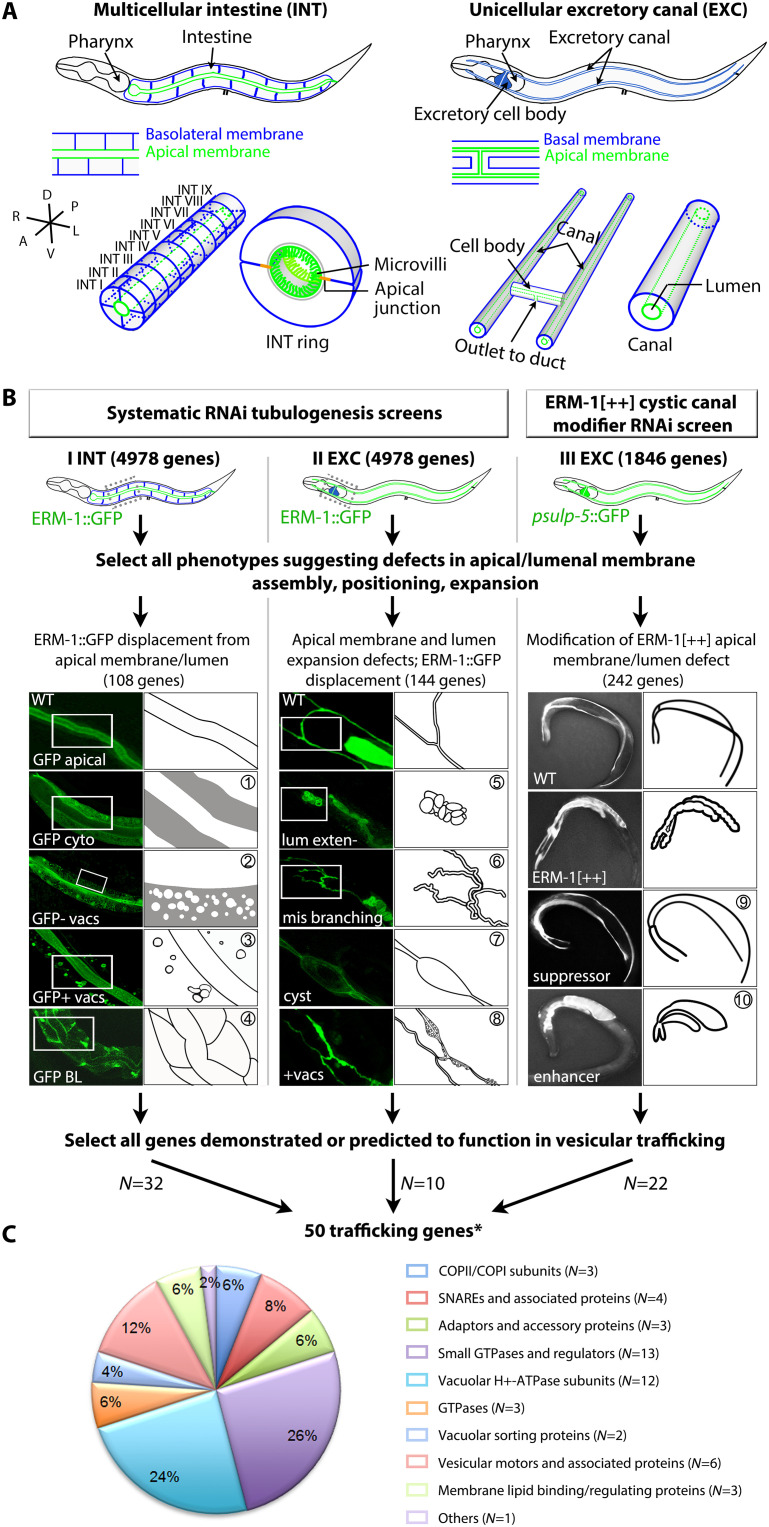
Intestinal and excretory canal tubulogenesis screens identify 50 trafficking molecules required for inTRA- or inTERcellular lumenogenesis. (**A**) Schematics of the *C. elegans* intestine (INT) and the excretory canal (EXC). INT (left): 20 cells form nine rings in bilateral symmetry, four cells in ring I. EXC (right): two anterior and two posterior canal arms extend from one single cell. Top: whole animal; middle: tube portion at higher magnification; bottom: 3D view. Here and below: apical = lumenal membrane, green; basolateral (BL) membrane, blue; anterior (A), left; posterior (P), right; dorsal (D), up; ventral (V), down; right (R), left; left (L), right. (**B**) Layout of tier-1 screens. Screens I and II first examined each gene of chromosome III for effects on INT and EXC morphogenesis in animals labeled with apical ERM-1::GFP. On the basis of the results of this pilot screen, the rest of the genome was scanned by assessing each lethal gene (total *N* = 4987; Materials and Methods) (*8*). Screen III examined putative ERM-1 interacting gene classes for their effects on the ERM-1[++] cystic EXC phenotype in animals labeled with *psulp-5*::GFP (*N* = 1846) (*28*). (I) INT screen: displacement of ERM-1::GFP [apical/lumenal in wild type (WT)] to the cytoplasm, homogeneously (GFP cyto); a vacuolized cytoplasm with ERM-1::GFP-negative vacuoles (GFP- vacs); ERM-1::GFP-positive cytoplasmic vacuoles (GFP+ vacs); the BL membrane (GFP BL) (① to ④). (II) EXC screen: ERM-1::GFP (apical/lumenal in WT) indicates no lumen extension (lum exten-), lumen branch loss/excess (mis branching), cystic lumen (cyst), and cytoplasmic GFP-positive puncta/vacuoles (+vacs) (⑤ to ⑧). (III) EXC modifier screen of ERM-1 overexpression (ERM-1[++])–induced short cystic canals: suppressor = canal re-extension, enhancer = cyst enlargement and further canal shortening, in ERM-1[++] (⑨ and ➉). Confocal images, left; corresponding schemata of boxed areas or whole animals, right. *Ten genes identified in two screens, two genes in three screens. (**C**) Pie chart: percentage distribution of functional classes (color coding maintained in figures below).

The analysis of de novo polarized epithelial membrane biogenesis under both in vitro and in and ex vivo conditions (e.g., in organoids) has been complicated by the flatness of epithelial cells and by tissue complexity, as well as by confounding effects of polarized cell migration and division that coincide with de novo polarized membrane biogenesis. To overcome these limitations, we here examined de novo polarized membrane biogenesis live in single postmitotic cells at their invariant positions within the three-dimensional (3D) (apicobasolateral) context of single-layered, expanding *C. elegans* tubular organ epithelia (Fig. 1A). We tracked ERM-1, the ancestral ortholog of the ERM/ezrin-radixin-moesin family of membrane-actin linkers that are enriched at the lumen of tubular epithelia where they denote apical membrane identity and successful apical membrane polarization (fig. S1) (*29*–*31*). In the 20-cell *C. elegans* intestine and the 1-cell excretory canal, ERM-1 delineates the coincident events of de novo apical membrane and lumen biogenesis from the early embryo (polarity establishment/initial membrane polarization and expansion; cells divide and move), through late embryogenesis and four larval stages (ongoing membrane polarization and expansion; cells no longer divide and move), to the adult (membrane polarity maintenance; no/minimal membrane expansion; see fig. S1 for tubulogenesis process) (*28*, *32*). Polarized membrane biogenesis is separable from polarized cell division and migration in late-embryonic/larval tubes, where net membrane expansion can be monitored in situ in single postmitotic cells over ~48 hours required for full tube extension in animals growing at room temperature (longer if growth is delayed; a ~34- and 2000-fold increase in apical membrane length in the intestine and canal, respectively; fig. S1, B and C).

We tracked ERM-1::GFP^+^ membranes along apicobasal cellular axes between cells during inTERcellular lumenogenesis in the intestine and along anterior-posterior cellular axes during inTRAcellular lumenogenesis inside the single canal cell. This approach permits the distinction of the development of apicobasal membrane polarity from other aspects of apical membrane biogenesis by discerning (i) apical membrane assembly (acquisition of apical membrane character; ERM-1 recruitment to the membrane from the cytoplasm), (ii) apical membrane positioning (determination of apicobasal membrane polarity; apicobasal ERM-1 distribution along the membrane), and (iii) apical membrane expansion (de novo apical membrane addition; extension of the ERM-1^+^ membrane).

### Multi- and unicellular *C. elegans* tubulogenesis screens identify 50 trafficking molecules required for apical membrane assembly, positioning, or expansion

In several RNA interference (RNAi)–based visual *C. elegans* screens (tier-1 screens; Fig. 1B, I to III), we identified a broad spectrum of intestinal and excretory canal tubulogenesis phenotypes [see Fig. 1B legend, fig. S2, Materials and Methods, and (*8*, *28*) for screen design]. We here separated the intestinal and canal lumenogenesis phenotypes (*N* = 494) from other tubulogenesis phenotypes and sorted them into 10 classes by their effects on (i) apical membrane assembly (cytoplasmic intestinal ERM-1 displacement; Fig. 1B, classes ① to ③), (ii) apical membrane positioning (basolateral intestinal ERM-1 mislocalization; Fig. 1B, class ④), and (iii) apical membrane expansion [ERM-1^+^ canal lumenal membrane extension defects (Fig. 1B, classes ⑤ to ⑧) and ERM-1–dependent canal lumen extension defects (Fig. 1B, classes ⑨ and ➉)]. Most intestinal phenotypes involved defects in apical membrane assembly (cytoplasmic ERM-1 displacement, with or without ERM-1^+/−^ vacuoles in the cytoplasm; Fig. 1B, classes ① to ③; *N* = 90 gene knockdowns). As expected, bona fide apicobasal membrane polarity defects, here narrowly defined as defects in apical membrane positioning (ERM-1 mislocalization to the basolateral membrane domain of intestinal cells; Fig. 1B, class ④), were only rarely encountered (*N* = 18 gene knockdowns).

**Fig. 2.**
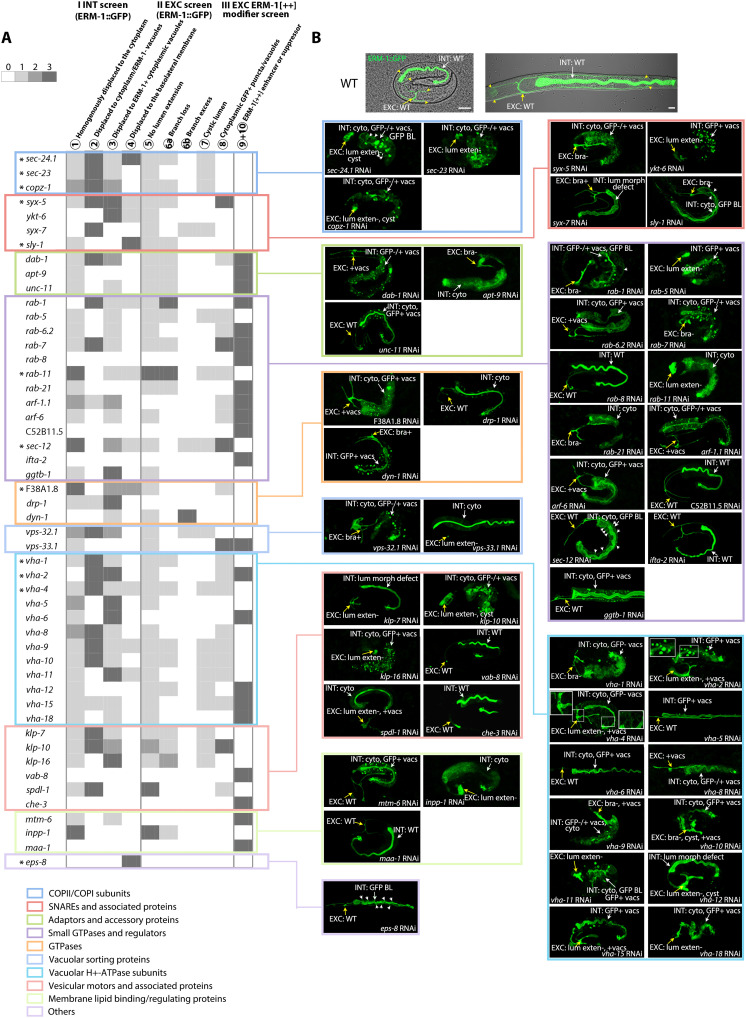
A shared set of trafficking molecules directs apical membrane assembly, positioning, and expansion within the single canal cell and in-between pairs of intestinal cells. (**A**) Fifty trafficking genes identified in tier-1 screens (I, II, and III), arranged by trafficking function (colored boxes) and phenotype class (columns). Phenotypes are distinguished as follows: none (0, no gray), weak (1, light gray), strong (2, gray), and phenotype that led to the identification of the gene (3, dark gray). Results of repeat RNAi screens are included. The list does not include genes identified by early combined tissue and membrane polarity defects or genes subsequently identified by membrane polarity defects (*sec-23, copz-1, syx-7, ggtb-1, vha-1, vha-2*). *Genes recovered in tier-2 screens as GSL enhancers. The following gene products (all phylogenetically conserved) have documented physical or functional interactions in various species: components of protein complexes: VHA-1/-2/-4/-5/-6/-8/-9/-10/-11/-12/-15/-18, SEC-23/-24.1; interacting proteins: SYX-5/YKT-6, SYX-5/SLY-1, SYX-5/SEC-24.1, SYX-5/RAB-1, YKT-6/SYX-7, RAB-1/SEC-23/SEC-24.1, DAB-1/APT-9, APT-9/ARF-1.1 (see table S1 for references). (**B**) Selection of corresponding INT/EXC embryonic/larval apical membrane assembly, positioning, and expansion defects. Not all phenotypes are shown, and not all defects are indicated (see fig. S3 legend for description of phenotypes and RNAi approach). Top panels: Nomarski/confocal overlay images of WT late (threefold) embryo (left) and late L1 larva (right). Two- to threefold late embryos are shown for most genes; L1 larvae for *ggtb-1*, *vps-33.1*, *eps-8*, *vha-5*, *vha-6*, *vha-8*; early embryos for *sec-23*, *sec-24.1*, *ykt-6* (INT and EXC tubulogenesis arrest stages can be discordant). Among early-embryonic phenotypes with morphogenesis defects, only INT intercalation defects are shown. lum morph defect: lumen morphogenesis defect; bra−/+: branch loss/excess (see Fig. 1B legend for other acronyms). Confocal projections and sections of ERM-1::GFP–labeled animals are shown. White arrows, INT; yellow arrows, EXC; white arrowheads, BL membranes; yellow arrowheads, canal tips. Scale bars, 10 μm. Color coding as in Fig. 1C.

We next selected all genes with demonstrated or predicted roles in vesicular trafficking from a total of 453 genes that were isolated by their functions in any of these aspects of apical membrane biogenesis (41 genes were identified by more than one of the identified phenotype classes). From among these 453 genes, 50 genes were identified that encoded proteins implicated in vesicular trafficking, corresponding to ~3% of annotated *C. elegans* trafficking genes [Wormbase release WS267; Fig. 1C and table S1; Materials and Methods; note that genes encoding GSL-biosynthetic enzymes and clathrin/AP-1 subunits, previously identified in these screens (*8*, *12*), were not included here]. All these gene products are phylogenetically conserved by structure, location on the endomembrane/vesicle membrane, and trafficking function. Twenty-six of 50 gene products belong to functional classes with members implicated in directional trafficking and polarized sorting in diverse species, e.g., Rab GTPases/Rab-related (*N* = 13, the largest subgroup; RAB-5/Rab5, RAB-8/Rab8, RAB-11/Rab11 with documented functions in apical domain positioning) (*20*, *33*, *34*), motors (*N* = 6; KLP-16/KIFC3 with documented functions in apical transport) (*35*), v-/t-SNAREs (*N* = 4), and coat adaptors (*N* = 3) (table S1, Figs. 1C and 2A, and legends). The second largest subgroup consists of 12 subunits of V-ATPases, endomembrane- and plasma membrane–based proton pumps (*36*), recently also implicated in apical secretion and polarized tubulogenesis (*37*–*39*). Validating screen specificity and consistent with genome coverage, multiple subunits of protein complexes and of proteins previously shown to functionally or physically interact were independently identified by similar phenotypes (see Fig. 2A legend and table S1 for references). All 50 genes are expressed in the intestine or excretory canal by RNA sequencing (*40*) or other experimental approaches (table S1).

Since these 50 genes were isolated from different screens (intestinal or canal screens) and by different phenotypes, we carried out a repeat RNAi analysis of all 50 genes (fig. S2) to examine the genes’ effects on both tubes (intestine and canal), all aspects of apical membrane biogenesis (phenotype classes ① to ➉), and all developmental stages (note that most knockdowns are sterile and/or embryonic/early-larval lethal). Further validating screen quality, this analysis revealed unexpected common properties among the genes’ functions: Most genes operated in (i) both intestinal and canal lumenogenesis (42 of 50 genes; Fig. 2B, fig. S3, and table S1) and in similar aspects of apical membrane biogenesis in these different tubes (Fig. 2A for phenotype signatures across classes ① to ➉); (ii) multiple aspects of apical domain biogenesis in each tube (43 of 50 genes; Fig. 2A) [notably, some genes identified by their requirement for apical membrane expansion in the canal were also found to be required for apical membrane positioning (polarity) in the intestine (Fig. 1B, class ④)]; and (iii) both embryonic and larval development, i.e., during and after completion of polarized tissue (tube) morphogenesis (44 of 50 genes; Fig. 2, fig. S3, and table S1).

In summary, tier-1 screens identify 50 trafficking molecules required for apical domain and lumen morphogenesis in multicellular or unicellular tubular *C. elegans* epithelia. The comparative analysis of these molecules reveals that a shared trafficking machinery supports the different tissue-morphogenetic processes of inTERcellular/intestinal and inTRAcellular/canal lumenogenesis and the distinct membrane-biogenetic processes of apical membrane assembly (acquisition of apical membrane character), apical membrane positioning (determination of apicobasal membrane polarity), and apical membrane expansion (net addition of newly synthesized apical membrane). All genes function in growing epithelia, i.e., during de novo apical membrane and lumen biogenesis, and most function in both embryonic cells that still divide and move and in larval cells that no longer divide and move but still expand.

### Genetic modifiers of the GSL-dependent apicobasal polarity conversion identify multiple trafficking molecules that determine membrane polarity

**Fig. 3.**
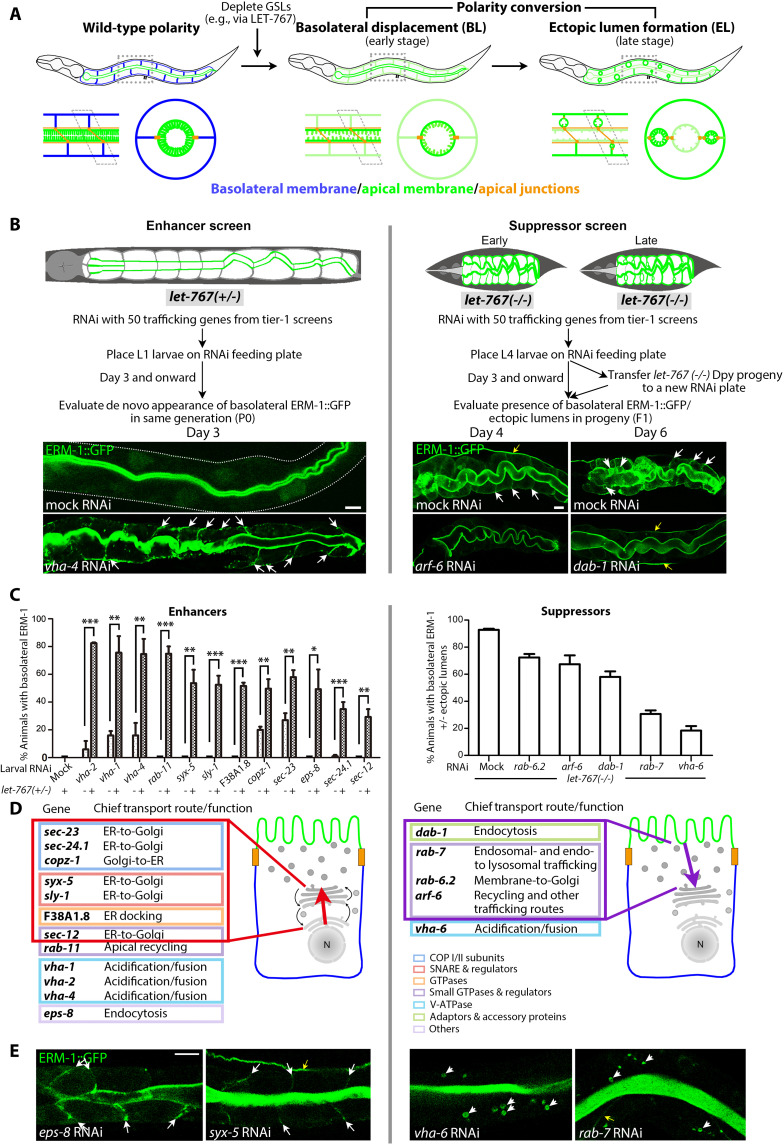
Enhancers and suppressors of the GSL-dependent apicobasal polarity conversion. (**A**) Schematics of in vivo apicobasal polarity conversion. Top: whole larva; bottom: longitudinal (left) and transverse section (right) through one INT ring. BL displacement of apical membrane components precedes BL ectopic lumen (EL) formation. Note the loss of apical versus gain of BL microvilli and intact apical, but new BL lumen-surrounding junctions, indicating full structural apical membrane, membrane-microdomain, and membrane-domain formation at (prior) BL membrane (*8*) (compare fig. S6A). (**B**) Genetic interaction screens (compare text). Top: schematics, left: *let-767(+/−)* larva (normal body morphology); right: L1-arrested *let-767(−/−)* larvae [dumpy (Dpy) morphology]. ERM-1::GFP identifies apical membrane (green). Middle: screen layout. Enhancer screen (left): phenotype is evaluated in the same generation, with enhancement required to induce BL ERM-1 mislocalization in WT-appearing *let-767(+/−)/RNAi* INT; suppressor screen (right): phenotype is evaluated in the next generation and requires prevention of phenotype development or reversion of the full phenotype (including BL ERM-1, ELs, L1-arrest) in *let-767(−/−)/RNAi* progeny. Bottom images: representative examples of enhancement (left) and suppression (right). Note the punctate nature of BL ERM-1 mislocalization. (**C** to **E**) Screen results (enhancers left, suppressors right). (C) Quantification of enhancement and suppression. Mean ± SEM is shown (*n* = 3/*N* > 50). One-tailed Student’s *t* test was used to calculate *P* values. (D) Schematics: genetic modifiers arranged by conserved primary site of function/route: enhancers are enriched for pre-Golgi components of anterograde secretory routes (red rectangle/arrow), suppressors for post-Golgi components of retrograde endocytic-recycling routes (purple rectangle/arrow). *eps-8*, an apical INT brush border component (*108*), is listed by its conserved function in endocytosis (*109*), but could also function in polarity via actin. (E) Representative images of membrane-positional (BL) ERM-1 mislocalization (enhancers) and cytoplasmic vacuolar ERM-1 mislocalization (suppressors) in WT INTs. White arrows: BL mislocalization; arrowheads: ELs in (B)/cytoplasmic vacuoles in (E). Yellow arrows: EXC. Scale bars, 5 μm.

We next used these 50 trafficking molecules, identified by their requirement for apical membrane biogenesis, to ask whether the GSL-dependent apicobasal polarity conversion in the *C. elegans* intestine was mediated by vesicular trafficking and, if so, by which trafficking route (see Introduction). GSL depletion results in the basolateral displacement of all components of the apical domain (and lumen), with subsequent ectopic basolateral lumen and junction formation (Fig. 3A), and ERM-1::GFP tracks this full membrane domain identity change (*8*). We carried out genetic enhancer and suppressor screens in animals labeled with ERM-1::GFP (tier-2 screens) and examined the ability of each of the 50 tier-1 trafficking genes to modify ERM-1’s apical membrane position in a GSL-biosynthetic enzyme–deficient mutant (*let-767[s2819]*; Fig. 3B and fig. S2). We defined enhancement as ERM-1 emergence at basolateral domains in initially wild-type–appearing intestines that were only weakly sensitized by the loss of one *let-767* copy (*let-767[s2819]* animals carrying the free duplication *sDp3* that provides one *let-767* copy, designated *let-767[+/−]* for simplicity). RNAi was induced post-embryogenesis, requiring enhancers to directly affect polarized membrane biogenesis, independent from polarized tissue morphogenesis (see above, approach, and fig. S1). We defined suppression as the prevention/reversion of basolateral ERM-1 displacement, ectopic lumen formation, and/or arrest/lethality in the absence of both *let-767* copies (*let-767[s2819]* animals without *sDp3*, designated *let-767[−/−]* for simplicity; animals appear “dumpy” due to a mutation uncovered by the loss of *sDp3*). RNAi was induced before embryogenesis, requiring suppressors to rescue/revert the fully expressed phenotype.

These tier-2 screens recovered an unexpectedly large percentage of tier-1 trafficking molecules as modifiers of the GSL-dependent apicobasal polarity change (17 of 50; Fig. 3C and figs. S2 and S4, A and B), supporting the hypothesis that GSLs regulate membrane polarity via trafficking and underscoring GSLs’ critical contribution to such a trafficking-based polarity function. However, only a few of these 17 trafficking molecules came from functional classes previously implicated in apical sorting or other aspects of directional trafficking in *C. elegans* or other species (only 6 of 26 such molecules identified in tier-1 screens: e.g., 3 of 13 Rabs/Rab-related and 0 of 6 motors), whereas most had not yet been implicated in polarized trafficking (e.g., 5 of 6 vesicle coat/coat assembly components; see color-coded boxes in Figs. 1C, 2A, and 3D).

The 12 GSL enhancers and the 5 GSL suppressors fell into different classes based on their conserved wild-type trafficking functions (Fig. 3D and table S1). Notably, enhancers, whose mild depletion newly mispositions the apical domain in a haplosufficient *let-767(+/−)* background (Fig. 3B), were enriched for secretory pathway components of membrane-directed (anterograde) vesicle trajectories (7 of 12). This result implicated the secretory pathway in the regulation of membrane polarity and suggested that GSLs position the apical domain on anterograde secretory or parallel trafficking routes. Validating this finding, the enhancer screen also identified conserved molecules previously implicated in apical domain positioning in different species via apical recycling or by functions at the apical membrane–vesicle interface [RAB-11, VHA-1/4, and EPS-8, an intestinal brush border component that could also function in polarity via actin (*20*, *34*, *39*, *41*, *42*); note that clathrin/AP-1, previously characterized as GSL enhancers (*12*), are not included in this analysis]. In contrast, suppressors, whose strong depletion reduces apicobasal polarity conversion in a *let-767(−/−)* background (Fig. 3B), were enriched for components of endocytic-recycling and vesicle-degradative trajectories leading away from the membrane (retrograde trafficking routes; Fig. 3D and table S1). Of note, V-ATPase subunits were identified among enhancers and suppressors, suggesting specific yet distinct roles of pump subunit isoforms in membrane polarity [subunit isoforms can assemble in an organelle-specific manner (*36*); see the VHA-6 analysis below and fig. S7A legend for discussion].

Enhancers and suppressors also fell into different classes when examined in a wild-type background. Hierarchical clustering of the 50 tier-1 knockdowns by their phenotype profiles in wild type could predict whether they would enhance or suppress GSLs’ polarity function (fig. S4C). The strongest predictor for enhancement was a combined defect in wild-type apical membrane assembly and positioning (combined cytoplasmic and basolateral ERM-1 displacement; 8 of 12 enhancers, 0 of 5 suppressors). An extended analysis of polarized membrane biogenesis in expanding postmitotic larval cells (Materials and Methods) revealed that depleting each enhancer (12 of 12) was able to mislocalize ERM-1 to basolateral domains, whereas depleting each suppressor (5 of 5) displaced ERM-1 to cytoplasmic vacuoles or had no effect (Fig. 3E). We concluded that all trafficking molecules interacting with GSLs in membrane polarity were themselves required for apical membrane assembly, but only enhancers for apical membrane positioning and hence for the regulation of apicobasal membrane polarity.

In summary, GSL enhancers discover multiple previously unidentified trafficking molecules that determine apicobasal membrane polarity by specifying the position of the apical domain on expanding *C. elegans* intestinal membranes, most of them secretory molecules, while confirming the conserved role of several endocytic-recycling molecules in apical domain positioning. In contrast, GSL suppressors, most operating on endocytic and vesicle-degradative routes, identify trafficking molecules required for the GSL-dependent polarity conversion but are dispensable for apical domain positioning in wild type. We conclude that the trafficking molecules that enhance and suppress the GSL-dependent polarity conversion are distinguished by their (i) primary site of function in wild type (antero- versus retrograde trafficking routes) and (ii) role in wild-type apical membrane biogenesis (apical membrane positioning versus assembly). This constellation suggested enhancer- and suppressor-specific distinct mechanisms of action and interaction with GSLs in membrane polarity that might involve trafficking directions. Notably, these sensitized tier-2 screens identify multiple genes encoding unequivocal components of the secretory pathway that determine apicobasal membrane polarity [*sec-23/24.1*, *copz-1*, *syx-5*, *sly-1*, F38A1.8, *sec-12* (*43*, *44*); table S1 for gene annotation and references].

### The secretory pathway determines the polarized distribution of apical membrane and PAR complex components during de novo membrane biogenesis

The secretory pathway delivers biosynthetic cargo from the endoplasmic reticulum (ER) via pre-Golgi, Golgi, and post-Golgi endomembranes/vesicle membranes to all sides of the epithelial plasma membrane (*5*). Its apicobasal directionality is thought to be determined by polarized cargo signals that are decoded only on the level of Golgi- and post-Golgi endomembranes (e.g., by GSLs) to sort post-Golgi vesicles to cognate recognition sites at corresponding target membranes (e.g., the apical domain) (*5*, *44*). Pre-Golgi (early) pathway components are therefore not expected to specify this cargo’s apicobasal distribution at the membrane. Yet, interference with each of seven, predominantly early pathway components (the COPI/II coat/coat assembly components SEC-23, SEC-24.1, SEC-12, and COPZ-1; the SRPR component F38A1.8; the t-SNARE SYX-5 and its assembly factor SLY-1), enhances the GSL-dependent apicobasal polarity conversion and shifts ERM-1 to basolateral domains of expanding membranes in the *C. elegans* intestine (Fig. 3, C to E; fig. S4A; and table S1). We weighed possible consequences of early (pre-Golgi) secretory pathway disruption that might explain these unexpected findings: (i) nonspecific effects on secretion or secondary effects on apical delivery and (ii) compensatory mechanisms in downstream (post-Golgi) trafficking (e.g., increased basolateral delivery). Possibility (i) was unlikely since it should (a) displace ERM-1 to the cytoplasm only, not to the basolateral membrane, irrespective of which delivery route might be affected (direct, indirect, or transcytotic) and (b) reduce cargo flux through the pathway and thus suppress, not enhance, apicobasal polarity conversion (see the suppressor analysis below). Possibility (ii) was also unlikely since post-Golgi trafficking routes depend on upstream (pre-Golgi) trafficking. We, therefore, concluded that either multiple early secretory pathway components have as-of-yet unknown functions in apical sorting or that the secretory pathway affects membrane polarity by some other, not-yet characterized mechanism.

**Fig. 4.**
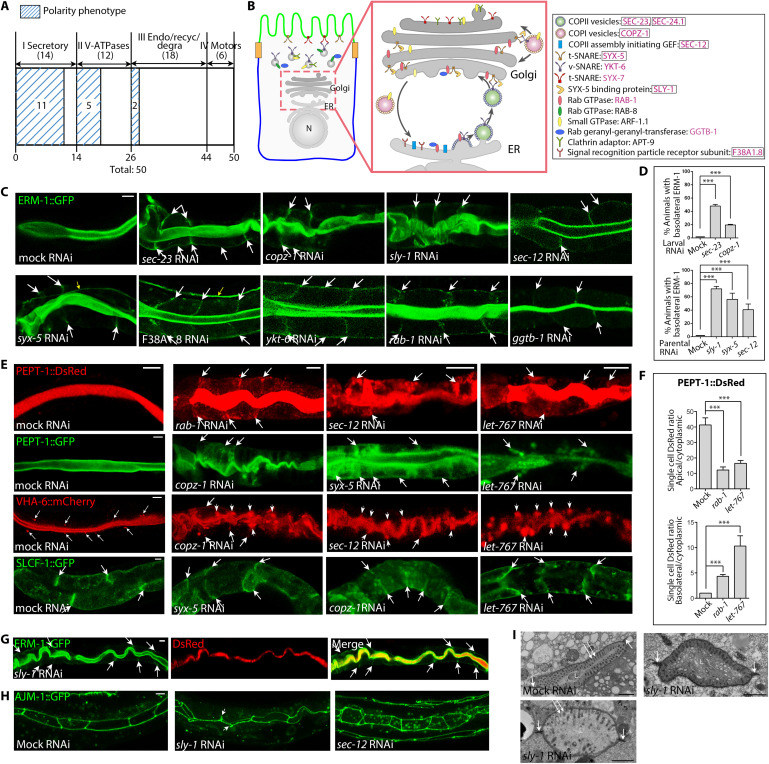
Interference with each of multiple secretory pathway components misdirects apical membrane components to basolateral domains during de novo polarized membrane biogenesis. (**A**) Among 50 trafficking molecules identified in tier-1 screens, polarity defects track with the loss of secretory molecules. Molecules are classified by their conserved trafficking functions/routes (Endo, endocytosis; recyc, recycling; degra, degradation). (**B**) Endomembrane location of 14 secretory pathway components identified by their requirement for apical domain biogenesis. Components required for WT membrane polarity are shown in pink; enhancers of GSLs’ polarity function are boxed. (**C** and **D**) Mild interference with each of multiple secretory molecules misdirects the apical domain identity marker ERM-1 to BL membranes (arrows) in expanding larval INTs. Yellow arrows: EXC. (D) Quantification of polarity defects subsequent to RNAi induced in same (top) or parent generation (bottom). Mean ± SEM is shown (*n* = 3/*N* > 50). One-tailed Student’s *t* test was used to calculate *P* values. (**E** and **F**) Effects of mild secretory pathway perturbation on the polarized distribution of integral membrane components in larval INT cells. Arrows: BL membrane; short arrows: BL membrane–aligned vacuoles; thin arrows: small VHA-6^+^ subapical vesicles. Defects copy polarity conversion induced by *let-767* mild RNAi (BL ERM-1, no ELs) (*8*). (F) Quantification of PEPT-1::DsRed fluorescence intensity ratios. Mean ± SEM is shown (*n* = 3/*N* > 10). Two-tailed (top) or one-tailed (bottom) Student’s *t* tests was used to calculate *P* values. (**G** to **I**) Apical junction integrity is not affected by *sly-1* and *sec-12* mild RNAi. (G) Larvae fed with DsRed-labeled bacteria retain the label in the INT lumen despite BL ERM-1 mislocalization (arrows). (H) The junction integrity molecule AJM-1 remains contiguous (short arrows: occasional lateral broadening). (I) TEM images of *sly-1(mildRNAi)* deformed INT lumens (L): intact junctions (short arrows); reduced/absent microvilli number/length (long arrows); paucity and dissolution of endomembranes. Upper image: mild depletion, lower image: stronger depletion; fig. S5 for full INTs. Scale bars, 5 μm and 1 μm in (I).

To further investigate the secretory pathway’s function in polarity, we reexamined all pathway components that had been identified in tier-1 screens by their requirement for apical membrane domain biogenesis (14 of 453; fig. S2). Although only seven such components were recovered as GSL enhancers in tier-2 screens (itemized above), three more (YKT-6, SYX-7, and GGTB-1) displayed an enhancer-specific phenotype signature in wild type (fig. S4C). An extended analysis of apical membrane expansion (Materials and Methods) in knockdowns of these 14 pathway components confirmed YKT-6, SYX-7, GGTB-1, and identified RAB-1 as additional cues for apical membrane positioning. Among different classes of tier-1 trafficking molecules, apicobasal polarity defects thus tracked with the loss of secretory pathway components (Fig. 4A and table S2). In total, we identified 14 such components, with conserved sites of function ranging from ER, pre-Golgi, Golgi, and post-Golgi endomembranes to the plasma membrane (Fig. 4B); 11 were required for apical membrane assembly and positioning in the intestine (Fig. 4B, pink), and 7 in conjunction with GSLs (Fig. 4B, pink/boxed). All 14 directed apical membrane expansion between pairs of cells (intestine) and inside one cell (excretory canal). We concluded that, during de novo polarized membrane biogenesis, the secretory pathway harbors directional (apical) cues along its path from the ER to the plasma membrane and concomitantly positions, assembles, and expands the apical domain into the growing membrane. Hence, at the time of net polarized membrane addition, the secretory pathway’s directionality appeared to be predetermined from the ER toward the nascent apical domain.

Full disruption of the secretory pathway aborts all membrane-directed traffic, making it impossible to detect membrane polarity defects caused by a putative change in the secretory pathway’s directionality. To analyze the pathway’s polarity function, we used a scaled-intensity RNAi approach and mildly depleted single pathway molecules. Mild *sec-23*, *copz-1*, *sly-1*, *sec-12*, *syx-5*, F38A1.8, *ykt-6*, *rab-1*, and *ggtb-1* RNAi each mislocalized ERM-1 to basolateral sides of expanding membranes in postmitotic late-embryonic and larval intestinal cells (Fig. 4C and fig. S1). RNAi induced in L1 larvae (ongoing net membrane expansion; fig. S1) was sufficient to change the position of the ERM-1^+^ apical membrane domain, whereas RNAi induced in adults (no/minimal membrane expansion) had no detectable effect on the position of the apical domain (Fig. 4, C and D). These results suggested that the secretory pathway’s role in apical membrane positioning is (i) a direct function of polarized membrane biogenesis (still effective after completion of polarized tissue morphogenesis), (ii) developmentally regulated (restricted to the embryonic and larval epithelium), (iii) dependent on net membrane addition (here only demonstrated in the expanding, not the mature, epithelium).

*sec-23*, *sec-12*, *syx-5*, *sly-1*, and *copz-1* mild RNAi also mislocalized other apical membrane components (submembranous actin/ACT-5 and intermediate filaments/IFB-2; the integral membrane components PEPT-1 and VHA-6, a V-ATPase subunit also identified as a GSL suppressor; see below) to the basolateral domain, the cytoplasm, and cytoplasmic vacuoles (Fig. 4, E and F). In contrast, the same RNAi conditions only modestly displaced the basolateral integral membrane protein SLCF-1 to the cytoplasm, without misdirecting it to the apical domain (Fig. 4E). Apical junction integrity remained intact in these intestines that were only mildly depleted of secretory pathway components, suggesting that junction leakage did not cause the polarity change. However, ectopic junction material could be observed in the cytoplasm and at lateral membranes (Fig. 4, G to I; fig. S5; and movie S1). We concluded that the secretory pathway’s role in the polarized distribution of membrane components is not limited to ERM-1 but specific to apical membrane, including apical junction, components.

**Fig. 5.**
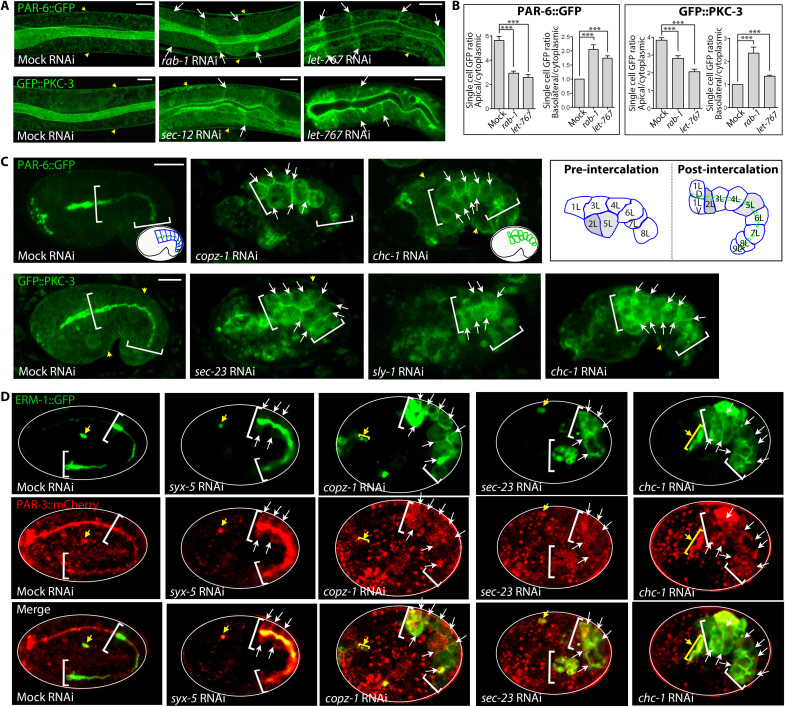
Interference with the secretory pathway misdirects apical PAR complex components to basolateral domains and prevents PAR polarization during polarity establishment. (**A** and **B**) The recruitment of apical PAR complex components to, and their polarized distribution at, the expanding membrane depends on the secretory pathway. (A) PAR-6 and PKC-3, strictly apical in WT (mock), are mislocalized to the cytoplasm and to BL membranes (some indicated by arrows, here and below) in *rab-1 and sec-12(mildRNAi)* larval INT cells [*let-767* mild RNAi is shown for comparison (*8*)]. Yellow arrows: EXC. Confocal sections of two to three cell pairs surrounding the lumen are shown. (B) Quantification of PAR-6::GFP and GFP::PKC-3 fluorescence intensity ratios. Mean ± SEM is shown (*n* = 3/*N* > 10). Two-tailed (left) and one-tailed (right) Student’s *t* tests were used to calculate *P* values. (**C** and **D**) The polarization of apical PAR complex components during intestinal polarity establishment depends on the secretory pathway. (C) Failure of PAR-6 and PKC-3 polarization in early-embryonic *copz-1*, *sec-23*, *sly-1(RNAi)* INTs [cytoplasmic and BL (arrows) mislocalization; *chc-1/clathrin* RNAi is shown for comparison (*12*)]. Note concomitant INT intercalation defects. Insets show cartoons of polarized (mock: PAR-6 apical) and nonpolarized (*chc-1* RNAi: PAR-6 pan-membranous) post-intercalation INTs of embryos within the eggshell. Brackets: INT; yellow arrows: hypodermis. Schematic: pre- and post-intercalation INT [compare fig. S1A: during intercalation, a pair of lower-tier cells moves apical into an upper tier, in parallel on the left (L) and the right side]. (D) Failure of PAR-3 polarization in early-embryonic *syx-5*, *copz-1*, *sec-23*, and *chc-1(RNAi)* INTs (concomitant failure of ERM-1 polarization shown for clarity in double-labeled strain). Note that apical PARs are directly recruited to, and remain at, the apical/apicolateral domain during WT polarity establishment. Confocal sections of whole embryos are shown. Eggshell is outlined by white line. Brackets: INT; yellow arrows: EXC. Scale bars, 5 μm.

We considered that a secretory pathway whose directionality was predetermined during de novo polarized membrane biogenesis might operate upstream of membrane-based apical polarity cues and also initiate membrane polarization during polarized tissue morphogenesis. Stronger interference with multiple pathway components prohibited membrane polarization in the intercalating early-embryonic intestine, at the time when its definitive polarity and lumen position are established (fig. S1A): In still dividing and moving early-embryonic cells, ERM-1 was either fully displaced to the cytoplasm (with and without ERM-1^+/−^ vacuoles; Fig. 2B: e.g., *sec-23*, *copz-1*, *syx-5*, and *ykt-6*) or, if membrane-associated, nonpolarized (pan-membranous ERM-1; Fig. 2B and fig. S3: e.g., A and J; note concomitant intestinal intercalation arrest and absence of intestine and canal lumen formation, processes that depend on the polarization of the apical membrane) (*45*, *46*). Furthermore, *sec-23*, *sec-12*, *syx-5*, *copz-1*, *sly-1*, or *rab-1* RNAi not only mislocalized PAR-6 and PKC-3 to the cytoplasm and to basolateral domains in late-embryonic/larval cells (Fig. 5, A and B) but also prevented the polarization of all three apical PAR complex components, including the earliest polarity cue PAR-3, during polarity establishment in the early-embryonic intestine (Fig. 5, C and D; PAR-3 expression weans at the late-embryonic/larval stage). We concluded that the secretory pathway determines the polarized distribution of apical membrane and PAR complex components on the expanding membrane, whether or not this membrane is polarized. The pan-membranous distribution of apical PARs in early-embryonic cells with impaired (but not fully disrupted) secretion (Fig. 5, C and D) revealed that it is the secretory pathway’s ability to confer polarized (apical), not only anterograde (membrane-directed), directionality to trafficking that determines membrane polarity during polarity establishment (note that, during wild-type polarity establishment, apical PAR complex components are directly recruited to the future apical/apico-lateral membrane domain, without prior pan-membranous distribution) (*47*–*49*).

**Fig. 6.**
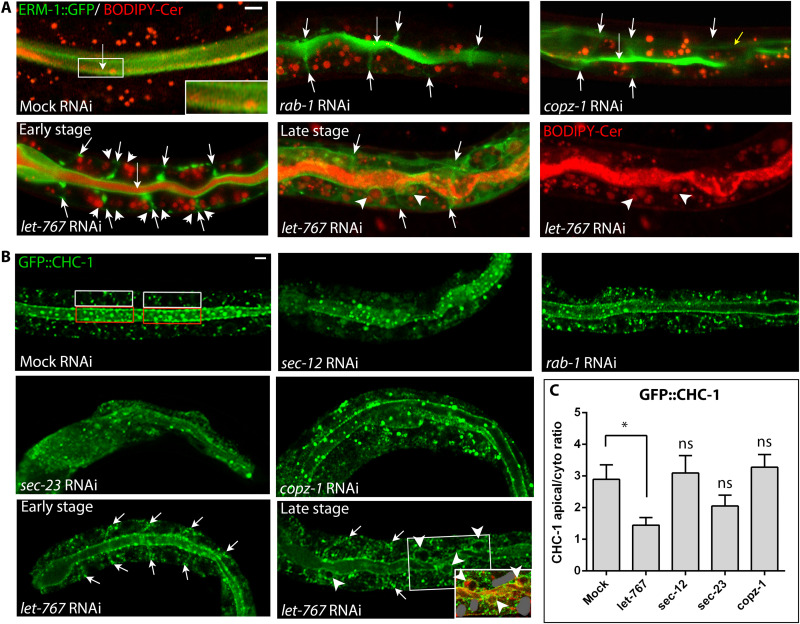
Effects of early secretory pathway perturbation on post-Golgi vesicle positioning. (**A**) Defects in GSL-rich [BODIPY-Cer^+^] post-Golgi vesicle biogenesis but not positioning. Upper row: unchanged WT (mock) pan-cytoplasmic distribution of BODIPY-Cer^+^ vesicles but reduction in number and increase in size in *rab-1* and *copz-1(mildRNAi)* INT cells. Note that BODIPY-Cer also labels the outer leaflet (=intralumenal portion) of the apical membrane in WT (enlarged in inset), missing in *rab-1* and *copz-1(mildRNAi)* INTs. Lower row: BODIPY-Cer^+^ vesicle positioning changes in *let-767(RNAi)* INT cells are shown for comparison (*12*): Cer^+^ vesicles are displaced to both sides of the ERM-1^+^ BL domain during early-stage polarity conversion, and into ERM-1^+^ ELs during late-stage polarity conversion [note that BODIPY-Cer also labels the outer membrane leaflet (=intralumenal portion) of ELs]. Long arrows: apical/lumenal membrane; short arrows: BL ERM-1 mislocalization; very short arrows: Cer^+^ vesicles; arrowheads: ERM-1^+^ ELs. (**B**) Defects in clathrin-coated (GFP::CHC-1^+^) post-Golgi vesicle biogenesis but not positioning. Upper two rows: sustained WT (mock) pan-cytoplasmic distribution and reduction in number of GFP::CHC-1^+^ vesicle in *sec-12*, *rab-1*, *sec-23*, and *copz-1(mildRNAi)* cells. WT apical enrichment is sustained, although diminished (note that minor positional changes could be obscured by the reduced number of vesicles). Lower row: GFP::CHC-1^+^ vesicle positioning changes during *let-767(RNAi)* polarity conversion are shown for comparison (*12*): BL displacement (arrows) during early-stage, displacement to ELs (arrowheads) during late-stage polarity conversion. Inset: ERM-1::mCherry double labeling reveals the apical character of EL membranes (additional ERM-1::mCherry is grayed out for clarity). (**C**) Quantification of GFP::CHC-1 apical membrane/cytoplasm ratio. Two areas (60 μm^2^) per INT cell were counted for the apical membrane [red box in (B)] and the cytoplasm (cyto) [white box in (B)]. Ten worms were used for the calculation. Mean ± SEM is shown. Two-tailed Student’s *t* test was used to calculate *P* values. Scale bars, 5 μm.

The early (pre-Golgi) secretory pathway is considered a prerequisite for the biogenesis of post-Golgi vesicles, but not thought to instruct their directionality (*44*, *50*). The enrichment of polarized cargo en route to the plasma membrane makes it difficult to visually track this cargo’s vesicle-based directional delivery from the ER to the plasma membrane (*5*, *10*). To further examine if nonspecific or secondary effects of early pathway disruption might have changed post-Golgi vesicle directionality, we examined the positioning of clathrin-coated and GSL-rich vesicles, previously shown to change positions from apical to basolateral domains during polarity conversion induced by interference with post-Golgi trafficking (*12*). Mild *sec-23*, *sec-12*, *copz-1*, and *rab-1* RNAi reduced the number of these vesicles but left their polarized distribution remarkably intact, despite ERM-1’s basolateral displacement (Fig. 6). Thus, mild perturbation of the early secretory pathway, strong enough to alter membrane polarity but not so strong as to collapse the dependent endomembrane system, affects post-Golgi vesicle biogenesis but not directionality. This finding is consistent with the current model of polarized trafficking that restricts directional cues to Golgi and post-Golgi endomembranes, but it also fits an alternative model where both pre- and post-Golgi endomembranes harbor directional cues that guide the secretory pathway from the ER to the apical domain during this domain’s biosynthesis.

We conclude that the secretory pathway determines membrane polarity by positioning apical membrane and PAR complex components on expanding *C. elegans* intestinal membranes from the time of the membrane’s initial polarization (polarity establishment) throughout further net polarized membrane addition required to maintain this polarity in the developing epithelium. The 11 identified pathway components that determine the polarized distribution of apical membrane components have conserved sites of action ranging from the ER to the plasma membrane and are enriched for pre-Golgi vesicle coat and coat assembly components, none implicated in apical sorting. Together, these findings suggest that this anterograde vesicle trajectory that supplies all sides of the membrane acquires apical directionality during de novo polarized membrane biogenesis, with secretory carriers being routed from the ER to the plasma membrane to asymmetrically insert the apical domain into the growing membrane [see our accompanying article (*51*)].

### Apicobasal polarity conversion is suppressed by decreasing the basolateral mislocalization of apical membrane components

**Fig. 7.**
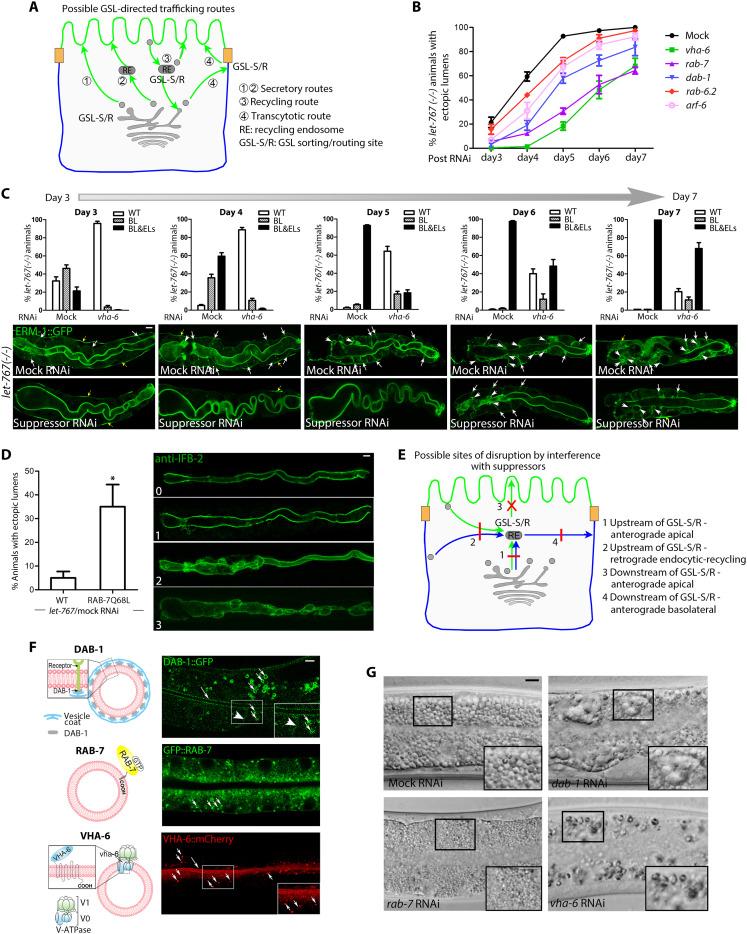
VHA-6, RAB-7, DAB-1, ARF-6, or RAB-6.2 depletion suppresses the GSL-dependent apicobasal polarity conversion. (**A**) Possible GSL-directed post-Golgi trafficking routes to the apical membrane: ① direct; ② indirect: i.e., passing through recycling, sorting, or other endosomes [recycling endosome (RE) shown for simplicity]; ③ recycling: could pass through different endosomes or Golgi/ER compartments (retrograde recycling); ④ transcytotic. GSL-S/R: GSL sorting or routing site. (**B**) Suppression of polarity conversion, expressed as percent *let-767(−/−)/suppressor(RNAi)* larvae with ELs over time. (**C**) Depletion of suppressors reduces polarity conversion but does not induce polarity reversion (VHA-6 shown as example). Bar graphs: percentage of animals with WT, early-stage (BL ERM-1; arrows), and late-stage (ELs; arrowheads) polarity conversion in *let-767(−/−)* versus *let-767(−/−)/vha-6(RNAi)* larvae over time; representative images for each time point beneath (yellow arrows: EXC). Mean ± SEM is shown (*n* = 3/*N* > 50). (**D**) Activated RAB-7 enhances polarity conversion. Left: increase in the number of *let-767(mildRNAi)* animals with INT ELs in the presence of a RAB-7Q68L transgene. Right: increase in EL number and size per animal (progression was graded from 0 to 3). IFB-2/intermediate-filament, like ERM-1, marks the INT lumen. Mean ± SEM is shown (*n* = 3/*N* = 20). One-tailed Student’s *t* test was used to calculate *P* values. (**E**) Possible sites of disruption of post-Golgi trafficking routes by interference with suppressors. GSL-S/R site arbitrarily placed at RE. Routes are colored (green: apical; blue: basolateral) according to destination, not cargo. (**F**) Subcellular localization of DAB-1, RAB-7, and VHA-6 in WT larval INTs. Schematics (left): relation of molecules to the membrane (vesicle membranes are shown). Images (right): in vivo localization on vesicles and on expanding plasma membranes in larval INTs (long arrows: apical membranes, arrowheads: BL membranes; short arrows: vesicles). (**G**) Vesicle biogenesis defects in *dab-1*, *rab-7*, *vha-6(RNAi)* larval INTs: vacuolization and vesicle aggregation, vesicle size reduction, and reduced numbers of vesicles, respectively. Scale bars, 5 μm.

In an attempt to map the route of a secretory pathway that is oriented toward the nascent apical domain during de novo polarized membrane biogenesis, we turned to the GSL suppressors. The enhancer screen had placed GSLs themselves onto, or in parallel to, the secretory pathway’s apical trajectory by their convergent function in apical membrane positioning (Fig. 3D). The small GTPases RAB-6.2, ARF-6, and RAB-7, the clathrin adaptor DAB-1/Disabled, and the V-ATPase subunit VHA-6 were all identified as loss-of-function suppressors of apicobasal polarity conversion induced by GSL depletion (Fig. 3, B and C). If these five GSL suppressors were effectors of GSL-dependent apical trafficking, reducing their activity should increase apical transport (e.g., remove an inhibition) that might reveal the secretory route’s directional regulation, delineate its itinerary, and identify sites of GSL-dependent cargo sorting or vesicle routing (Fig. 7A). However, the identification of these five suppressors in tier-1 screens by their requirement for apical membrane biogenesis predicted that reducing their activity should, conversely, decrease apical transport.

To address this apparent paradox, we first determined when and where the suppressors operate during polarity conversion and reversion [polarity conversion proceeds through stages 1 (basolateral ERM-1 mislocalization) and 2 (ectopic basolateral lumen formation); reversion proceeds in the reverse order; fig. S6A]. Monitoring membrane expansion in double *let-767(−/−)*/suppressor*(RNAi)* intestines over 7 days (fig. S6B for timing and sequence of events in GSL-deficient growth-delayed L1-larval intestines) demonstrated that RNAi with each suppressor reduced the number of *let-767(−/−)* animals with stage 1 and 2 polarity conversion and delayed its progression but failed to restore wild-type polarity and viability (Fig. 7, B and C, and table S3 for *N* of all five suppressors). The time course of phenotype suppression in double *let-767(−/−)*/suppressor*(RNAi)* intestines [a successive decrease of wild-type polarity and the corresponding increase of early-stage (basolateral ERM-1 mislocalization), followed by late-stage (ectopic basolateral lumen formation), polarity conversion] suggested that polarity conversion was suppressed by reducing the basolateral misrouting of apical membrane components (e.g., via route 3, retrograde recycling, or route 4, basolateral secretion; Fig. 7A), rather than by removing an inhibition on apical trajectories (e.g., on route 1 or 2; Fig. 7A). This scenario would also resolve the apparent paradox of the suppressors’ concomitant requirement for apical transport, suggested by their identification in tier-1 screens.

Several suppressors have conserved functions on endocytic and vesicle-degradative routes. To assess whether suppression might have been caused by nonspecific disruption of these processes, we depleted various molecules, previously shown to function on these routes in the *C. elegans* intestine, in a *let-767(−/−)* background (fig. S6C). Depletion of most of these molecules failed to suppress the *let-767*–dependent polarity conversion but identified two more suppressors (the AP-2 adaptor DPY-23 and the ARF-like GTPase ARL-8), implicating the disruption of specific endocytic routes, but not the general disruption of endocytosis or vesicle degradation, in the mechanism of suppression. We also failed to find evidence for a suppressor-dependent loss of lysosomal GSL degradation that might have been expected to suppress polarity conversion by increasing GSL levels (fig. S6, D and E). If, in contrast, suppression was caused by the loss of gene-specific trafficking functions, increasing these functions should enhance polarity conversion. Consistent with this prediction, expression of the constitutively active RAB-7Q68L (*52*) increased the severity of polarity conversion in *let-767(RNAi)* intestines (Fig. 7D).

We conclude that RAB-6.2, ARF-6, RAB-7, DAB-1, and VHA-6 are required for the basolateral mislocalization of apical membrane components in GSL-depleted *C. elegans* intestines, likely affecting this process by their specific trafficking functions. These five GSL suppressors would therefore be expected to operate on either (i) anterograde basolateral trafficking routes downstream of GSL-directed apical sorting/routing sites (Fig. 7E, route 4) or (ii) routes upstream of these sites (Fig. 7E, routes 1 and 2). Disrupting these routes would reduce basolateral mislocalization of apical membrane components upon GSL depletion either directly (Fig. 7E, route 4) or indirectly, by reducing influx into the GSL-directed apical trajectory (Fig. 7E, routes 1 and 2; disruption at red X—route 3—is neutral or enhances misrouting, except if suppressor inhibits). These scenarios suggested that the suppressor analysis was unlikely to identify downstream effectors of the GSL-dependent secretory trajectory for apical membrane positioning (Fig. 7E, route 3), but might instead identify trafficking routes supplementing it (Fig. 7E, routes 1 and 2).

### The GSL suppressors DAB-1, RAB-7, and VHA-6 are required for the biogenesis, but not the positioning, of apical and basolateral membrane domains in wild type

To take advantage of the identified GSL suppressors for the characterization of trafficking routes supplementing the secretory pathway’s apical trajectory during de novo polarized membrane biogenesis, we next examined the three strongest suppressors’ effects on expanding membranes of wild-type larval intestinal cells. In the mature *C. elegans* intestine, DAB-1, RAB-7, and VHA-6 have documented roles in endocytosis, endo-to-lysosomal transport, and lumen acidification, respectively (*52*–*54*). Tier-1 screens had revealed these suppressors’ shared phenotype profiles in wild-type inTER- and inTRAcellular lumenogenesis, marked by defects in apical membrane biogenesis but not in apical domain positioning (Fig. 2, figs. S3 and S4, and table S1). Consistent with trafficking-based functions in de novo apical membrane biogenesis, confocal analysis of fluorescent fusions detected all three suppressors on endosomal vesicle and/or apical membranes in growing wild-type larval intestines. DAB-1 localization, thought to be restricted to basolateral membranes (*53*), was also found on expanding apical membranes and on two distinct vesicle populations in larval intestines (Fig. 7F and fig. S6F). VHA-6, thought to be restricted to apical membranes (*54*), was also found on subapical vesicles during membrane expansion (Fig. 7F). RAB-7, known to colocalize with early and late endosomal, lysosomal, and lysosome-related organelle (LRO) markers in adult intestines (*52*), had previously been observed to form subapical aggregates in L1-larval intestines (*8*). *dab-1*, *rab-7*, and *vha-6* RNAi also induced distinct vesicle biogenesis defects (Fig. 7G).

**Fig. 8.**
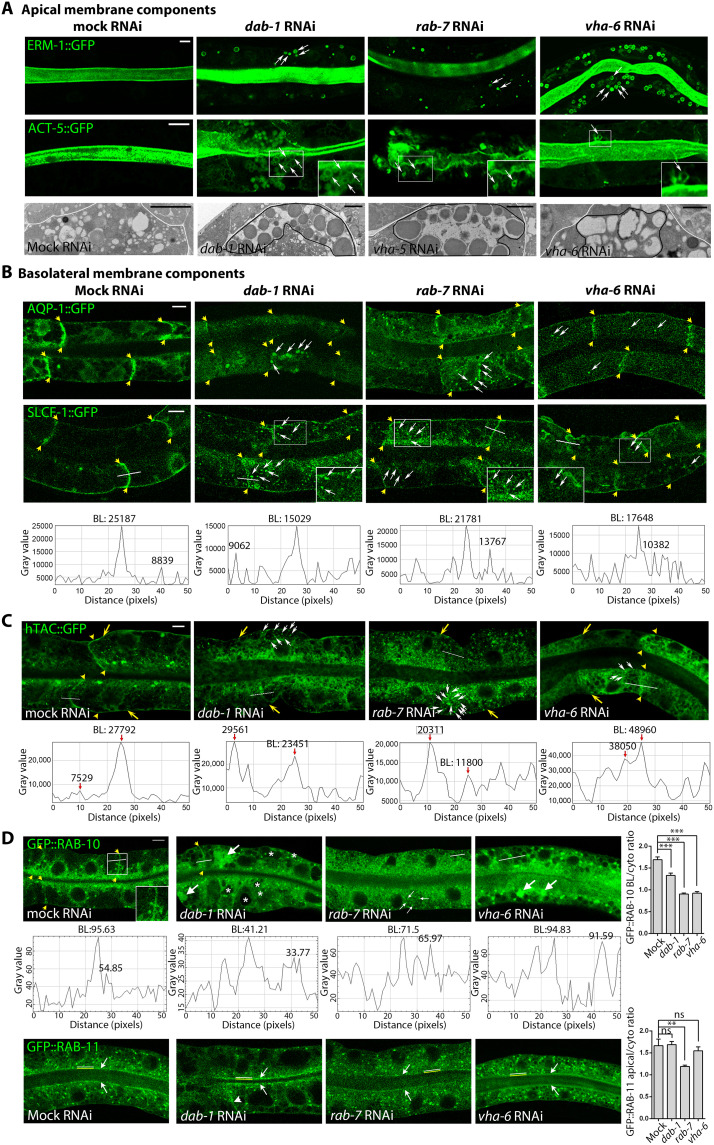
The GSL suppressors DAB-1, RAB-7, and VHA-6 are required for polarized membrane biogenesis, not positioning, in WT and function in recycling. (**A**) *dab-1*, *rab-7*, and *vha-6* RNAi displaces ERM-1 and ACT-5 (apical in WT/mock) to cytoplasmic vacuoles (arrows) during net polarized membrane addition. Here and below, thin confocal sections of two pairs of larval INT cells are shown. TEM images beneath: vacuolar aggregates, outlined in black (part of one larval INT cell is shown, boundary outlined in white). (**B**) AQP-1 and SLCF-1 (BL in WT/mock) are lost from *dab-1*, *rab-7*, and *vha-6(RNAi)* expanding BL membranes (lateral portions bracketed by yellow arrows) and displaced to small cytoplasmic vesicles or tubulovesicles (white arrows). Fluorescence intensity across SLFC-1^+^ membranes (white line, length: 50 pixels) is shown beneath, with highest cytoplasmic intensity point indicated. (**C**) hTAC (BL in WT/mock) is lost from expanding *dab-1*, *rab-7*, and *vha-6(RNAi)* BL membranes (yellow arrows; arrowheads bracket lateral portions) and displaced to cytoplasmic vesicles (white arrows). Fluorescence intensity across hTAC^+^ membranes (white line, length: 50 pixels) is shown beneath. (**D**) RAB-10^+^ vesicles are displaced from BL membranes (yellow arrows) and retained in cytoplasmic vesicle aggregates, clusters of small vesicles, and enlarged vesicles (white arrows indicate examples of each) in larval *dab-1*, *rab-7*, and *vha-6(RNAi)* INT cells, respectively. Asterisks: *dab-1(RNAi)*–specific vacuoles that can contain RAB-10^+^ material. Fluorescence intensity across RAB-10–enriched BL membranes (white line, length: 50 pixels) shown beneath with highest cytoplasmic intensity point indicated. In contrast, RAB-11^+^ vesicles largely maintain their WT apical enrichment [white arrows; note some loss in *dab-1* and *rab-7*, and occasional BL displacement in *dab-1(RNAi)* INT cells (arrowhead)]. Bar graphs: quantification of the fluorescence intensity ratio of membrane-associated to cytoplasmic vesicles. Mean ± SEM is shown (*n* = 3/*N* > 8). Two-tailed Student’s *t* test was used to calculate *P* values. Scale bars, 5 μm and 1 μm in TEM images.

To confirm that the three GSL suppressors affected apical membrane assembly (acquisition of apical membrane character: ERM-1 recruitment from the cytoplasm; Fig. 1B/phenotype class ③: cytoplasmic ERM-1 displacement with ERM-1^+^ vacuoles) rather than apical membrane positioning (regulation of membrane polarity: apicobasolateral ERM-1 distribution along the membrane; Fig. 1B/phenotype class ④) in wild-type intestines, we reassessed net polarized membrane addition in *dab-1*, *rab-7*, and *vha-6(RNAi)* expanding postmitotic cells at their fixed positions in growing larval intestines. ERM-1 and ACT-5/actin were found to be increasingly retained on ERM-1^+^ and ACT-5^+^ cytoplasmic vacuolar inclusions but not misdirected to basolateral domains during de novo apical membrane biogenesis (Fig. 8A). By confocal microscopy, apical membrane–bounded vacuolar inclusions are indistinguishable from ectopic lumens, a feature of polarity conversion when located at basolateral membranes. To scan for the presence/absence of ectopic lumens in *dab-1*, *vha-6*, and *vha-5(RNAi)* larval intestinal cells, we used serial transmission electron microscopy (TEM) sectioning (Fig. 8A and fig. S7, A and B; *vha-5* RNAi also targets its paralog *vha-6*; see fig. S7A legend). This analysis failed to detect vacuoles with inward-pointing microvilli, suggesting that the observed vacuolar inclusions were endomembrane intermediates en route to or from the apical membrane and not ectopic lumens indicative of apicobasal polarity conversion. These results were consistent with a function of GSL suppressors in wild-type apical membrane assembly but not positioning.

Basolateral membrane expansion was monitored under the same conditions to determine whether the suppressors’ function in wild-type polarized membrane biogenesis was limited to the apical membrane. Unexpectedly, *dab-1*, *rab-7*, and *vha-6* RNAi also displaced the sugar transporter SLCF-1 and the water channel AQP-1/aquaporin from expanding basolateral membranes, retaining them on small vesicles/tubulovesicles, but did not misdirect these integral membrane components to the opposing (apical) domain in wild-type larval intestinal cells (Fig. 8B; see fig. S7C for quantification).

We conclude that DAB-1, RAB-7, and VHA-6, each necessary for apicobasal polarity conversion in GSL-depleted *C. elegans* intestines, are required for both apical and basolateral membrane biogenesis but not positioning in wild-type intestines, and hence appear dispensable for determining wild-type apicobasal membrane polarity. The dual roles of DAB-1, RAB-7, and VHA-6 in apical and basolateral membrane biogenesis suggested that these three GSL suppressors interact with GSLs in polarity from a position upstream of a GSL-dependent apical vesicle trajectory, operating either on (i) secretory routes, moving unsorted apicobasal cargo toward GSL-dependent sorting/routing sites, as part of, or parallel to, the GSL-directed trajectory (Fig. 7E, route 1) or (ii) interfacing endocytic-recycling routes, moving both apical and basolateral cargo toward these sites (Fig. 7E, route 2).

### The suppressors of apicobasal polarity conversion uncover polarized recycling routes that replenish secretory routes to manufacture new polarized membrane

To distinguish whether DAB-1, RAB-7, or VHA-6 supplemented the GSL-dependent vesicle trajectory for apical domain positioning on secretory or recycling routes, we first asked if these three molecules operated in secretion or recycling at the time of de novo polarized membrane biogenesis and monitored basolateral trafficking routes, well characterized in mature intestines (*55*), during net membrane addition in larval intestinal cells. We found that *dab-1*, *rab-7*, and *vha-6* RNAi (i) retained the recycling marker hTAC::GFP on cytoplasmic vesicles in larval intestinal cells (Fig. 8C; see fig. S7D for quantification); (ii) failed to accumulate Yolk, a marker for secretion, in these cells (fig. S8A; see legend for Yolk assay); and (iii) did not abort membrane delivery of TGN-38(Y314A), a Golgi resident trapped at the membrane due to a recycling defect (fig. S8B) (*56*). We concluded that the three GSL suppressors operate in recycling, not secretion, during de novo membrane biogenesis, suggesting that they supplement the GSL-dependent secretory trajectory for apical domain positioning on polarized recycling routes (Fig. 7E, route 2).

**Fig. 9.**
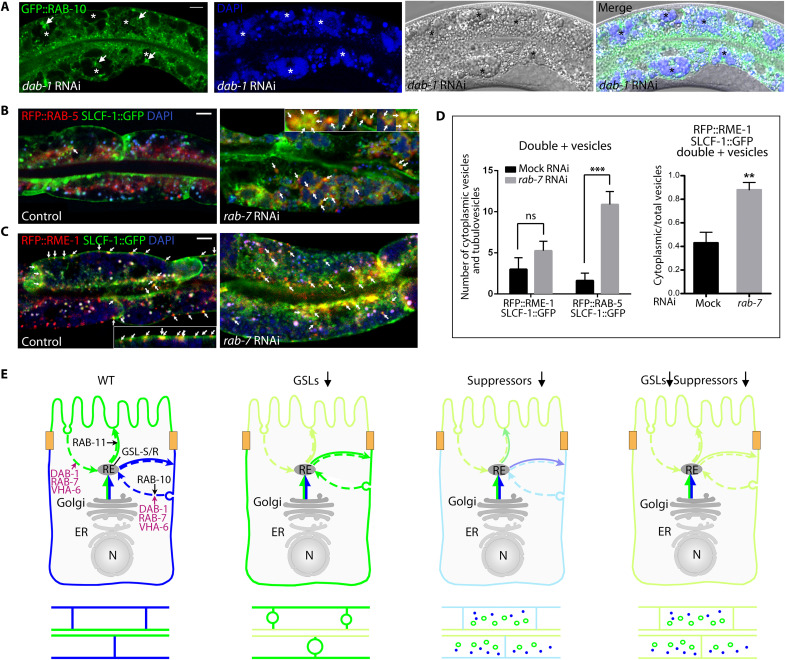
The suppressors of the GSL-dependent polarity conversion supplement de novo polarized membrane biogenesis in WT via polarized recycling. (**A**) GFP::RAB-10^+^ material (arrows) accumulates in *dab-1(RNAi*)–specific DAPI^+^ vacuoles (asterisks) during membrane expansion in larval INT cells. (**B** to **D**) The integral membrane component SLCF-1 traverses endocytic-recycling carriers during de novo polarized membrane biogenesis, a process requiring RAB-7. (B) and (C) Left: occasional SLCF-1 passage through RAB-5^+^ membrane-derived, and RME-1^+^ membrane-directed, arms of endosomal-recycling routes (arrows) in WT (control). Note close association of RFP::RME-1^+^; SLCF-1::GFP^+^ vesicles with membrane-attached tubules [inset in (C)]. Right: *rab-7* RNAi increases cytoplasmic SLCF-1::GFP^+^; RFP::RAB-5^+^ [inset in (B)], but not SLCF-1::GFP^+^; RFP::RME-1^+^ vesicles, although it broadens membrane-associated SLCF-1::GFP^+^; RFP::RME-1^+^ tubulovesicles during membrane expansion. Note loss of BL SLCF-1 and enlarged LROs (blue), demonstrating *rab-7(RNAi)* effect. (D) Quantification of SLCF-1::GFP^+^; RFP::RME-1^+^ and SLCF-1::GFP^+^; RFP::RAB-5^+^ cytoplasmic vesicles/tubulovesicles, and ratio of SLCF-1::GFP^+^; RFP::RME-1^+^ cytoplasmic to total (cytoplasmic plus membranous) vesicles/tubulovesicles. Mean ± SEM is shown (*n* = 3/*N* = 8). Analysis of variance (ANOVA) was used to determine statistical significance. Confocal sections or Nomarski/Nomarski-confocal overlay images of one to two pairs of larval INT cells are shown. Scale bars, 5 μm. (**E**) Model for suppression. Reduction of flux from polarized endocytic-recycling routes into the GSL-dependent apical secretory route reduces apical-to-BL misrouting upon GSL depletion and suppresses polarity conversion. WT and single and double GSL/suppressor depletion conditions are shown, single cells on top, corresponding tissue phenotypes beneath. The direction of the delivery of polarized membrane components (arrows) and the putative location of the suppressors (DAB-1, RAB-7, VHA-6) on corresponding delivery routes are indicated. RAB-10 and RAB-11 are shown as markers for BL and apical recycling carriers that could be engaged by the suppressors. GSL-S/R is arbitrarily placed at the RE. Secretory routes: solid lines, recycling routes: dashed lines. Reduced cargo flux to (or arrival at) plasma membranes is indicated by lighter colors. N, nucleus.

We next asked if DAB-1, RAB-7, or VHA-6 could spatially direct polarized vesicles on recycling routes during net membrane addition and tracked the positions of RAB-11^+^ and RAB-10^+^ recycling endosomes in growing larval cells (Figs. 8D and 9A). RAB-11 and RAB-10 mark two of few vesicle populations with documented (albeit not exclusive) functions on apical (predominantly anterograde) and basolateral (predominantly retrograde) recycling routes, respectively (*55*). Tier-2 interaction screens had identified RAB-11 as a loss-of-function enhancer of the GSL-dependent polarity conversion (Fig. 3C), suggesting that RAB-11 acts in parallel to, or downstream of, GSLs on an anterograde apical vesicle trajectory that might pass through the recycling compartment (Fig. 7A, route 2; Fig. 7E, route 3). If so, the three suppressors, expected to promote recycling upstream of GSLs, should function via RAB-10^+^ but not RAB-11^+^ recycling carriers during net membrane addition. In *dab-1*, *rab-7*, and *vha-6(RNAi)* larval intestinal cells, GFP::RAB-11^+^ vesicles were largely retained at expanding apical membranes, while GFP::RAB-10^+^ vesicles aggregated in the cytoplasm and were lost from basolateral membranes (Figs. 8D and 9A). These results were consistent with a function of DAB-1, RAB-7, and VHA-6 in the routing of vesicles on retrograde arms of polarized recycling routes that operate during de novo polarized membrane biogenesis.

Collectively, these findings suggested a scenario where the GSL suppressors DAB-1, RAB-7, and VHA-6 direct endocytic-recycling carriers returning previously polarized membrane components to GSL-dependent secretory routes that position the apical domain during de novo polarized membrane biogenesis. Recycling of polarized cargo is thought to increase the fidelity of cargo sorting rather than to supplement the de novo biogenesis of polarized membrane (*5*). However, the delivery of polarized membrane components to or from the membrane during net membrane addition has not yet been visually tracked. We used double-labeling of polarized cargo-carrier pairs to track this process in *C. elegans* larval intestinal cells. The above-outlined scenario would predict that, during de novo membrane biogenesis, polarized integral membrane components traverse endocytic-recycling carriers in which they become trapped in the absence of DAB-1, RAB-7, or VHA-6. We found that the integral membrane component SLCF-1 could be detected in RAB-5^+^ (presumed membrane-derived) and RME-1^+^ (presumed membrane-directed) endocytic-recycling carriers (*55*) during net polarized membrane addition (Fig. 9, B to D, and fig. S8, C and D). Moreover, depleting RAB-7 increased RFP::RAB-5; SLCF-1::GFP but not RFP::RME-1; SLCF-1::GFP double-positive vesicles in larval intestinal cells, indicating that RAB-7 depletion trapped SLCF-1 on endocytic arms of polarized recycling routes during polarized membrane expansion (Fig. 9, B to D, and fig. S8, C and D). RAB-7 depletion also increased the cytoplasm to plasma membrane–associated ratio of RFP::RME-1; SLCF-1::GFP double-positive vesicles, consistent with SLCF-1’s slowed progression through the recycling compartment. This finding suggested that RAB-7’s well-characterized function downstream of RAB-5 may not be limited to vesicle degradative routes during de novo polarized membrane biogenesis, but at this time include membrane-directed recycling routes [Fig. 9, B to D; such a function might, for instance, be independent of the RAB-7 guanine nucleotide exchange factor (GEF) SAND-1 whose loss had failed to suppress polarity conversion; fig. S6C].

In summary, the analysis of GSL suppressors identifies polarized recycling routes that promote the biogenesis, but not the positioning, of apicobasal membrane domains in wild-type *C. elegans* intestines, with previously unknown functions on such routes for DAB-1, RAB-7, VHA-6, and four additional candidate components of these recycling routes (ARF-6, RAB-6.2, DPY-23, and ARL-8). DAB-1, RAB-7, and VHA-6, although themselves dispensable for apical domain positioning, support GSLs in this polarity function via their canonical roles in endocytic recycling that here serve to supply the GSL-directed secretory route that positions the nascent apical domain. Reducing influx into the GSL-directed apical vesicle trajectory reduces basolateral misrouting of apical membrane components upon GSL depletion and hence suppresses the GSL-dependent apicobasal polarity conversion (Fig. 9E; see fig. S8E for possible conclusions regarding the itinerary of a GSL-directed secretory route for apical domain positioning). We conclude that polarized membrane components are directly recycled back to manufacture new polarized membrane in the expanding *C. elegans* intestine, revealing polarized recycling as an integral part of de novo polarized membrane biogenesis.

## DISCUSSION

**Fig. 10.**
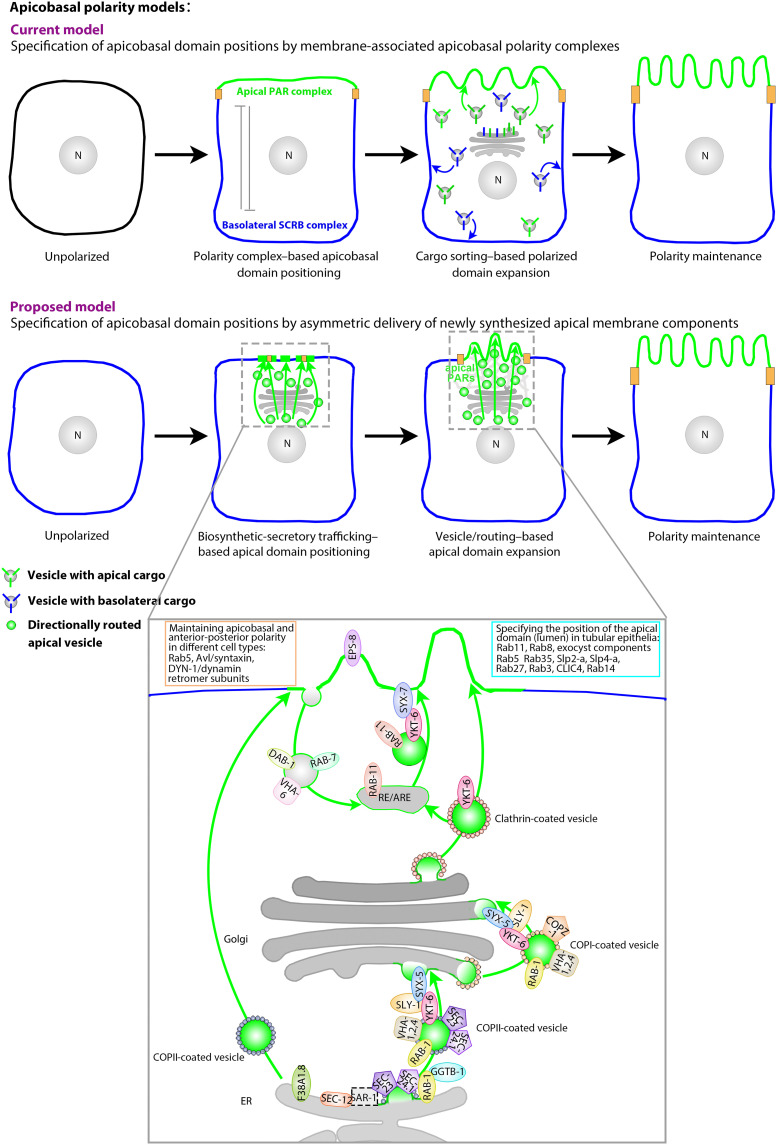
Current and proposed epithelial polarity models. Membrane polarization is shown as proceeding from left to right, cell 1 to cell 4. Current model (first row): Apicobasal membrane domains are first demarcated at the cortex by the interdependent cortical alignment of membrane-/junction-based apical and basolateral polarity complexes (only apical PARs and BL SCRIB complexes are indicated; cell 2). Sorting of apicobasal membrane components (cargo) to cognate recognition sites at previously polarized membrane domains expands and maintains the polarized domains (cell 3). Proposed model (second row): The secretory pathway is asymmetrically routed to the membrane by a dynamic cytoskeleton [gray mesh; cell 3; see our accompanying article (*51*)] and concomitantly positions, assembles, and expands the nascent apical domain into the growing membrane. Apical PARs and junctions are subsequently (or concomitantly) polarized. They could be directly delivered by vesicles or indirectly recruited to the newly synthesized apical domain. Junctions subsequently assemble at the apico-lateral membrane angle, securing the expanding apical domain. N: nucleus; gray structure above: Golgi; mesh above: proposed vectorial cytoskeleton; colors as in fig. S1. Not all components of the system are indicated in all cells. Magnification below: a unifying trafficking model for a single secretory vesicle trajectory traversing the recycling compartment on its way to the nascent apical domain (accommodating all identified vesicle-based polarity cues). Possible Golgi bypass routes [recently also documented in the *C. elegans* INT (*110*)] and possible direct routes from the Golgi to the apical domain are also indicated. COPI, COPII, and clathrin vesicle coats (shown as vesicle “ears”) could provide the physical connection to the guiding cytoskeleton (not shown here). RE/ARE: recycling/apical recycling endosome. Box at left upper corner: molecules required to maintain apicobasal and anterior-posterior polarity (*15*–*19*); box at right upper corner: molecules required for the positioning of the apical domain (lumen) in tubular epithelia (*20*–*27*, *34*).

Here, we identify the secretory pathway, supplemented by polarized recycling routes, as key determinant of apicobasal membrane polarity in the *C. elegans* intestine. Our analysis indicates that the directionality of this essential trafficking pathway is regulated upstream of PARs and independent of polarized membrane domains to asymmetrically insert the apical domain into the growing epithelial membrane and thereby determine this membrane’s apicobasal polarity. We isolate multiple components of secretory carriers located throughout the pre- and post-Golgi endomembrane system that function as directional cues for the polarized distribution of apical membrane and PAR complex components on not-yet polarized membranes during polarity establishment and on already polarized membranes, whose established polarity they can change as long as new membrane is being synthesized. Together, our findings suggest that, during de novo polarized membrane biogenesis, the anterograde secretory pathway, required to supply all sides of the plasma membrane, becomes routed to a nascent apical domain that it concomitantly assembles, positions, and expands. This alternative mode of membrane polarization shares features with early polarity models that envisioned the predetermined (apical or basolateral) directionality of bulk membrane delivery to polarize the epithelial membrane (*9*, *10*). Such an alternative mode of determining membrane polarity could also address questions of current models of polarity and polarized trafficking (Fig. 10; Introduction and below).

An upstream polarity function of intracellular vesicular trafficking, regulated to assume apical directionality but not intrinsically polarized, resolves the current polarity model’s difficulty that the canonical, membrane-based polarity cues (e.g., the PARs) and junctions must themselves be polarized to membrane domains whose positions they ought to determine. The well-characterized signaling network of mutual inhibition between these canonical, apical, and basolateral membrane-based polarity cues (*1*, *2*, *4*), along with the assembly of apical junctions, is, conversely, well placed to secure the boundaries of a nascent apical domain that is expanded into the growing membrane by the secretory pathway, as suggested here (Fig. 10). The proposition that lateral junctions are only secondarily assembled is supported by the initial appearance of spot junctions at the nascent apical membrane of polarizing *C. elegans* intestinal cells (*47*) and by the de novo assembly of junctions around ectopic basolateral lumens during apicobasal polarity conversion (*8*, *12*). The here proposed alternative polarity model does not preclude the concomitant or subsequent interdependent cortical alignment of the membrane-based apical and basolateral polarity cues, nor of endomembrane-based apicobasal cargo sorting (once membrane domains are polarized). Intracellular (trafficking-based) and membrane-based polarity cues might in fact mutually reinforce each other during the process of membrane polarization, as, for instance, suggested by effects of apical PARs on polarized trafficking (*57*).

A flexible directionality of the secretory pathway, regulated independent of cargo sorting, also addresses difficulties of sorting-based models of polarized trafficking that place the directional control of trafficking with polarized cargo signals and their carrier-based decoders (*1*, *5*). For example, cargo- or carrier-based signals fail to consistently sort in one direction (e.g., clathrin/AP-1 sort to both apical and basolateral domains, a problem unresolved by cell type–specific adaptors such as AP-1B) (*58*–*61*). It is also still unclear how sorting confers long-range apicobasal directionality to vesicles and whether and where these vesicles travel: (i) randomly (e.g., captured only at their polarized target domain by cognate v-/t-SNARE or coincident phosphoinositide pairs) (*62*, *63*), (ii) guided (e.g., by cytoskeletal tracks/motors) (*64*, *65*), or (iii) by endomembrane maturation (e.g., via Rab cascades) (*66*). Together with the evidence presented in our accompanying article (*51*), our findings suggest that cytoskeletal, spatially, and temporally regulated guidance cues determine trafficking’s directionality during de novo polarized membrane biogenesis in epithelia.

There is precedence for a regulated change of the secretory pathway’s directionality in nonepithelial cells (*67*–*69*). In budding yeast, this pathway is actin-dependently oriented to the bud site during polarized bud growth to direct a full set of nonpolarized membrane components to the daughter cell (*68*). In epithelial cells, however, the directional routing of polarized (e.g., apical) membrane components—proteins and lipids (e.g., GSLs)—will asymmetrically expand and thereby polarize the membrane. Membrane lipid measurements in polarized versus nonpolarized Madin-Darby canine kidney (MDCK) cells reveal the net addition of apical lipids to the resident complement of membrane lipids (*70*). Therefore, a transient increase in apical cargo/carrier components (e.g., via an increase in GSL biosynthesis) could regulate and also initiate membrane polarization. Consistent with this proposition, the transcriptionally regulated biosynthesis of sterols (raft components, like GSLs) (*5*, *6*) was shown to initiate polarity and operate upstream of canonical polarity cues in fission yeast (*71*).

Membrane-based landmarks other than already polarized membrane domains could orient trafficking routes directly or indirectly via branched-chain actin dynamics that direct secretory vesicles to the nascent apical domain in polarizing *C. elegans* intestinal cells [see our accompanying article (*51*)]. The midbody, for instance, serves as a landmark in the trafficking-dependent process of apical domain positioning in MDCK cysts (*72*). During polarity establishment in the *C. elegans* intestine, the midbody moves to (and then remains at) the apical domain and associates with polarized RAB-11^+^ vesicles before PARs are polarized (*73*). Developmentally regulated transcriptional signals alone could initiate the apical reorientation of trafficking, either directly (e.g., via GSLs, as above) or via branched-chain actin dynamics. A transcriptional increase of branched-chain actin modulators was, for instance, recently shown to establish polarity in mouse oocytes (*74*). Basement membrane–associated extracellular matrix signals, although so far elusive in the *C. elegans* intestine (*46*, *49*), could, in principle, organize the actin cytoskeleton (as, for instance, in the *Drosophila* follicular epithelium) (*75*–*77*) or directly polarize trafficking routes from the basolateral membrane, as demonstrated in MDCK cysts (*78*). Finally, lateral membrane–based PAR-3 and HMR-1/cadherin complexes were recently proposed as intercellular polarity triggers in the *C. elegans* intestine; however, they must, like PARs and junctions, themselves be polarized (*79*).

A polarity model based on the directional insertion of apical membrane components into the growing membrane can also serve as a morphogenetic model that consolidates inTERcellular and inTRAcellular apical domain and lumen biogenesis (fig. S9). Notably, inTRAcellular lumenogenesis models propose the expansion and polarization of the apical/lumenal membrane by directional vesicle coalescence (*57*, *80*, *81*). If such a process of net apical domain extension within a single cell (e.g., inside the junction-less unicellular *C. elegans* canal) proceeds similarly between pairs of cells (e.g., in the multicellular *C. elegans* intestine), it will position the apical, and consequently the basolateral, domain (fig. S9). Consistent with this idea, all trafficking components that we found to regulate polarity in the intestine also direct apical membrane expansion in the canal (the only exception is EPS-8, with a likely intestine-specific and possibly actin-dependent polarity function). In support of this observation, we note that several trafficking molecules have been identified by a function in the polarity of multicellular tubular epithelia that were independently identified by a function in the expansion of unicellular tubular epithelia [e.g., Rab35, Rab5, exocyst components, Slp/BTZ (bitesize), CLIC4/EXC-4] (*15*, *20*–*23*, *81*–*87*).

Secretory routes can intersect with recycling routes on their way to the apical membrane (Fig. 7A) (*88*, *89*). A secretory vesicle trajectory, routed from the ER to the nascent apical domain during membrane polarization, could therefore also accommodate apical endocytic and transcytotic recycling routes. Components of such routes, more amenable to loss-of-function studies than the essential secretory pathway, continue to be identified by their roles in apicobasal and anterior-posterior polarity (itemized in Fig. 10). All components of secretory or endocytic carriers identified by their ability to position the apical domain could thus regulate the directionality of a single common apical vesicle trajectory, e.g., by connecting it to a dynamic cytoskeleton that directs this trajectory to the apical plasma membrane during de novo membrane biogenesis [Fig. 10; see our accompanying article (*51*)]. Directing such a trajectory from the ER to the apical membrane might require components of (i) pre-Golgi secretory carriers (this study), (ii) Golgi/post-Golgi endomembranes/carriers (GSLs, clathrin/AP-1; prior studies by us and others) (*8*, *11*, *12*, *60*–*61*, *90*), and (iii) apical recycling carriers and the apical vesicle/membrane interface [e.g., RAB-11 (*20*), V-ATPases (*39*), and EPS-8 (*42*); this study and other studies; Fig. 10]. The idea of one common trajectory is supported by the close genetic interactions here observed between all three groups of vesicle-based polarity cues (pre-Golgi, post-Golgi/sorting, post-Golgi/recycling), where the combined depletion of any two components, mild enough to maintain polarity when only one is depleted, suffices to synthetically induce apicobasal polarity conversion.

It has been noted that secretory pathway components are conspicuously absent from the collection of trafficking molecules identified in numerous genetic screens on apicobasal polarity (*19*). This may be due, in part, to the narrow range of interference conditions that permit the separation of the pathway’s directional from its general secretory function. In contrast, screens on apical secretion and apical membrane expansion—processes more robust to pathway disruption—in both flat and tubular, especially in unicellular tubular, epithelia have repeatedly identified early pathway components (e.g., COPI/II subunits) (*25*, *91*–*96*). However, tracking cargo delivery beyond the membrane (assessing secretion) must miss, while tracking cargo accumulation in the cytoplasm (assessing membrane delivery) can mask, changes in the position (apical versus basolateral) of cargo at the membrane, which would explain these screens’ failure to identify the pathway’s regulatory function in apicobasal polarity. Secretory molecules were also identified in screens tracking the delivery of single apical cargo, carrier, or junction components (table S2), an approach likewise unable to identify the pathway’s function in determining membrane polarity. However, these screens provide ample confirmation for the secretory pathway’s direct involvement in apical cargo delivery that was here largely deduced from the recruitment of the apical identity marker ERM-1 (incidentally, polarity defects were occasionally observed in these screens but set aside or dismissed as nonspecific).

While anterograde secretory routes can intersect with recycling routes on their way to the membrane, retrograde recycling routes can intersect with pre- and post-Golgi secretory routes, an intersection thought to, respectively, replenish pre-Golgi intermediates and increase the fidelity of cargo sorting (*97*). Here, we identify endocytic-recycling routes that redirect polarized membrane components to the secretory pathway during net membrane expansion, suggesting that these membrane components can be immediately reused to manufacture new polarized membrane. This unexpectedly close interface of polarized secretion and recycling during de novo membrane biogenesis could explain the astonishing fluidity of membrane polarity that we observe in postmitotic cells within the fixed tissue context of the expanding *C. elegans* intestine where lumen positions can shift in situ between apical and basolateral sides. Such a dynamic aspect of single-cell polarity may be conserved through phylogeny, as indicated by the conservation—to the molecular level—of trafficking-dependent apicobasal polarity phenotypes from worms to human intestinal disease phenotypes [e.g., microvillus inclusion disease (*98*)]. Apical-to-basolateral polarity conversion in the *C. elegans* intestine mirrors basolateral-to-apical polarity inversion in mammalian MDCK cysts, where the transition to 3D culture conditions shifts the apical domain from the future basolateral to its new apical location (fig. S10) (*20*). The two distinct in vivo (*C. elegans*) and in vitro (MDCK cells) models may thus provide complementary vistas on the same trafficking-directed process of membrane polarization, with apical recycling molecules such as RAB-11/Rab11 (identified in both models by their apicobasal conversion/inversion phenotypes) marking shared components of one common apical secretory trajectory that traverses the recycling compartment. A developmentally regulated, directionally flexible biosynthetic-secretory pathway, in close apposition to polarized recycling routes that permit exchange between all membrane domains, would provide greater plasticity of single-cell polarity than currently appreciated.

## MATERIALS AND METHODS

### Experimental model and subject details

An introduction to the in vivo analysis of polarized membrane biogenesis in the *C. elegans* intestine and excretory canal, with an extended Methods section and a visual demonstration of the techniques used in this study, is provided in (*99*, *100*).

### *C. elegans* strains, culture conditions, and genetics

Wild-type (N2 Bristol) and mutant *C. elegans* strains were cultured, and genetic crosses were performed using standard methods (*101*). Worms were generally maintained at 20° to 22°C on Nematode Growth Medium (NGM) plates seeded with *Escherichia coli* *(E. coli)* OP50 (*102*). The list of strains used in this study is provided in table S4.

### RNAi/loss-of-function analysis

RNAi was carried out by feeding worms *E. coli* HT115 (DE3), producing double-stranded RNA (dsRNA) of the gene of interest, as previously described (*8*, *103*). For standard RNAi, bacterial feeding clones were inoculated from LB plates into 1 ml of LB liquid medium containing ampicillin (50 μg/ml) and incubated for 8 to 18 hours at 37°C. Cultured RNAi bacteria (200 μl) were seeded onto agar plates supplemented with 2 mM isopropyl-β-d-thiogalactopyranoside (IPTG) and carbenicillin (25 μg/ml). dsRNA was induced at room temperature for at least 6 hours before picking four to six L4 larvae onto each RNAi plate. Most bacterial clones were derived from the Ahringer genome-wide RNAi feeding library (J. Ahringer, Welcome Trust/Cancer Research UK Gurdon Institute, Cambridge, UK). The integrity of all RNAi clones was verified by sequencing (discrepancies to reported sequence information are noted in table S1).

RNAi was the method of choice for the here performed systems-based loss-of-function analysis of essential genes (most genes are sterile, maternal-effect and early lethal): It (i) produces a broad range of phenotypes, (ii) can be titrated to induce very mild to severe effects, and (iii) effectively targets maternal RNA (germline mutants provide maternal product via the balancer). In tier-1 screens, moderately severe (standard) RNAi conditions were used to generate a balanced spectrum of phenotypes. To avoid full disruption of membrane-directed trafficking, a scaled-intensity RNAi approach was used to analyze the secretory pathway [see our accompanying article (*51*)]. Mild to moderately severe RNAi conditions were empirically determined for any given gene by modulating IPTG concentrations, diluting the RNAi clone of interest with mock bacteria (empty vector), varying the developmental stage (L2 to adult) of the parental strain in which RNAi was induced, and using the RNAi-sensitive strain *rrf-3(pk1426)* (*99*, *100*). To achieve interference at different time points during embryonic and/or later stages of development, RNAi was induced: in parents (evaluating the F1 progeny; standard parental RNAi), in larvae (evaluating the same generation; conditional larval RNAi), or in adults. To start RNAi at the L1-larval stage, conditional RNAi was carried out by bleaching 30 to 50 gravid adults in one drop bleaching solution (a 1:4 mix of 10 M NaOH and household sodium hypochlorite) on the edge of an RNAi plate and allowing hatched larvae to crawl to the bacterial lawn [see (*100*) for details]. Appropriate controls were added to ensure that RNAi was effective when induced at later time points during development or in adults [e.g., by using *gfp* RNAi on a green fluorescent protein (GFP)–expressing strain].

### Tier-1 tubulogenesis screens and selection steps

Screens I and II, genome-wide RNAi-based intestinal and excretory canal tubulogenesis screens (Fig. 1B) (*8*, *12*), first examined the tubulogenesis phenotype of each chromosome III gene knockdown in a pilot screen. This pilot screen revealed that >90% of genes whose losses induced informative tubulogenesis phenotypes in ERM-1::GFP-labeled intestinal and excretory canal epithelia were lethal. Screens were therefore restricted to all lethal genes in the genome (*N* = 4978), with the expectation of achieving >90% genome coverage for identifying genes required for tubulogenesis in this experimental setting. Intestinal and excretory canal tubulogenesis was examined in live animals throughout development, first under a dissecting fluorescent microscope, subsequently by confocal microscopy. More than 20% of genes, selected by phenotypic characteristics promising to be better defined at higher resolution, were examined by confocal microscopy. Emphasis was placed on the analysis of embryonic and early larval development, the time of intestinal and canal morphogenesis. Screen III, a targeted RNAi-based screen aimed at identifying enhancers and suppressors of the ERM-1[++] cystic short canal phenotype (Fig. 1B) (*28*), examined broadly extended classes of genes predicted or experimentally demonstrated to interact with ERM-1/ezrin-radixin-moesin in any species (*N* = 1846). Modification of the ERM-1[++] excretory canal defect (canal re-extension versus further canal shortening and cystic enlargement) was assessed under a dissecting fluorescent microscope in live animals carrying an integrated transgene, providing additional *erm-1* copies (*fgIs2[perm-1::erm-1]*) and an extrachromosomal transgene labeling the canal cytoplasm (the transcriptional fusion *psulp-5*::GFP).

Subsequent selection steps used in this study (fig. S2) were carried out as follows: (i) From all tubulogenesis phenotypes identified in screens I to III, those phenotypes were selected that had defects in “apical/lumenal membrane assembly, positioning or expansion” (as defined by type of ERM-1 displacement, described in the text); (ii) after unblinding the identities of all genes whose losses caused such defects in apical membrane biogenesis, those genes were selected that had been implicated in vesicular trafficking. All genes were selected whose direct or homologous gene products had reported functions in vesicular trafficking in *C. elegans* or other species, based on published experimental data and search engines [e.g., AceView (http://www.ncbi.nlm.nih.gov/ieb/research/acembly/), Ihop (http://www.ihop-net.org/UniPub/iHOP/), the Yeast genome database (http://www.yeastgenome.org), Flybase (http://www.flybase.org), the Mouse Genome Database (http://www.informatics.jax.org), and the Human Genome Database (http://www.gdb.org)]. Fifty trafficking genes were identified. All 50 gene products were highly conserved from yeast to human. Gene products were classified according to structure and trafficking function and annotated with regard to their yeast and human orthologs, cellular and subcellular localization, and, where available, trafficking route, with specific attention to functions described in *C. elegans* (collected in table S1).

### Phenotype analysis

Phenotypes were evaluated in multiple independent experiments (*n* > 5, with *N* > 500 animals per plate), performed in triplicates. Animals were characterized alongside a positive control (e.g., *let-767* RNAi for the larval intestinal polarity phenotype and *chc-1* RNAi for the embryonic intestinal polarity phenotype) and a negative control [empty vector or vector with unrelated gene, typically a larval or embryonic lethal gene (itemized in table S1)]. Note that all negative results of the initial tier-1 screens (>5000 genes; Fig. 1B) serve as additional negative controls. Interference with most of the essential genes induced moderate-to-severe body morphogenesis defects. The identification of specific phenotypes within this background (e.g., phenotype classes ① to ➉; Fig. 1B) was made possible by the membrane-level, single-cell resolution of the visual analysis (tracking of fluorescently labeled apical membranes in the single-layered intestinal and single-cell canal epithelium). RNAi phenotypes of representative pathway genes were further confirmed via phenocopy by germline mutants or RNAi with additional subunit or pathway components of the targeted gene, dose dependency of the phenotype, and/or independent studies (as described in the text). Cell-autonomous functions of selected genes were confirmed by phenocopy in an intestine-specific RNAi strain (*rde-1* mutant background supplemented with an intestine-directed *rde-1* transgene) (*99*, *100*).

For the standard analysis of RNAi phenotypes, the progeny of two RNAi plates, seeded with four L4 stage *erm-1p::erm-1::gfp; rol-6p::rol-6(su1006)* larvae, was evaluated. The dominant marker *rol-6*, conferring a circumferential rolling movement, facilitates the 3D (apicobasolateral) evaluation of polarized tubulogenesis. RNAi conditions were empirically determined and varied to (i) track polarized membrane biogenesis throughout the entire postmitotic growth phase (see fig. S1) and (ii) examine a spectrum of mild-to-severe loss-of-function phenotypes. All animals were evaluated throughout development by high-power dissecting and/or confocal microscopy, with most phenotypes present in embryos and/or early larvae. For the initial assessment of lethality (table S1), the F1 progeny was counted on day 3. The morphology of embryos was assessed by differential interference contrast (DIC) (Nomarski) microscopy (see below, microscopy). Sterility was scored based on the number of total F1 progeny per plate, and embryonic morphology defects/larval arrests were counted per 100 embryos/larvae, respectively.

In the repeat RNAi analysis of the 50 identified trafficking genes, all RNAi clones were examined in two rounds: one focusing on embryonic, the other on early larval development. Intestinal and canal apical/lumenal membrane biogenesis was assessed in parallel in the same animal. Absence/presence of specific phenotype subclasses (e.g., basolateral or vacuolar ERM-1::GFP displacement) was examined in separate sets of experiments using empirically determined gene-specific RNAi conditions, as described above.

For the extended RNAi analysis of apical membrane assembly, positioning, and expansion defects, embryonic and larval intestinal and excretory canal tubulogenesis and ERM-1::GFP placement were evaluated 4 to 6 hours over day during the window of ~48 hours required for full tube extension (mostly longer, given gene-specific growth delay), throughout the process of net polarized membrane expansion (see fig. S1). Phenotypes that occurred later (≥72 hours) were not included, given the high percentage of nonspecific late effects caused by membrane biogenesis defects. For example, intestinal membrane polarity defects (basolateral ERM-1::GFP mislocalization) can be obscured during polarized membrane expansion by increasing cytoplasmic ERM-1::GFP displacement, the development of structural apical/lumenal membrane defects (e.g., lumen widening), vacuolar ERM-1::GFP displacement, and other tubulogenesis defects.

### Tier-2 genetic interaction screens

*let-767(s2819)* mutants balanced with the free duplication *sDp3* and labeled with the ERM-1::GFP transgene were used for the genetic interaction screens [*let-767(s2819) dpy-17(3164); sDp3; perm-1-erm-1-gfp*; the duplication also covers *dpy-17*; hence, only animals that have lost the duplication have a dumpy (Dpy) body morphology; see Fig. 3B, larvae on the right side; table S4 for full genotype]. Screen design and read-out are described in the text and in Fig. 3B legend. *let-767(s2819)* is a maternal-effect larval lethal allele; thus, even homozygous progeny are not complete nulls due to the presence of maternal product (*8*). Although the same *let-767(s2819)* mutant strain was used for enhancer and suppressor screens, each screen was carried out separately. For the enhancer screen, 40 to 60 gravid hermaphrodites were seeded with bleaching solution (4:1 bleach solution/10 M NaOH) onto each RNAi plate. The surviving eggs were allowed to hatch and grow for 42 to 46 hours. The same generation of *let-767(+/−)* animals was evaluated and followed for an additional 2 to 3 days or until they grew into adults. The suppressor screen was carried out by seeding five L4-stage larvae on each RNAi plate. Worms were allowed to grow up and lay eggs on the RNAi plates. After 70 to 74 hours, at least 70 next-generation *let-767(−/−)* dumpy L1 larvae per plate were handpicked onto a new RNAi plate. Their development was followed at least until day 5 to allow for full expression of the ectopic lumen phenotype. In each set of experiments, 50 to 120 live animals per plate were scored for the presence/absence of the intestinal polarity conversion phenotype under a dissecting fluorescence microscope (see below, microscopy), and each experiment was repeated three to five times.

### Fluorescently labeled fusion proteins

All strains carrying fluorescently labeled fusion proteins used in this study were previously generated by us and others and have been described [see table S4 and (*99*, *100*) for technical approach]. All integral and submembranous apical and basolateral membrane proteins used in this study are expressed from their endogenous promotors and are translational fluorophore fusions. We and others have confirmed their subcellular localization by different labeling procedures, including distinct transgenes, antibodies, germline knock-ins, chemical staining, and by their ability to rescue the corresponding mutant phenotype (*99*, *100*). Where available, different fluorophore fusions were tested in parallel for subcellular localization studies of integral membrane proteins (e.g., PEPT-1::DsRed and PEPT-1::GFP; Fig. 4E). The low copy number *erm-1p::erm-1::gfp* transgene, previously characterized and shown to be devoid of any phenotypic effects, is suited for its use as apical domain identity marker since it avoids any disturbance of the *erm-1* germline locus that might interfere with apical domain biogenesis (*8*, *12*, *28*, *32*). ERM-1’s subcellular localization has been previously confirmed by various independent transgenic strains (high and low copy number, labeled with different fluorophores) and by an ERM-1::GFP CRISPR knock-in (*28*, *32*, *104*). The PAR-6::GFP, GFP::PKC-3, and PAR-3:mCherry strains used in this study are CRISPR knock-ins. All vesicle-based fluorescently labeled proteins are translational fusions driven to the intestine by the *vha-6* promotor to allow for the subcellular positional analysis of these ubiquitously expressed molecules (see table S4 for list of strains used in this study).

### DsRed feeding

DsRed HT115 RNAi bacteria (table S4) were generated with a DsRed-expressing plasmid to produce a faint red color. Animals were fed on plates containing RNAi bacteria targeting secretory pathway components and control RNAi bacteria (no vector) for 2 days. At least 70 animals were transferred to plates containing a 1:1 mixture of gene-specific and DsRed-containing RNAi bacteria, at least 15 hours before evaluation.

### BODIPY-ceramide assay

BODIPY-ceramide (Cer) (see table S4) at a final concentration of 1% was added to bacteria seeded onto the RNAi feeding plates. Fifteen hours after seeding, five L3 larvae were placed onto the RNAi plates and their L3 progeny was evaluated microscopically. *unc-22* RNAi was used as a positive control for these RNAi conditions. Before microscopic evaluation, animals were transferred onto OP50 plates without BODIPY-Cer to ensure that their intestinal lumens had cleared off the label and their cuticles were cleaned (animals with BODIPY in their intestinal lumen were excluded from the analysis). One animal was mounted on a slide and individually scanned for each experiment. The time between mounting the animal and taking the image did not exceed 1.5 min. See below (microscopy and quantification) for confocal analysis and fluorescence intensity measurement.

### Enhancement of *let-767(RNAi)*–induced ectopic lumen formation by activated RAB-7

To test for the ability of RAB7Q68L to enhance polarity conversion, mild *let-767* RNAi conditions were empirically determined (see above, RNAi) to generate progeny that developed ectopic lumens in less than 5% at the L1 stage [almost 100% of L1 larvae exposed to *let-767(RNAi)* bacteria under standard RNAi conditions display ectopic lumens]. Ectopic lumens in *let-767(RNAi)* intestines were scored by staining for apical intermediate filaments using the anti–IFB-2 antibody MH33 [IFB-2, like ERM-1, outlines the apical domain and is displaced to basolateral membranes and ectopic lumens during polarity conversion (*8*)]. Presence/absence and the number of ectopic lumens were assessed by confocal microscopy, and the severity of the phenotype was graded from 0, 1, 2, to 3 (no ectopic lumens, few anterior ectopic lumens, multiple anterior ectopic lumens, and ectopic lumens along the length of intestine, respectively). Twenty L1s were examined per condition in three separate sets of experiments.

### Yolk assay

Yolk proteins (YP170) are synthesized in the intestine, secreted into the pseudocoelomic space (body cavity), and then endocytosed by oocytes. In wild-type adult hermaphrodites, YP170::GFP is enriched in oocytes and embryos. Endocytosis defects can be distinguished from defects in secretion by accumulating YP170::GFP in the body cavity versus the intestine (*105*). Five YP170::GFP L4-stage larvae were placed on each RNAi plate using mild RNAi conditions to allow progeny to grow up to hermaphrodites with fully developed gonads (e.g., *vha-6* RNAi bacteria were diluted 1:4 with mock RNAi bacteria). The effectiveness of these mild RNAi conditions was deemed sufficient to assess intestinal trafficking defects since they produced substantial concomitant endocytosis defects in oocytes. YP170::GFP was also evaluated in late larval intestines under standard RNAi conditions, with the same effect as in adult intestines (no intestinal accumulation).

### Colocalization studies of endomembrane and plasma membrane components

Standard RNAi conditions were used to evaluate the effect of suppressor knockdowns on double transgenic animals carrying green- versus red-labeled fluorescent fusion proteins of plasma and endosomal vesicle membranes, respectively. Confocal analysis of L3 and L4 larvae was carried out as described below (microscopy), with the following adjustments (established in pilot experiments to optimize resolution and avoid corruption of signal, loss of signal, and false overlap): Images were taken as five sections along the *z* axis at 0.15-μm intervals, dorsal and ventral of the mid-intestinal plane (lumen), and images were averaged twice; DAPI (4′,6-diamidino-2-phenylindole) exclusion was used to distinguish autofluorescent gut granules, allowing an increase in (Nikon) laser settings to DAPI: high voltage (HV) = 88, 405 = 5.57, fluorescein isothiocyanate (FITC): HV = 50, 488 = 8.76, tetramethyl rhodamine isothiocyanate (TRITC): HV = 85, 561 = 51.88; pinhole was set at 40.0 μm. Only sequential imaging was performed, DAPI last, as described below (microscopy). Vesicles were examined on all single sections. Overlapping signals observed in both red and green but not blue channels were considered bona fide colocalization events (as opposed to autofluorescent signals of *C. elegans* gut granules that appear in all three channels).

### Immunohistochemistry

L1 larvae were collected in M9 medium (*101*) onto slides coated with 0.1 to 0.2% poly-l-lysine (Sigma, P5899), covered with overhanging coverslips, and then permeabilized by flash freezing in liquid nitrogen and subsequent flicking off the coverslip (*12*, *100*). Fixation was performed by sequential incubation in methanol and acetone at −20°C. Immunofluorescent staining was carried out as described [procedures are demonstrated in (*100*)]. For MH33 staining, slides were exposed to the first antibody (1:10 dilution) overnight at 4°C, washed, and then exposed to the secondary FITC-labeled antibody (diluted 1:100) for 1 hour at room temperature. Permount (Fisher, SP15-100) was used as a coverslide mounting medium.

### Microscopy

Typically, live worms were directly scored and phenotypically characterized on their plates under an Olympus SZX12 dissecting microscope equipped with a high-power stereo fluorescence attachment (Kramer Scientific). DIC (Nomarski) and confocal microscopic analysis were carried out using either a Leica TCS SL laser-scanning confocal microscope (Leica Microsystem) or a Nikon C2 laser-scanning confocal mounted on an ECLIPSE Ti-E inverted microscope (Nikon), supplemented with DIC optics. Live worms were mounted and immobilized on glass slides using 10 mM sodium azide (Fisher Scientific, BP9221-500) or 5% lidocaine (MP Biomedicals, LLG, 193917). Single-plane images were taken as 10 (6 to 20) sections along the *z* axis at 0.2-μm or smaller intervals and integrated for projection images. Most images were obtained by a Nikon 60×/1.40 Oil apochromat objective and scanned at 1024 × 1024 pixels. Laser settings were set at lowest possible gain and laser power where not indicated otherwise, and identical settings were used for all comparisons. Autofluorescent gut granules were visually eliminated/reduced by confocal settings (Leica: by restricting the wavelengths of the fluorescence filters: green filter spectrum to 500 to 515 nm, red filter spectrum to 630 to 700 nm; Nikon by using the following settings: DAPI: HV = 108, 405 = 5.57, FITC: HV = 69, 488 = 6.35, TRITC: HV = 105, 561 = 7.62), or the DAPI channel was used for their identification (*105*). For multi-channel images, individual channel intensity was adjusted to achieve equivalent brightness of all channels, and samples were scanned sequentially to eliminate bleed-through between channels. The images were adjusted by Adobe Photoshop software for contrast and brightness without further changes. No deconvolution software was used.

### Transmission electron microscopy

*vha-5*, *vha-6*, *dab-1*, and *sly-1* RNAi was performed by standard conditions (see above, RNAi), using either N2, *fgEx13[erm-1p::erm-1::gfp, rol-6p::rol-6(su1006)]* or *rrf-3(pk1426)II; fgEx13[erm-1p::erm-1::gfp, rol-6p::rol-6(su1006)]* transgenic animals. More than 20 RNAi plates (60 × 15 mm) were set up with five L4 larvae, and progeny was allowed to develop to the mid-larval stage if not arrested earlier. Before harvesting the progeny, animals were checked under a fluorescent dissecting scope for the presence of the phenotype (i.e., presence of vacuolar displacement of the apical marker). Larvae were washed off in standard M9 medium (*101*) and collected into 1.5-ml Eppendorf tubes. They were then fixed in 2.5% glutaraldehyde and 1.0% paraformaldehyde in 0.05 M sodium cacodylate buffer (pH 7.4) plus 3.0% sucrose. Before fixation, the cuticles were “nicked” with a razor blade in a drop of fixative under a dissecting microscope to allow the fixative to penetrate. After an initial 2-hour fixation at room temperature, the specimens were transferred into fresh fixative and stored overnight at 4°C. Specimens were rinsed several times in 0.1 M cacodylate buffer and then postfixed in 1.0% osmium tetroxide in 0.1 M cacodylate buffer for 2 hours on ice. After postfixation, specimens were rinsed several times in 0.1 M cacodylate buffer and then embedded in 2.0% agarose in phosphate-buffered saline (PBS) for ease of handling. The agarose blocks were dehydrated through a graded series of ethanol to 100%, dehydrated briefly in 100% propylene oxide, and pre-infiltrated overnight on a rocker in a 1:1 mixture of propylene oxide:Eponate resin (Ted Pella, Redding, CA). The following day, the agarose blocks were infiltrated in 100% Eponate resin for several hours, then embedded in flat molds in fresh Eponate resin, and allowed to polymerize a minimum of 24 hours at 60°C. Thin sections were cut on a Leica UC7 ultramicrotome and collected on formvar-coated grids, poststained with uranyl acetate and Reynold’s lead citrate, and viewed in a JEOL 1011 TEM at 80 kV equipped with an AMT digital imaging system (Advanced Microscopy Techniques, Danvers, MA).

### Hierarchical clustering

The phenotypic profiles of the 50 identified trafficking genes were analyzed by average linkage agglomerative hierarchical clustering with a centered correlation coefficient as a similarity metric of the gene profile for all genes using Cluster 3.0 software (*106*). Phenotype strength was defined as follows: 0, no phenotype; 1, phenotype identified by extended phenotypic evaluation; 2, phenotype identified by initial tubulogenesis screens. The hierarchical clustering dendrograms were visualized with Java TreeView software (*107*).

### Quantification of BODIPY-Cer vesicles and fluorescence intensity measurement

Before quantification, inspection of worms under a fluorescence dissecting microscope revealed no appreciable difference in quantity, size, and distribution of BODIPY-Cer vesicles between RNAi and control worms at different larval stages. L1 to L4 larvae were tested in several sets of pilot experiments for confocal fluorescence intensity measurements and quantification of BODIPY-Cer vesicles, and L3-stage worms were found to be best suited for standardization (size, RNAi effect, uptake of BODIPY-Cer). Results reported here reflect one set of quantification experiments performed with L3-stage larvae (five L3s per each condition; three separate sets of experiments). For fluorescence intensity measurements, a square area of 250 × 200 pixels was chosen in the anterior intestine, corresponding to four cells (INT II and INT III), and ImageJ [National Institutes of Health (NIH)] software was used for image analysis. For quantification of vesicles, two circles of 100 pixels of the same area were counted.

### Fluorescence intensity measurement and quantification of plasma membrane components

All images were analyzed by ImageJ software. To determine membrane versus cytoplasmic fluorescence intensity ratios between different animals, identical confocal laser settings were used for each set of experiments. Fifty-pixel long lines were drawn orthogonally across each of the four anterior-most lateral membranes between the first-to-second and second-to-third intestinal rings (INT II and III). The mid-point of the line was always placed on the lateral membrane. The medians of the maximum fluorescence intensity values were used for both membranes and the cytoplasm, and the membrane/cytoplasm ratios of the four measured cells were averaged and compared to controls. The average of four membrane/cytoplasm ratios was calculated, and more than seven sets of serial confocal images were analyzed. The average from three independent experiments was used to draw the bar graph. For quantification of the PEPT-1::DsRed, PAR-6::GFP, GFP::PKC-3, and GFP::RAB-10 membrane/membrane-associated/cytoplasm fluorescence intensity ratio, three cells of each worm were used for fluorescence intensity quantification and >10 worms were used to calculate the final membrane/cytoplasm intensity ratio. For quantification of the presence/absence of hTAC on the plasma membrane, the four lateral membranes between the first three anterior intestinal INT rings (INT I/II and INT II/III) of suppressor knockdowns versus controls were examined for the absence, reduction, and presence of hTAC. More than 30 animals were counted per experiment, and each experiment was repeated three times. Note that quantification of fluorophore loss on basolateral membranes (SLCF-1 and hTAC; fig. S7, C and D) underestimates the actual loss since only a fraction of membranes retain sufficient fluorophore to qualify for quantification.

### Quantification of colocalization of endomembrane and plasma membrane components

Animals labeled with fluorescent endomembrane and plasma membrane components were scanned by confocal microscopy (see above, colocalization studies). Per worm, fluorescent puncta were counted separately inside two intestinal cells, outlined by a fluorescently labeled basolateral membrane marker. Puncta were subjectively determined as endosomal vesicles, considering factors such as shape, size, boundary, and signal intensity (all fluorescent markers used were previously determined to reside on endomembranes and/or plasma membranes). Vesicles were classified into three categories: membrane-associated (puncta overlapping with plasma membrane components), tubular (puncta in connection to a cytoplasmic tubular structure that may or may not be connected to the membrane), and cytoplasmic (puncta separated from surrounding structures). Eight animals were counted per each experiment, and more than three independent experiments each were performed.

### Statistics

Statistical analyses were performed by GraphPad Prism 5 software, and the specific tests used for each experiment are listed in the figure legends. All values shown are mean ± SEM of three or more independent experimental data sets. Where pertinent, *N* (sample size) and *n* (number of independent experiments) are indicated in the text and figure legends. *P < 0.05, **P < 0.01, ***P < 0.001.

## Supplementary Materials

### This PDF file includes:

Figs. S1 to S10 Legends for tables S1 to S4 Legend for movie S1 References

## Notes

View/request a protocol for this paper from *Bio-protocol*.
