# Supplementary material for: The biosynthetic-secretory pathway, supplemented by recycling routes, determines epithelial membrane polarity
Source: Sci Adv. 2023 Jun 28;9(26):eade4620. doi: 10.1126/sciadv.ade4620 (PMC10306302; doi:10.1126/sciadv.ade4620)
Supplement: Supplementary file 1 — Figs. S1 to S10 Legends for tables S1 to S4 Legend for movie S1 References [file sciadv.ade4620_sm.pdf]

Science Advances
AAAS

Supplementary Materials for

The biosynthetic-secretory pathway, supplemented by recycling routes, determines epithelial membrane polarity

Nan Zhang *et al.*

Corresponding author: Verena Gobel, vgobel@mgh.harvard.edu

Sci. Adv. **9**, eade4620 (2023)
DOI: 10.1126/sciadv.ade4620

The PDF file includes:

Figs. S1 to S10
Legends for tables S1 to S4
Legend for movie S1
References

Other Supplementary Material for this manuscript includes the following:

Tables S1 to S4
Movie S1

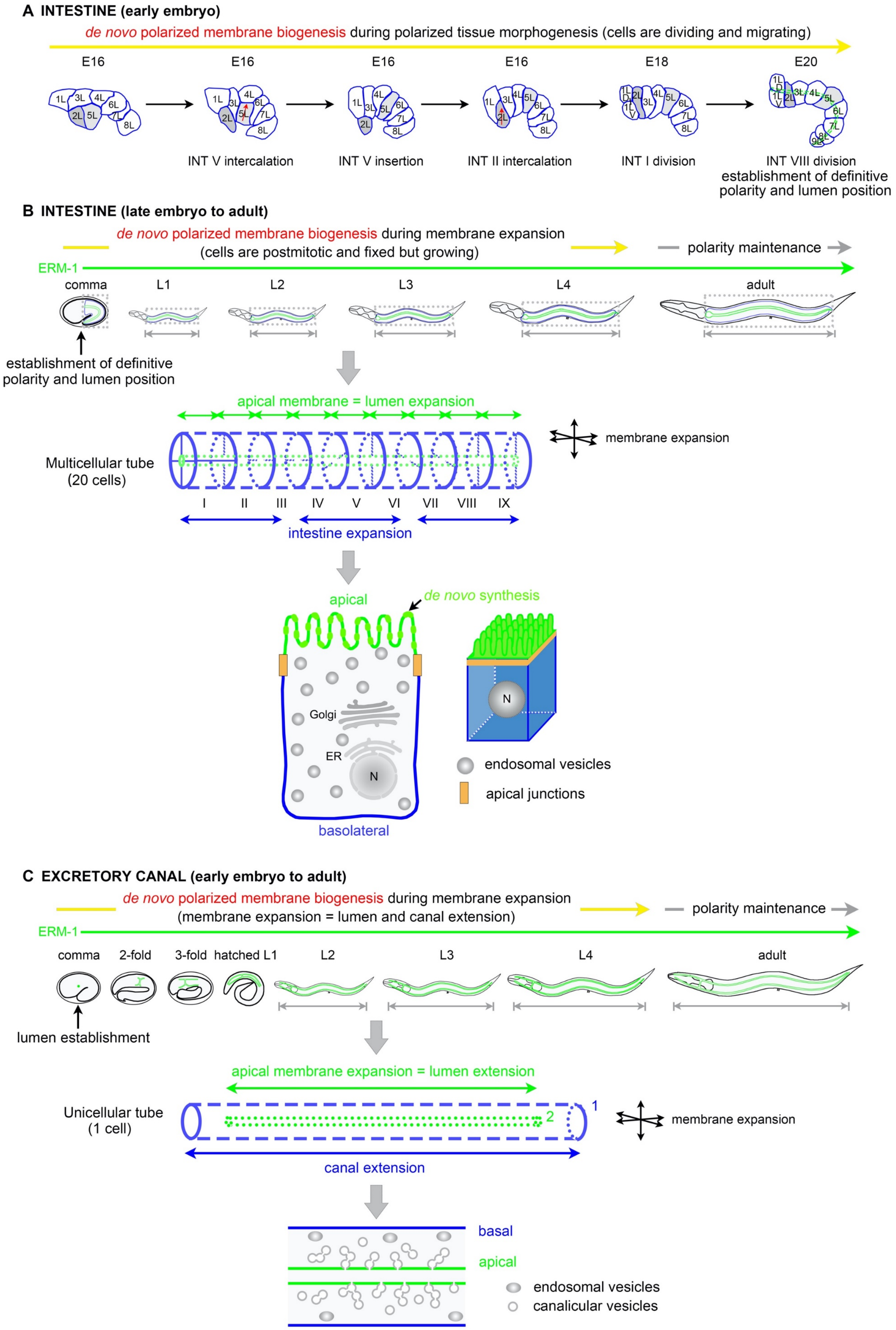
A INTESTINE (early embryo)
de novo polarized membrane biogenesis during polarized tissue morphogenesis (cells are dividing and migrating)
E16
E16
E16
E16
E18
E20
INT V intercalation
INT V insertion
INT II intercalation
INT I division
INT VIII division
establishment of definitive polarity and lumen position
B INTESTINE (late embryo to adult)
de novo polarized membrane biogenesis during membrane expansion
(cells are postmitotic and fixed but growing)
polarity maintenance
ERM-1
comma
L1
L2
L3
L4
adult
establishment of definitive polarity and lumen position
apical membrane = lumen expansion
membrane expansion
Multicellular tube (20 cells)
I
II
III
IV
V
VI
VII
VIII
IX
intestine expansion
apical
de novo synthesis
Golgi
ER
N
basolateral
endosomal vesicles
apical junctions
C EXCRETORY CANAL (early embryo to adult)
de novo polarized membrane biogenesis during membrane expansion
(membrane expansion = lumen and canal extension)
polarity maintenance
ERM-1
comma
2-fold
3-fold
hatched L1
L2
L3
L4
adult
lumen establishment
apical membrane expansion = lumen extension
Unicellular tube (1 cell)
membrane expansion
canal extension
basal
apical
endosomal vesicles
canalicular vesicles

Fig. S1. ***De novo*** **polarized membrane biogenesis during** ***C. elegans*** **intestinal and excretory canal tubulogenesis (related to Fig. 1A).**

Schematics show temporal vectors (developmental time [yellow arrows]) and spatial vectors for polarized membrane expansion during intestinal and excretory canal tubulogenesis. Upper panels indicate net anterior-posterior extension of the apical/lumenal membrane [green arrows], middle panels apical [green arrows], basolateral [blue arrows], and circumferential [black arrows] membrane expansion on the cellular level.

The *C. elegans* intestine is a single-layered multi-cellular epithelial tube and the *C. elegans* excretory canal is a unicellular tube consisting of one single cell. InTERcellular (intestinal) and inTRAcellular (canal) apical membrane and lumen biogenesis occur coincidently in both tubes. The development of these simple tubular organs is thus uniquely suited for the *in vivo* tracking of membrane biogenesis at the single-cell level and for the distinction of apical (=lumenal) from lateral and basal cellular membrane biogenesis within the 3D tissue context of the transparent organism. In postmitotic, immobile cells of growing late-embryonic and larval tubes, *de novo* polarized membrane biogenesis (yellow arrows) can be separated from polarized tissue morphogenesis. This setting permits the analysis of the effects of polarized intracellular processes (e.g., trafficking and cytoskeletal dynamics) on polarized membrane biogenesis independent from these processes' concomitant effects on polarized cell division and migration that occur simultaneously with polarized membrane biogenesis during tissue morphogenesis.

ERM-1, the single *C. elegans* ortholog of the ERM/ezrin-radixin-moesin family of membrane-actin linkers, is asymmetrically positioned at all apical/lumenal membranes of *C. elegans* tubular internal organ epithelia that do not secret cuticle (*32*). In tubular epithelia of many species, ERMs denote membranes with apical character, poised to form apical-membrane specific microdomains such as microvilli (*29, 30*). ERM-1 tracks apical/lumenal membrane biogenesis from the time of the establishment of the definitive apicobasal polarity and lumen position in the *C. elegans* intestine and the time of lumen initiation in the *C. elegans* excretory canal in the early embryo. ERM-1 continues to mark the apical/lumenal domain throughout its further expansion during postmitotic tube growth (late embryo and four larval stages), and during its maintenance in the fully grown adult tube.

(A) Intestine (early embryo). Coincident polarized membrane and tissue morphogenesis at the time of polarity establishment (early embryo: pre-bean to early comma stage). The 20 intestinal cells are clonally derived from a single progenitor (E cell). The establishment of the definitive apicobasal polarity occurs at the E16 stage, where cytoplasmic polarization (movement of nuclei to the midline=future apical/lumenal membrane) precedes membrane polarization and intercalation (*45, 46*). Intercalation transforms the intestinal tube from a double-layered into a single-layered tubular epithelium by one intercalation step during which some cells still divide. Cells of the lower tier extend their apical membranes and move into the upper tier, with right and left cells moving in parallel (the left-sided [L] cells of the future INT I-VIII doublets are shown). The mature intestinal tube has bilateral symmetry, with 8 INT rings consisting of two cells each (INT II-IX) and the first INT ring consisting of four cells (INT I; see Fig. 1A).

(B) Intestine (late embryo to adult). Coincident apical membrane and lumen biogenesis between pairs of expanding post-mitotic cells (later embryo: late comma- through 1.5-, 2-, and 3-fold embryo [not shown], and through four larval stages, L1 - L4, to adult). The tube's apical membrane (green) faces and concomitantly builds the lumen, while the basolateral membrane (blue) contacts neighboring cells or the body cavity (top and middle diagrams); apical junctions separate the polarized domains (orange; bottom diagrams of a single cell). *De novo* polarized membrane biogenesis starts in still dividing and migrating cells (shortly before the comma stage) and continues in postmitotic cells that expand at their fixed positions within the mature organ (in late embryos, through four larval stages, to adulthood; minimal membrane expansion in adults; top diagram). The anterior-posterior length of the apical/lumenal intestinal membrane increases from a mean of 21.6 μm to a mean of 735.1 μm from the bean-stage early embryo to the adult (*51*). In addition, a single cell's fully polarized apical domain requires a multiple of its ~1/6th overall cellular membrane surface for microvilli formation (3D view, bottom diagram). The location and size of intracellular organelles (bottom diagram) are not drawn to scale. Golgi mini-stacks are distributed throughout the cytoplasm in the adult epithelium (*55*). N=nucleus.

(C) Excretory canal (early embryo to adult). Coincident apical membrane and lumen biogenesis inside the expanding, postmitotic single-cell excretory canal. During early embryogenesis, the excretory cell migrates to its definitive location at the left lateral side of the posterior pharyngeal bulb, from where it sends out two extensions to the left and right lateral surfaces (top diagrams) (*111*). At the lateral hypodermis, each canal extension bifurcates and grows anterior-ward and posterior-ward (top diagrams; compare with Fig. 1A). Canals grow directionally at a speed exceeding that of the animal until the late L1-larval stage when they reach the tips of the worm's nose and tail. The posterior branches are extended halfway through the animal's length at hatching. The apical membrane (green) expands as an endotube (intracellular lumen) within the cell body and its expansion follows the expansion of the basal membrane (blue; middle diagram: basal membrane: 1, apical membrane: 2). The excretory cell lumen is connected to the lumen of the adjacent duct cell, where apical and basal membranes meet, separated by a single junction; canals are otherwise junction-less. *De novo* apical membrane biogenesis in the excretory canal continues from lumen initiation in the early embryo through embryonic and larval stages until adulthood, with the length of the apical/lumenal membrane increasing to a total of ~2000 μm (corresponding to an anterior-posterior extension that traverses the length of the animal twice). The enlarged view of a canal arm section (bottom diagram) shows canalicular and endosomal vesicles, the former partially interconnected and connected to the apical/lumenal membrane.

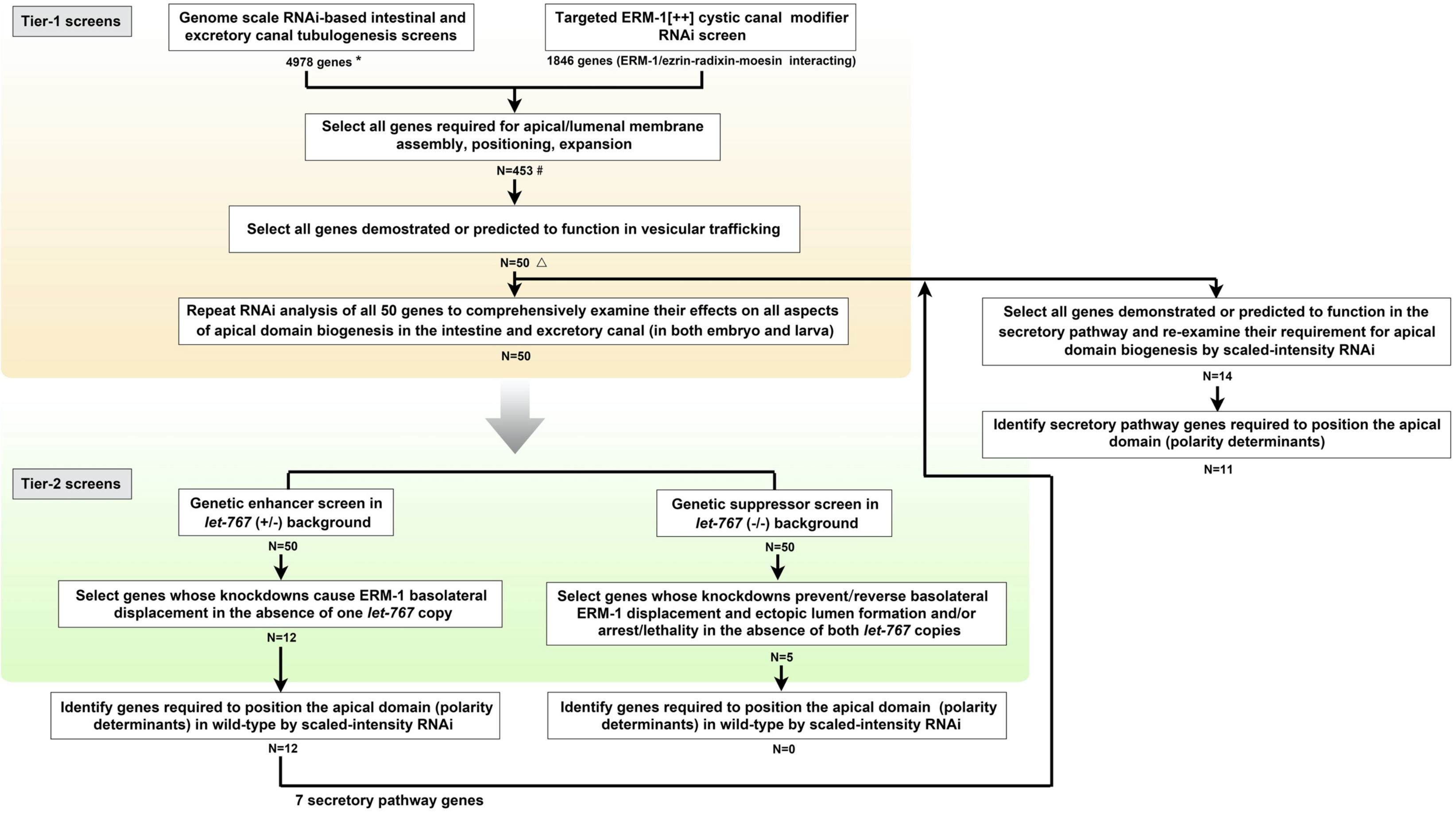

* (1) All chromosome III genes (pilot screen); (2) All lethal genes (shown to harbor >90% tubulogenesis phenotypes in pilot screen)

\# 41-genes dentified in both the intestinal and canal screen

△ Note that previously characterized GSL-biosynthetic and clathrin/AP-1 subunit genes are not included

Fig. S2. Flowchart of the two-tiered *C. elegans* genomics-genetics screens, including the flow of subsequent selection steps and sub-screens (related to Fig. 1B).

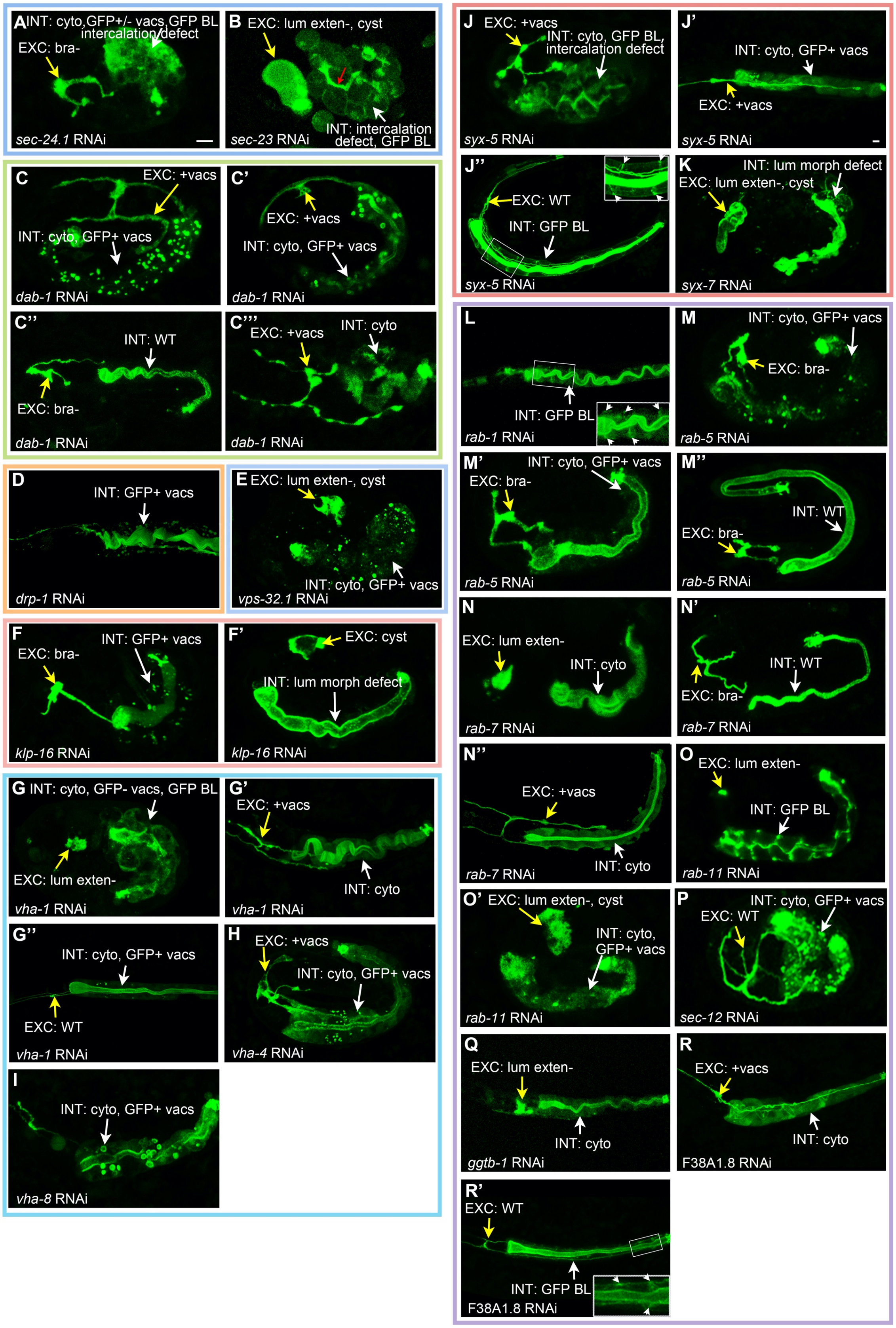
A INT: cyto, GFP+/- vacs, GFP BL intercalation defect
EXC: bra-
sec-24.1 RNAi
B EXC: lum exten-, cyst
INT: intercalation defect, GFP BL
sec-23 RNAi
C EXC: +vacs
INT: cyto, GFP+ vacs
dab-1 RNAi
C' EXC: +vacs
INT: cyto, GFP+ vacs
dab-1 RNAi
C'' INT: WT
EXC: bra-
dab-1 RNAi
C''' EXC: +vacs
INT: cyto
dab-1 RNAi
D INT: GFP+ vacs
drp-1 RNAi
E EXC: lum exten-, cyst
INT: cyto, GFP+ vacs
vps-32.1 RNAi
F EXC: bra-
INT: GFP+ vacs
klp-16 RNAi
F' EXC: cyst
INT: lum morph defect
klp-16 RNAi
G INT: cyto, GFP- vacs, GFP BL
EXC: lum exten-
vha-1 RNAi
G' EXC: +vacs
INT: cyto
vha-1 RNAi
G'' INT: cyto, GFP+ vacs
EXC: WT
vha-1 RNAi
H EXC: +vacs
INT: cyto, GFP+ vacs
vha-4 RNAi
I INT: cyto, GFP+ vacs
vha-8 RNAi
J EXC: +vacs
INT: cyto, GFP BL, intercalation defect
syx-5 RNAi
J' INT: cyto, GFP+ vacs
EXC: +vacs
syx-5 RNAi
J'' EXC: WT
INT: GFP BL
syx-5 RNAi
K INT: lum morph defect
EXC: lum exten-, cyst
syx-7 RNAi
L INT: GFP BL
rab-1 RNAi
M INT: cyto, GFP+ vacs
EXC: bra-
rab-5 RNAi
M' INT: cyto, GFP+ vacs
EXC: bra-
rab-5 RNAi
M'' INT: WT
EXC: bra-
rab-5 RNAi
N EXC: lum exten-
INT: cyto
rab-7 RNAi
N' INT: WT
EXC: bra-
rab-7 RNAi
N'' EXC: +vacs
INT: cyto
rab-7 RNAi
O EXC: lum exten-
INT: GFP BL
rab-11 RNAi
O' EXC: lum exten-, cyst
INT: cyto, GFP+ vacs
rab-11 RNAi
P INT: cyto, GFP+ vacs
EXC: WT
sec-12 RNAi
Q EXC: lum exten-
INT: cyto
ggtb-1 RNAi
R EXC: +vacs
INT: cyto
F38A1.8 RNAi
R' EXC: WT
INT: GFP BL
F38A1.8 RNAi

Fig. S3. A shared set of trafficking molecules directs inTER- and inTRAcellular apical membrane assembly, positioning, and expansion (related to Fig. 2B).

Selection of additional apical membrane domain biogenesis defects and description of phenotypes (see Fig. 1B and 2A and table S1 for phenotype classes ① - ⑩ of all 50 genes, Fig. 1A for intestinal [INT] and excretory-canal [EXC] anatomy and fig. S1 for INT and EXC tubulogenesis). Confocal projections of embryonic and larval phenotypes are shown (embryos: A-C''', E-G, H-J, K, M-N', O-P, among them early: A-B, G, J, all others late; L1-larvae: D, G'-G", J'-J'', L, N", Q-R'), with ERM-1::GFP marking the apical domain/lumen in both INT and EXC. See Figs. 1 and 2 for acronyms for phenotypes and for functional classes of molecules (classes are outlined in color); Fig. 2B for wild-type body plan of embryos/larvae and corresponding wild-type INT and EXC ERM-1::GFP localization. Arrows point to selected defects only, see the description below for details. Not all phenotypes are shown. White arrows point to the INT, and yellow arrows to the EXC. Arrowheads: basolateral (BL) displacement. Color coding of functional classes (boxes) as in Figs. 1C and 2. Scale bars: 5µm.

Note that RNAi can be titrated to generate a spectrum of dose-dependent membrane biogenesis phenotypes (Materials and Methods). The spectrum of phenotypes shown here ranges from severe/moderate ('no/partial apical membrane assembly' = full/partial ERM-1 mislocalization in the INT and 'no/short lumen or no/incomplete apical membrane expansion' = 'no ERM-1 extension' in the EXC) to wild-type INT or EXC apical domain/lumen biogenesis. Also note that basolateral (BL) ERM-1 mislocalization, with or without residual apical ERM-1 (e.g., 'pan-membranous ERM-1'), indicates a defect in 'apical domain positioning' and hence indicates a *bona fide* apicobasal polarity defect - distinct from defects in 'apical membrane domain assembly' (absence of ERM-1 at the apical domain). Full displacement of ERM-1 from the membrane masks this distinction, since it can include either or both, a defect in 'apical membrane assembly' and/or in 'apical domain positioning' (*dab-1* provides an example for the former, *sec-23/sec-24.1* for the latter phenotype). An 'allelic series' of increasingly severe phenotypes, generated by a scaled-intensity RNAi approach, can distinguish between these possibilities. 36/50 images in Fig. 2B show combined INT/EXC phenotypes, with 16/36 canals revealing 'no/minimal lumen/ERM-1+ apical membrane extension'.

Narrative (main Fig. 2B images are included; not all aspects of phenotypes are described). **(A-B)** *sec-23/24.1*: early-embryonic apical membrane assembly, positioning, and expansion defects (including images in Fig. 2B). INT: no ERM-1 at the apical domain (no apical domain assembly) in all images (including those of Fig. 2B) but partial apical ERM-1 in *sec-23(RNAi)* INT in this figure ((B); red arrow), with ERM-1 displacement to the cytoplasm and all sides of the membrane (apical domain positioning defect: no apicobasal membrane polarity); EXC: no lumen extension, cystic deformation, short lumen/lumen arms (no apical membrane expansion). **(C-C''')** *dab-1*: spectrum of late embryonic to early larval apical membrane biogenesis but not positioning defects (including image in Fig. 2B). INT/EXC: decreasing severity of vacuolar ERM-1 displacement, from full displacement (no apical domain assembly) to no or homogeneous cytoplasmic ERM-1 displacement (Fig. 2B and fig. S3, C to C"'). Note the absence of BL ERM-1 mislocalization (absence of apicobasal membrane polarity defect). **(D)** *drp-1*: mild apical membrane domain assembly defect. INT: small-vesicular cytoplasmic ERM-1 displacement (compare to large vacuolar ERM-1 displacement in *dab-1* and *vha-8(RNAi)* INTs).

(E) *vps-32.1*: embryonic apical membrane assembly but not positioning defects. INT: full vacuolar ERM-1 displacement from the apical domain but no appearance at the BL membrane (compare to *dab-1*). **(F-F')** *klp-16*: spectrum of embryonic apical membrane assembly and expansion but no positioning (=no apicobasal polarity) defect (including image in Fig. 2B). The spectrum ranges from full vacuolar ERM-1 displacement (no apical domain assembly, Fig. 2B) to mild vacuolar displacement and partial apical domain assembly (F) to full apical domain assembly (F') in the INT; and from no lumen extension (Fig. 2B) to varying degrees of extension defects (EXC; F-F'). **(G-H)** *vha-1/4*: spectrum of early-embryonic to larval apical membrane assembly and positioning defects. INT: combination of cytoplasmic vacuoles with ERM-1-positive versus ERM-1-negative membranes. EXC: cytoplasmic ERM-1 displacement reveals previously noted enlarged and persistent varicosities in short canals (*28*). **(I)** *vha-8:* apical membrane assembly but no positioning defects. INT: note large vacuolar ERM-1 displacement, distinct from *vha-1/4* (see text for the discussion of discrepancy to (*39*)). **(J – J")** *syx-5*: spectrum of early-embryonic to larval apical membrane assembly and positioning defects (including image in Fig. 2B). INT: both cytoplasmic (vacuolar; (J') and Fig. 2B) and BL ERM-1 mislocalization in embryo (J), predominant BL mislocalization in larvae (J''). EXC: mildly reduced lumen extension (Fig. 2B) and cytoplasmic ERM-1 displacement (J-J'). **(K)** *syx-7*: defect during a late step of apical domain assembly and apical membrane expansion defect. INT: near complete apical membrane domain assembly. EXC: minimal lumen extension and cystic deformation (EXC should be extended beyond bifurcation at this stage; compare to similar EXC phenotypes in *sec-23* and *rab-11(RNAi)* animals). No apicobasal polarity defects shown in these images. **(L)** *rab-1*: spectrum of late-embryonic (Fig. 2B) to larval apical domain assembly, positioning, and expansion defects. INT: ERM-1 displacement to cytoplasmic vacuoles and BL membrane in the embryo (Fig. 2B); mild BL displacement in the larva. EXC: mild extension defect (Fig. 2B). **(M-M'')** *rab-5:* spectrum of late-embryonic apical membrane assembly and expansion defects (including image in Fig. 2B). INT: full vacuolar ERM-1 displacement (M); mild cytoplasmic ERM-1 displacement (M'; Fig. 2B); wild-type apical ERM-1 (M''). No apicobasal membrane polarity defect. EXC: no lumen extension (Fig. 2B); lumen branch loss (M-M''). **(N-N'')** *rab-7*: spectrum of late-embryonic apical membrane assembly and expansion defects (including image in Fig. 2B). INT: decreasing severity from: almost full vacuolar ERM-1 displacement (Fig. 2B); cytoplasmic ERM-1 displacement (N, N"); apical ERM-1 (N'). No membrane polarity defect. EXC: no lumen extension (N); short lumen (N'; Fig. 2B); full lumen extension (N''). **(O-O')** *rab-11*: late-embryonic apical membrane assembly, positioning, and expansion defect. INT: strong cytoplasmic ERM-1 displacement (O'); BL ERM-1 is only transiently detected during late-embryonic apical membrane biogenesis (O). EXC: no lumen extension. **(P)** *sec-12*: INT-specific apical membrane assembly and positioning defects in the early and late embryo (including image in Fig. 2B). INT: no apical membrane assembly in early embryo (P); cytoplasmic and BL ERM-1 displacement in late embryo (=apicobasal polarity defect; Fig. 2B); wild-type EXC in these images (posterior arms twisted in the section shown in (P)). **(Q)** *ggtb-1*: discordant severity of INT/EXC apical membrane biogenesis defects in early larvae (including image in Fig. 2B). Strong vacuolar displacement (INT) and wild-type EXC (Fig. 2B) versus mild cytoplasmic displacement (INT) and short lumen (EXC; (Q); no membrane polarity defects shown in these images). **(R-R')** F38A1.8: spectrum of early-embryonic to larval apical membrane assembly and positioning defects (including image in Fig. 2B). INT phenotypes range from cytoplasmic-vacuolar ERM-1 displacement in early-embryo (Fig. 2B) to strong cytoplasmic- (R) and mild BL ERM-1 displacement in larvae (R').

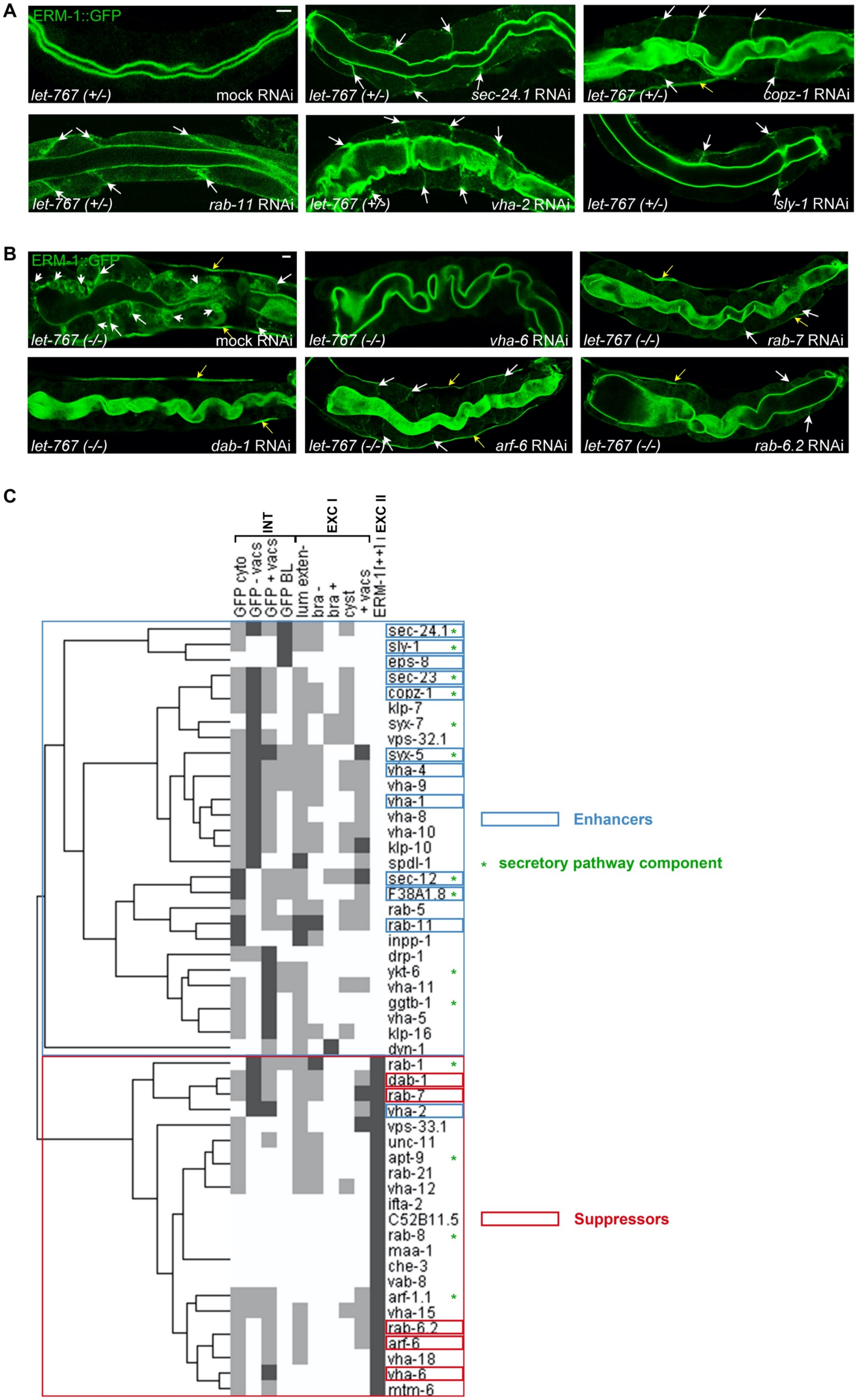
A
ERM-1::GFP
let-767 (+/-)
mock RNAi
let-767 (+/-)
sec-24.1 RNAi
let-767 (+/-)
copz-1 RNAi
let-767 (+/-)
rab-11 RNAi
let-767 (+/-)
vha-2 RNAi
let-767 (+/-)
sly-1 RNAi
B
ERM-1::GFP
let-767 (-/-)
mock RNAi
let-767 (-/-)
vha-6 RNAi
let-767 (-/-)
rab-7 RNAi
let-767 (-/-)
dab-1 RNAi
let-767 (-/-)
arf-6 RNAi
let-767 (-/-)
rab-6.2 RNAi
C
INT
EXC I
EXC II
GFP cyto
GFP - vacs
GFP + vacs
GFP BL
lum exten-
bra -
bra +
cyst
+ vacs
ERM-1[++]
sec-24.1 *
sly-1 *
eps-8
sec-23 *
copz-1 *
klp-7
syx-7 *
vps-32.1
syx-5 *
vha-4
vha-9
vha-1
vha-8
vha-10
klp-10
spdl-1
sec-12 *
F38A1.8 *
rab-5
rab-11
inpp-1
drp-1
ykt-6 *
vha-11
ggtb-1 *
vha-5
klp-16
dyn-1
rab-1 *
dab-1
rab-7
vha-2
vps-33.1
unc-11
apt-9 *
rab-21
vha-12
ifta-2
C52B11.5
rab-8 *
maa-1
che-3
vab-8
arf-1.1 *
vha-15
rab-6.2
arf-6
vha-18
vha-6
mtm-6
Enhancers
* secretory pathway component
Suppressors

Fig. S4. Genetic interaction screens identify 17 modifiers of the GSL-dependent apicobasal polarity conversion (related to Fig. 3).

(A) Enhancement (compare Fig. 3B). Additional examples of *de novo* appearance of basolateral (BL) ERM-1::GFP (arrows) in growing double *let-767(+/-)/enhancer(RNAi)* larval intestines on day 3 post-RNAi induction (Materials and Methods). Note intact polarity with strict apical confinement of ERM-1::GFP in *let-767(+/-)* haplosufficient mutant background (*sDp3* duplication provides one *let-767* copy; upper left image). Arrows indicate lateral ERM-1::GFP mislocalization and basal ERM-1::GFP mislocalization, outlining the intestine. Note the punctate nature of BL ERM-1::GFP recruitment with additional cytoplasmic ERM-1::GFP puncta in the vicinity of the membrane.

(B) Suppression (compare Fig. 3B). *vha-6*, *rab-7*, *dab-1*, *arf-6* and *rab-6.2* RNAi suppress BL ERM-1::GFP mislocalization and BL ectopic lumen formation in *let-767(-/-)* intestines. On day 5 post-RNAi induction (standard RNAi, Materials and Methods), greater than 90% of *let-767(-/-)* mutants (no *let-767* copy, *sDp3* duplication lost; upper left image) display BL ERM-1::GFP displacement (arrows) and BL ectopic lumens (small arrows). In double mutant/RNAi animals (all other images) ERM-1::GFP is confined to the apical membrane (*vha-6*, *dab-1*) and/or only mildly displaced to BL membranes (*rab-7*, *arf-6, rab-6.2*; representative day 5 images are shown). The brightness of images of double mutant/RNAi intestines is increased to show the absence/reduction of BL ERM-1::GFP (resulting in the false appearance of an ERM-1::GFP increase at the apical domain). Animals are ‘dumpy’ (short and fat), and lumens are wide due to a *dpy-17* mutation, revealed by loss of the duplication (see Material and Methods for strain details).

Confocal sections of larval intestines are shown throughout. Yellow arrows: EXC. Scale bars: 5μm.

(C) Hierarchical clustering of phenotype profiles (Materials and Methods). Most enhancers (blue rectangles) were recovered from INT screens and all suppressors (red rectangles) from EXC screens. Secretory pathway components (green asterisk) track with phenotype signatures of enhancers. Compare Figs. 1 and 2 for acronyms and phenotype classes.

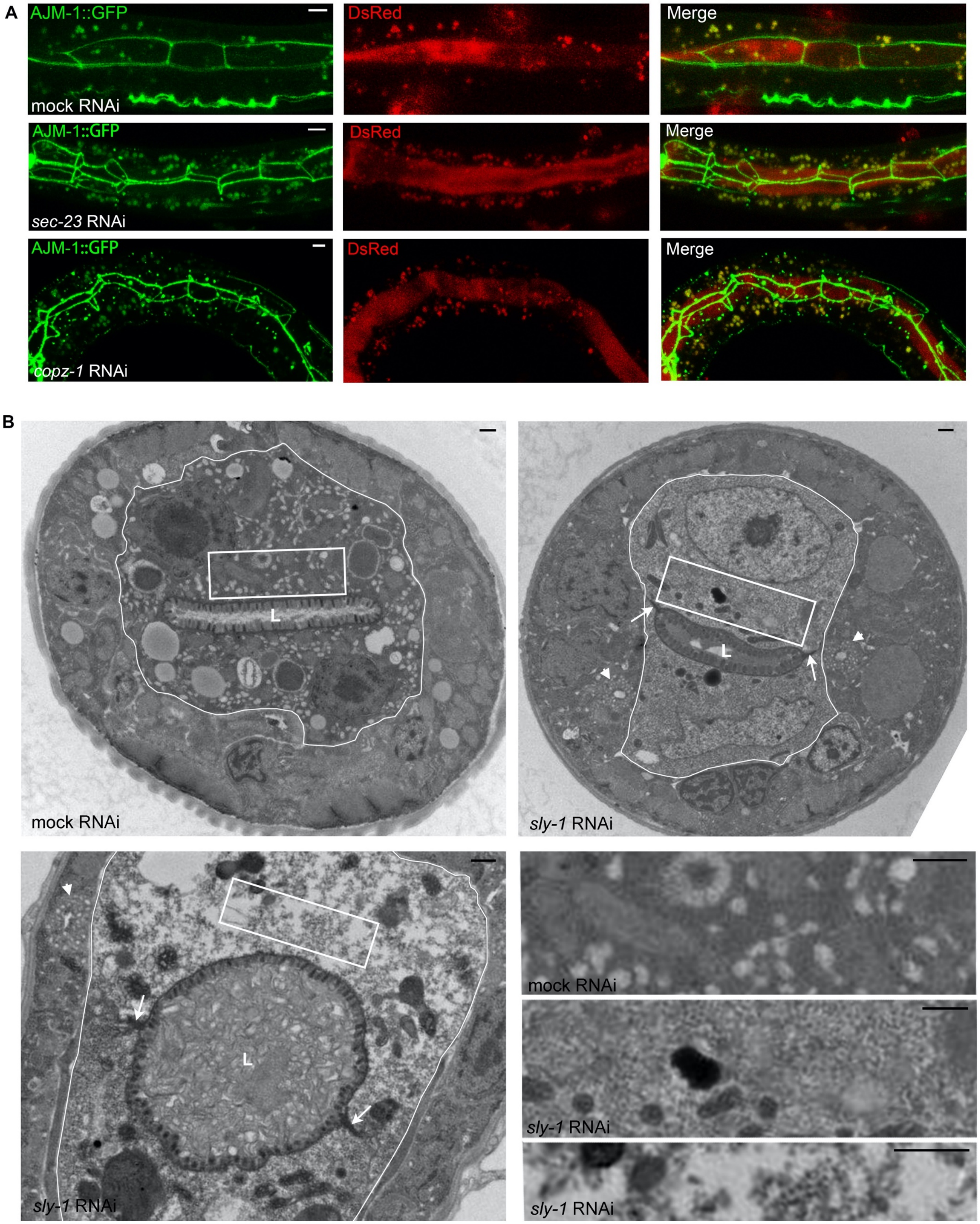
A
AJM-1::GFP
DsRed
Merge
mock RNAi
AJM-1::GFP
DsRed
Merge
sec-23 RNAi
AJM-1::GFP
DsRed
Merge
copz-1 RNAi
B
L
mock RNAi
L
sly-1 RNAi
L
sly-1 RNAi
mock RNAi
sly-1 RNAi
sly-1 RNAi

Fig. S5. Effects of early secretory pathway disruption on the assembly of apical junctions and endomembranes (related to Fig. 4).

(A) Intact apicolateral junctions but increase in cytoplasmic junction material in intestines mildly depleted of secretory pathway components. *sec-23-* and *copz-1(mildRNAi)* larvae, fed with DsRed-labeled bacteria, maintain contiguity of AJM-1-labeled apicolateral junctions that appear functionally intact (no leakage of DsRed between lateral membranes). Concomitant increase of punctate junction material (bright GFP spots) in *copz-1(mildRNAi)* intestinal cytoplasm (a few such puncta are also seen in wild-type; see Fig. 4H). Junctions are occasionally broadened into the lateral membrane (indicated by arrows in Fig. 4H), here obscured by distorted but contiguous apical junction pattern. For junction contiguity in these areas, see rotation of 3D projection (movie S1). Enlarged autofluorescent gut granules can be distinguished by their appearance in both green and red channels (yellow in overlay).

(B) Transmission electron microscopic (TEM) images of cross sections of wild-type and *sly-1(RNAi)* larvae. A white line delineates the intestine (two cells are visualized). Mild (upper right) and moderately-severe (lower left) *sly-1(RNAi)* phenotypes are shown. Note apical/lumenal membrane defects with reduced number and length of microvilli; widened lumen (L); marked paucity of all vesicle- (endo) membranes. Apicolateral junctions appear structurally intact (arrows). Representative sections are shown (75 random sections of *sly-1(RNAi)* L1 larval intestines were evaluated; compare Fig. 4I). Increasing disintegration of endomembranes in *sly-1(RNAi)* cytoplasm shown at bottom right at higher magnification (corresponding to boxed areas in cross sections). Note that the effects of *sly-1* RNAi are largely confined to the intestines. Normal appearing cross sections of excretory canal arm lumens and surrounding canalicular vesicles in these two *sly-1(RNAi)* larvae (arrowheads).

Confocal projections of portions of larval intestines are shown in (A). Scale bars, 5µm in (A); 500nm in (B).

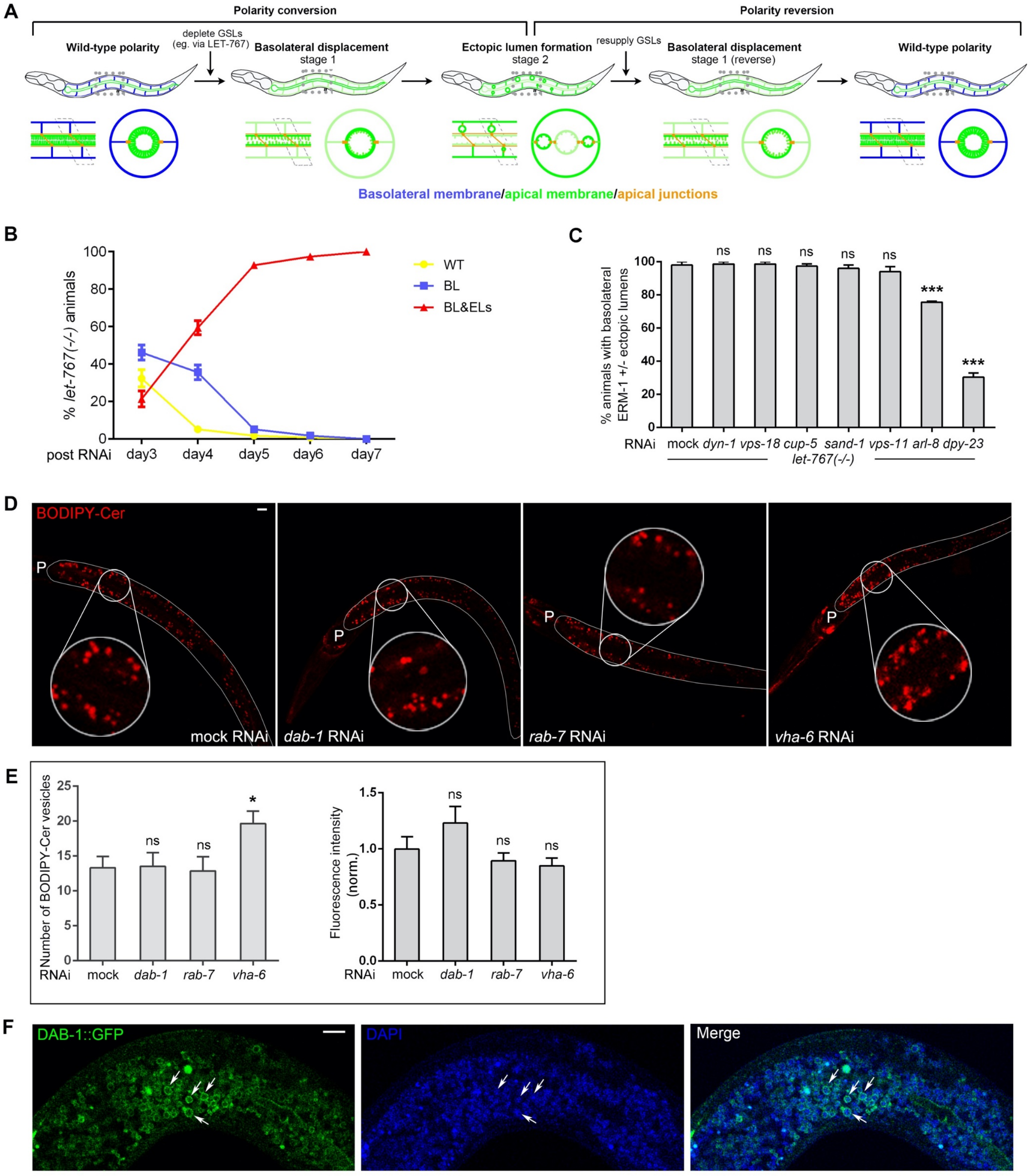
A
Polarity conversion
Polarity reversion
Wild-type polarity
deplete GSLs (eg. via LET-767)
Basolateral displacement stage 1
Ectopic lumen formation stage 2
resupply GSLs
Basolateral displacement stage 1 (reverse)
Wild-type polarity
Basolateral membrane/apical membrane/apical junctions
B
% let-767(-/-) animals
WT
BL
BL&ELs
post RNAi day3 day4 day5 day6 day7
C
% animals with basolateral ERM-1 +/- ectopic lumens
RNAi mock dyn-1 vps-18 cup-5 sand-1 vps-11 arl-8 dpy-23
let-767(-/-)
ns
***
D
BODIPY-Cer
P
mock RNAi
dab-1 RNAi
rab-7 RNAi
vha-6 RNAi
E
Number of BODIPY-Cer vesicles
RNAi mock dab-1 rab-7 vha-6
Fluorescence intensity (norm.)
F
DAB-1::GFP
DAPI
Merge

Fig. S6. Analysis of suppression (related to Fig. 7).

(A) Schematic of apicobasal polarity conversion and reversion in postmitotic cells of the mature but growing larval *C. elegans* intestine, induced by, respectively, depletion and repletion of GSL biosynthesis (see text; Materials and Methods). Growth in GSL-depleted animals is delayed, extending the window for the observation of polarized membrane expansion. From left to right (1-5):

(1) wild-type apicobasal membrane polarity;
(2) stage 1 polarity conversion: basolateral (BL) mislocalization of apical membrane components (day 2-3 post standard RNAi induction; slowly growing L1-larvae);
(3) stage 2: ectopic apical membrane biogenesis (at the site of the initial BL membrane) generates BL lumens with apical-membrane specific microvilli, sub-membranous terminal web and surrounding junctions (day 3-5; larvae arrest at L1 stage);
(4) stage 1 (reverse) polarity reversion after restoring GSL biosynthesis: BL mislocalization of the apical domain without ectopic lumen formation;
(5) restoration of wild-type apicobasal polarity and growth (larvae develop into fertile adults).

GSL depletion/repletion can be modulated by RNAi with various GSL biosynthetic enzymes (*8*). GSL biosynthesis is reduced by placing animals on RNAi bacteria and resumed by removing them from RNAi bacteria to wild-type (OP50) feeding plates. Speed and strength of conversion and reversion can be further modulated by the timing of placing and removing animals from RNAi plates and by different RNAi conditions (Materials and Methods; standard *let-767* RNAi conditions were used in the suppressor analysis).

Top: whole animal; bottom: longitudinal (left) and transverse section (right) through one INT ring. Animals are not drawn to scale (they arrest during polarity conversion and resume growth during reversion).

(B) Time-course of polarity conversion in *let-767(-/-)* larval intestines (compare to Fig. 7C). WT, wild-type; BL, basolateral displacement only; BL&ELs, basolateral displacement and basolateral ectopic lumens.

(C) Disruption of endocytic and degradative trafficking pathways in *let-767(-/-)* mutants. The GTPase DYN-1/dynamin and DPY-23, the mu2 subunit of the clathrin AP2 adaptor (components of endocytic routes), and the mucolipin ortholog CUP-5, the Arf-like GTPase ARL-8, SAND-1/MON1 and the HOPS complex components VPS-11 and VPS-18 (components of vesicle degradative trafficking routes) (*55*) were depleted in *let-767(-/-)* animals using the same conditions as those used in the suppressor screen (compare Fig. 3B; milder RNAi conditions were used for *dyn-1* to avoid sterility; Materials and Methods). Depletion of VPS-33.1, another HOPS complex component (initially identified in tier-1 screens), also failed to suppress, as demonstrated in tier-2 genetic interaction screens (Fig. 2, table S1). Mean ± SEM is shown, n=3; N>30. One-tailed Student's *t*-test was used to calculate *P* values. ns, not significant, ****P*< 0.001.

(D) BODIPY-Cer+ vesicles in *dab-1-, rab-7-* and *vha-6(RNAi)* larval intestines. Note similar contribution and size of BODIPY-Cer+ vesicles in all intestines. P = pharynx. White lines outline intestines. BODIPY-Cer in head area labels amphids.

(E) Quantification of BODIPY-Cer+ vesicles and fluorescence intensity measurements in *dab-1-, rab-7-* and *vha-6(RNAi)* larval intestines. Ceramide (Cer) is the chemical backbone of glycosphingolipids (GSLs). Animals were fed BODIPY-Cer, previously shown to reproduce localization and behavior of endogenous intestinal vesicle-associated GSLs (*12*). Left graph shows the number of intestinal BODIPY-Cer+ vesicles counted in the defined area circled in the images above (D), right graph the corresponding fluorescence intensity. Note the increase in number of BODIPY-Cer+ vesicles but not fluorescence intensity in *vha-6(RNAi)* intestines (compare to (D)). Mean ± SEM is shown, n=3; N=5. Two-tailed Student's *t*-test was used to calculate *P* values. ns, not significant, $*P < 0.05$.

(F) Vesicular localization of DAB-1. DAB-1::GFP resides on at least two morphologically distinct vesicle populations, one of them visible in the DAPI channel. Note GFP ring around blue vesicles (arrows; compare Fig. 7F).

Confocal images of larvae are shown in (D), pair of two cells surrounding the lumen of larval intestines in (F). Scale bars: 5µm.

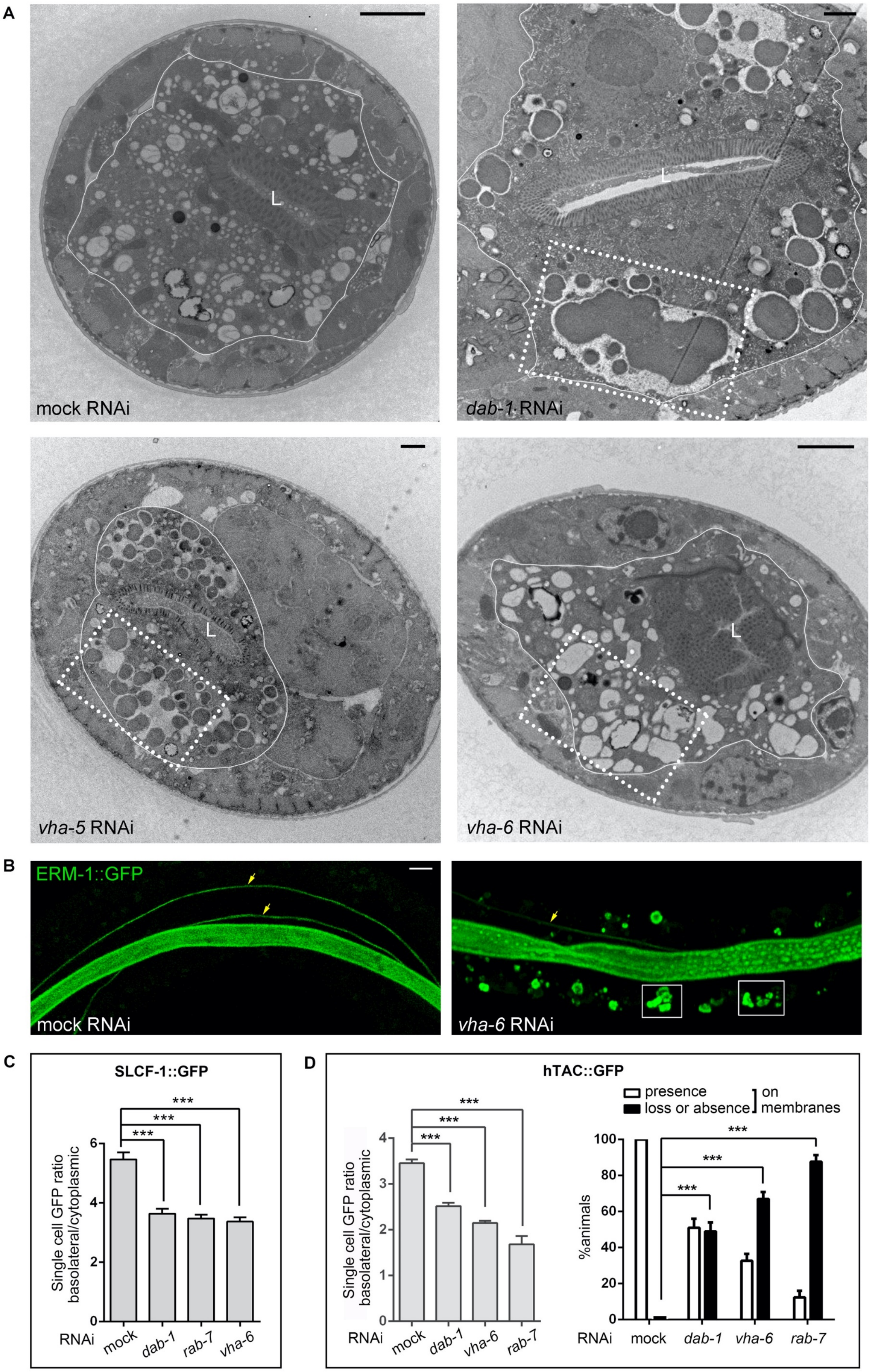
A
L
mock RNAi
L
dab-1 RNAi
L
vha-5 RNAi
L
vha-6 RNAi
B
ERM-1::GFP
mock RNAi
vha-6 RNAi
C
SLCF-1::GFP
***
Single cell GFP ratio basolateral/cytoplasmic
0
2
4
6
RNAi
mock
dab-1
rab-7
vha-6
D
hTAC::GFP
presence
loss or absence
on membranes
***
Single cell GFP ratio basolateral/cytoplasmic
0
1
2
3
4
RNAi
mock
dab-1
vha-6
rab-7
%animals
0
20
40
60
80
100
RNAi
mock
dab-1
vha-6
rab-7

Fig. S7. Cytoplasmic apical membrane inclusions in *dab-1-* and *vha-6(RNAi)* intestines are not ectopic lumens. Quantification of the suppressors' effect on wild-type basolateral membrane biogenesis and recycling (related to Fig. 8).

(A) Representative TEM cross sections of whole *dab-1-, vha-5- and vha-6(RNAi) rrf-3* larval intestines. VHA-5, a paralog of VHA-6, is another V-ATPase 'a' subunit isoform and was also identified in tier-1 screens (table S1). *vha-5-* and *vha-6* contain stretches of >18bp sequence homology required for effective RNAi. The *vha-5(RNAi)* defect copies the *vha-6(RNAi)* intestinal defect. *vha-5* RNAi in a *rrf-3* RNAi-sensitive background generates a high percentage of informative phenotypes in larvae, facilitating TEM studies. Images show similar vacuolar aggregates in all three knockdowns (boxed), with a mixture of amorphous material and intact vesicles, some of which may contain different lipids (N>50; random sections each were evaluated; representative images are shown). Subcellular localization, size, and clustering of vacuolar aggregates, none present in wild-type, suggest they correspond to the ERM-1::GFP+ vacuolar clusters that are also absent in wild-type (compare to B). No inward-pointing microvilli were detected in any of these vacuoles in serial TEM sections, suggesting they are not ectopic lumens. A white line delineates the two intestinal cells shown. L: lumen. Scale bars: 2µm.

The depletion of V-ATPases has previously been suggested to generate ectopic lumens in *C. elegans* intestines (*39*). It cannot be excluded that ectopic lumens were missed on multiple TEM sections of *vha-6(RNAi) rrf-3* intestines. However, this discrepancy with Bidaud-Meynard et al. (*39*) could also be explained by the organelle-specific assembly of different V-ATPase subunit isoforms into different pumps within the same intestinal cell, as, for instance, demonstrated in yeast (*36*). Note that, among a total of twelve V-ATPase subunits/subunit isoforms identified in our screens as required for apical domain biogenesis, VHA-1, -2 and -4 act as enhancers of GSLs' polarity function and are required for apical domain positioning (membrane polarity), whereas VHA-6 acts as a GSL suppressor and is not required for apical domain positioning (Fig. 3C).

(B) Confocal projections of ERM-1::GFP+ vacuolar aggregates (two aggregates are boxed) in *vha-6(RNAi)* larval intestinal cells (set of three cells surrounding the lumen are shown). RNAi was performed in an RNAi-sensitive background (*rrf-3*).Yellow arrows indicate EXC arm lumens. Scale bar: 5µm.

(C) Basolateral-membrane/cytoplasm SLCF-1::GFP fluorescence intensity ratio in expanding larval intestines (compare to Fig. 8B). Data are shown as mean ± SEM, n=3. One-tailed Student's *t*-test was used to calculate *P* values. ***P< 0.001.

(D) hTAC::GFP basolateral-membrane/cytoplasm fluorescence intensity ratio (left) and hTAC::GFP presence/absence on expanding larval intestinal membranes (right; compare to Fig. 8C). Data are shown as mean ± SEM, n=3. N≥7 (left), N≥30 (right). One-tailed Student's *t*-test was used to calculate *P* values. ***P< 0.001.

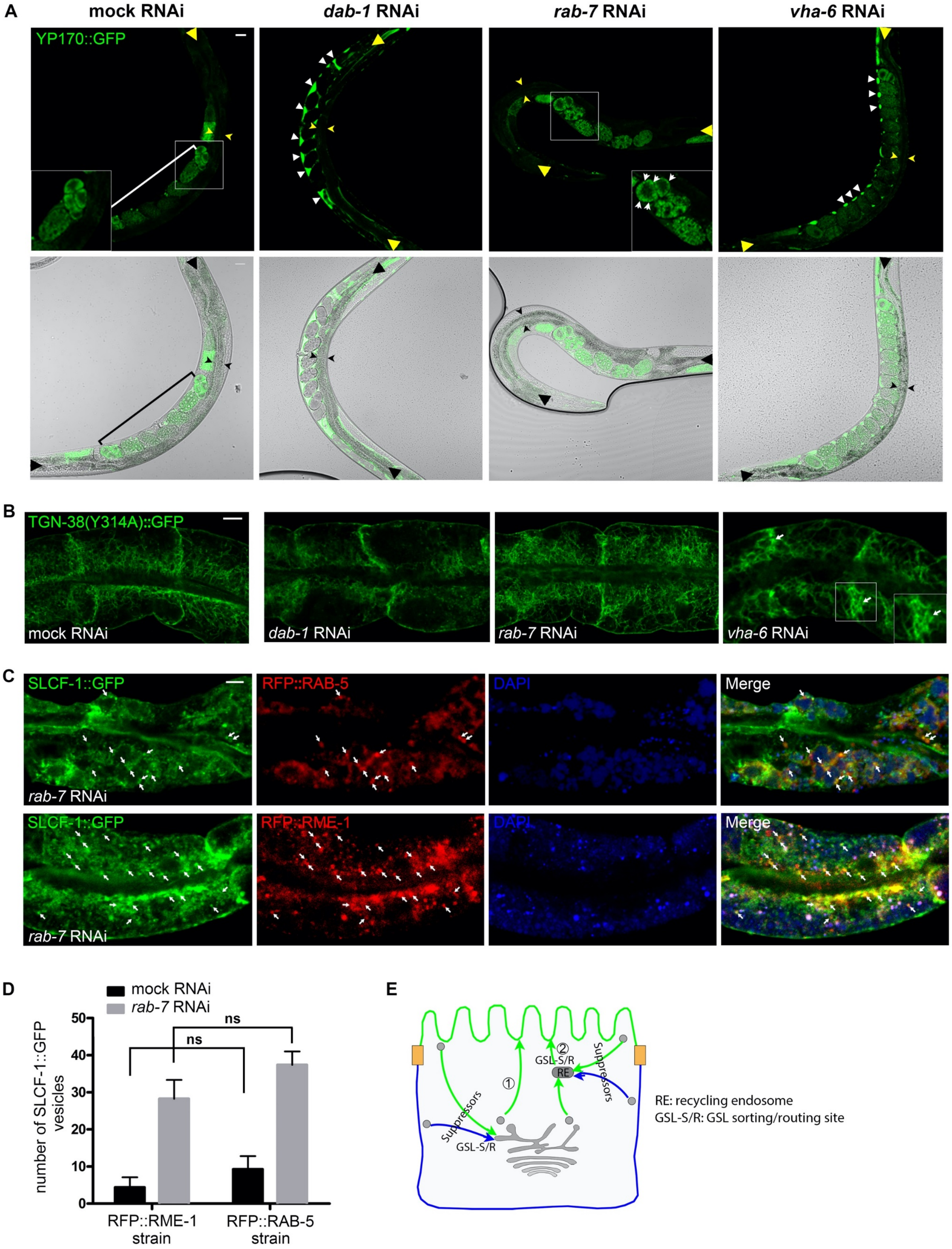
A
mock RNAi
dab-1 RNAi
rab-7 RNAi
vha-6 RNAi
YP170::GFP
B
TGN-38(Y314A)::GFP
mock RNAi
dab-1 RNAi
rab-7 RNAi
vha-6 RNAi
C
SLCF-1::GFP
RFP::RAB-5
DAPI
Merge
rab-7 RNAi
SLCF-1::GFP
RFP::RME-1
DAPI
Merge
rab-7 RNAi
D
mock RNAi
rab-7 RNAi
number of SLCF-1::GFP vesicles
50
40
30
20
10
0
ns
ns
RFP::RME-1 strain
RFP::RAB-5 strain
E
Suppressors
GSL-S/R
①
②
GSL-S/R
RE
Suppressors
RE: recycling endosome
GSL-S/R: GSL sorting/routing site

Fig. S8. DAB-1, RAB-7 and VHA-6 deliver polarized membrane components on recycling, not secretory, routes during net membrane expansion in wild-type (related to Fig. 9).

(A) *dab-1, rab-7 and vha-6* RNAi fail to disrupt Yolk secretion in the intestines of larvae and young adults. YP170/Yolk::GFP is secreted from *C. elegans* intestines into the body cavity (pseudocoelum), then endocytosed by oocytes (Materials and Methods). Disrupting endocytosis and secretion cause YP170::GFP accumulation in the body cavity and intestine, respectively (*105*). Note: (1) absence of intestinal YP170::GFP accumulation in all suppressor knockdowns, but pseudocoelomic YP170::GFP accumulation in *dab-1(RNAi)* animals (white arrowheads) with coincident lack of Yolk uptake into oocytes/embryos (previously described (*53*)); (2) YP170::GFP distribution defects in *rab-7(RNAi)* oocytes/embryos (see insets, white arrowheads; also previously described (*112*); and (3) mild YP170::GFP accumulation in the body cavity of *vha-6(RNAi)* animals (white arrowheads) with reduced uptake in oocytes/embryos. Mild RNAi conditions allowed animals to grow to maturity for which this assay was established. YP170::GFP accumulation was also absent in the intestines of L2-L3-stage larvae treated with stronger RNAi conditions (Materials and Methods). Top, confocal images, bottom, corresponding confocal/Nomarski overlays. Insets show higher magnification images of embryos (uterus with embryos is bracketed in first column). Yellow arrowheads (top) and corresponding black arrowheads (bottom) bracket widths (small arrowheads) and lengths (large arrowheads) of intestines.

(B) The recycling defective mutant TGN-38(Y314A) reaches the plasma membrane in expanding *dab-1-, rab-7-* and *vha-6(RNAi)* larval intestinal cells. Note lateral membrane broadening into a tubular network in the *vha-6(RNAi)* intestine (inset, arrows).

(C) Co-localization of SLCF-1 with RAB-5 and RME-1 in *rab-7(RNAi)* larval intestinal cells. Corresponding single-color images to Fig. 9, B and C. Arrows point to GFP+/RFP+/DAPI- vesicles, identifying double labeled vesicles (as opposed to auto-fluorescent triple positive LROs). See Fig. 9, B and C for legend.

Confocal images are shown throughout, full young adult intestines in (A), two pairs of cells surrounding the lumen of larval intestines in (B, C). Scale bars: 5µm.

(D) The RFP::RME-1 and RFP::RAB-5 transgenic backgrounds do not affect SLCF-1::GFP displacement to cytoplasmic vesicles by *rab-7* RNAi in larval intestinal cells. Also note the significant increase of cytoplasmic SLCF-1+ vesicles in *rab-7(RNAi)* intestinal cells that demonstrates the efficacy of *rab-7* RNAi in these colocalization experiments and quantifies the vesicular SLCF-1 displacement during membrane expansion for this specific suppressor. Data are shown as mean $\pm$ SEM, n=3/N=8. Two-way ANOVA was performed to determine statistical significance. ns, not significant.

(E) The itinerary of a post-Golgi GSL-directed secretory apical vesicle trajectory and the location of GSL sorting/routing sites can be deduced from the route on which the suppressors might function (compare with Fig. 7). Possibilities include: (1) a direct route to the apical membrane (route 1), with corresponding GSL sorting/routing site at Golgi or pre-Golgi endomembranes (suppressors function on pre-Golgi-/Golgi-directed retrograde recycling routes);

(2) an indirect route to the apical membrane, with GSL sorting/routing site at the recycling endosome or at other, e.g., apical, sorting endosomes (route 2; the suppressors function on endocytic-recycling routes). A transcytotic route (Fig. 7A, route 4) is unlikely, as it would implicate a suppressor function on secretory routes and GSL sorting/routing site at, or beyond, the basolateral membrane.

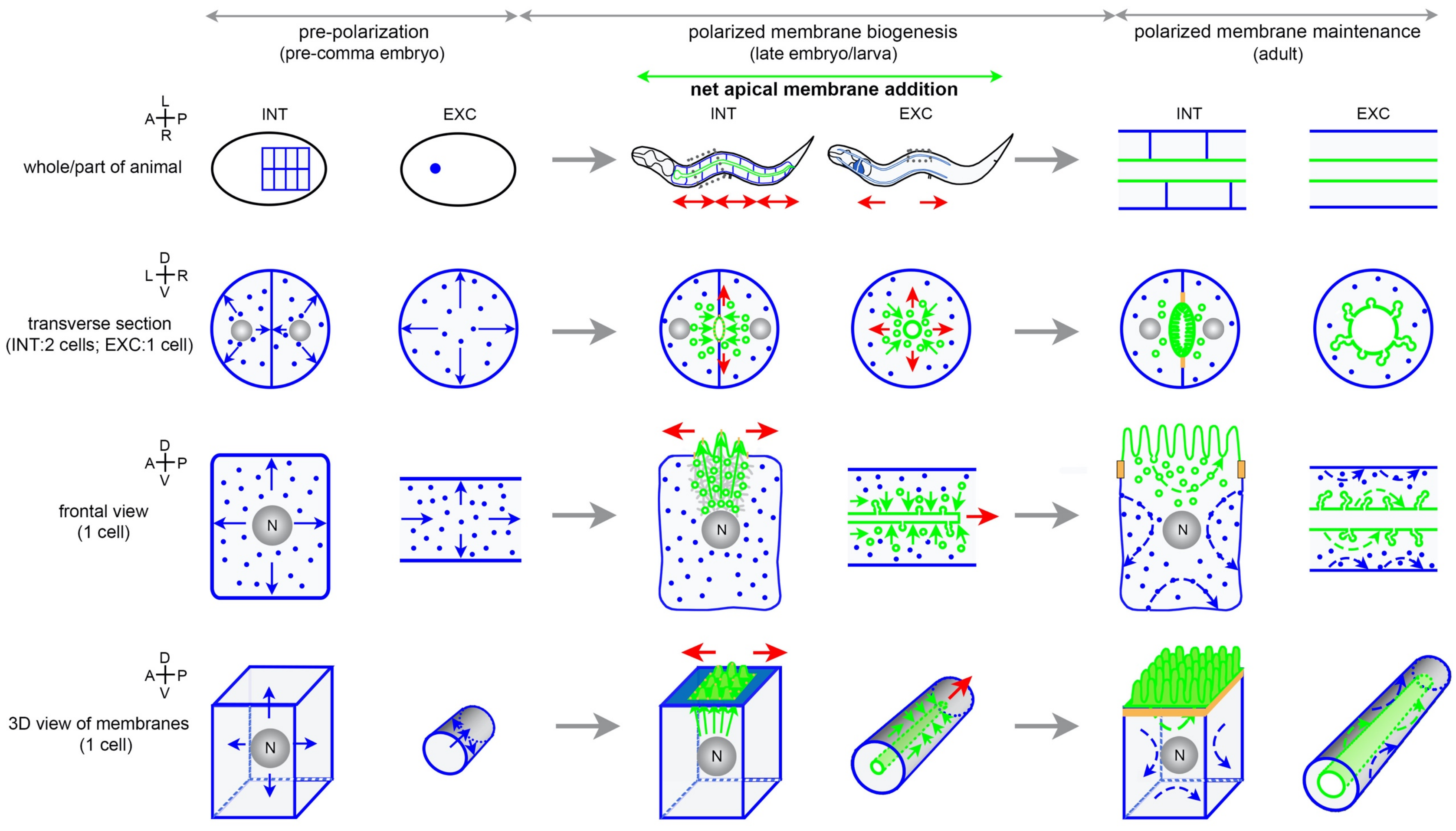

pre-polarization
(pre-comma embryo)
polarized membrane biogenesis
(late embryo/larva)
polarized membrane maintenance
(adult)
net apical membrane addition
INT
EXC
INT
EXC
INT
EXC
whole/part of animal
transverse section
(INT:2 cells; EXC:1 cell)
frontal view
(1 cell)
3D view of membranes
(1 cell)
N

Fig. S9. An apicobasal polarity model based on net apical membrane addition. 3D tissue context (related to Fig. 10).

The asymmetric addition of apical membrane extends the inTRAcellular lumen in the single-cell excretory canal and inserts an inTERcellular lumen between pairs of cells in the single-layered intestine, thereby defining the position of apicobasal membrane domains.

Each of the three double columns show intestinal (INT; left) and excretory canal (EXC; right) apical membrane biogenesis side by side. The insertion of the nascent apical membrane (middle columns; red arrows) into the non-polarized plasma membrane (left double columns) generates a plasma membrane with apical and basolateral domains (right double columns). It extends the plasma membrane of intestinal cells with an apical domain and the excretory canal cell membrane with an endomembrane of apical character.

Whole/parts of animals/tissues are shown in the upper row (boxed areas in the middle column are magnified in the right column) and different views of single cells are shown beneath. Apical and basolateral (or non-polarized) endo- and plasma membranes are shown in green and blue, respectively (see Fig. 1A and fig. S1 for INT and EXC tubulogenesis). Trafficking routes/directions are simplified for clarity: only secretory routes (solid arrows) are shown in the left and middle double columns; only apical secretory routes in the middle double columns; only recycling routes (dashed arrows) in the right double columns. Indirect trafficking routes (via endosomes) and interfacing (e.g., transcytotic) routes to the nascent apical domain are omitted (compare Fig. 7A). The transient dynamic cytoskeleton (see our accompanying article (*51*)), proposed to move vesicles towards the nascent apical domain in the INT, is indicated in the 3rd row, middle column (grey); junctions (orange) are shown in the 3rd and 4th rows. N: nucleus. A/anterior, P/posterior; D/dorsal, V/ventral; R/right, L/left.

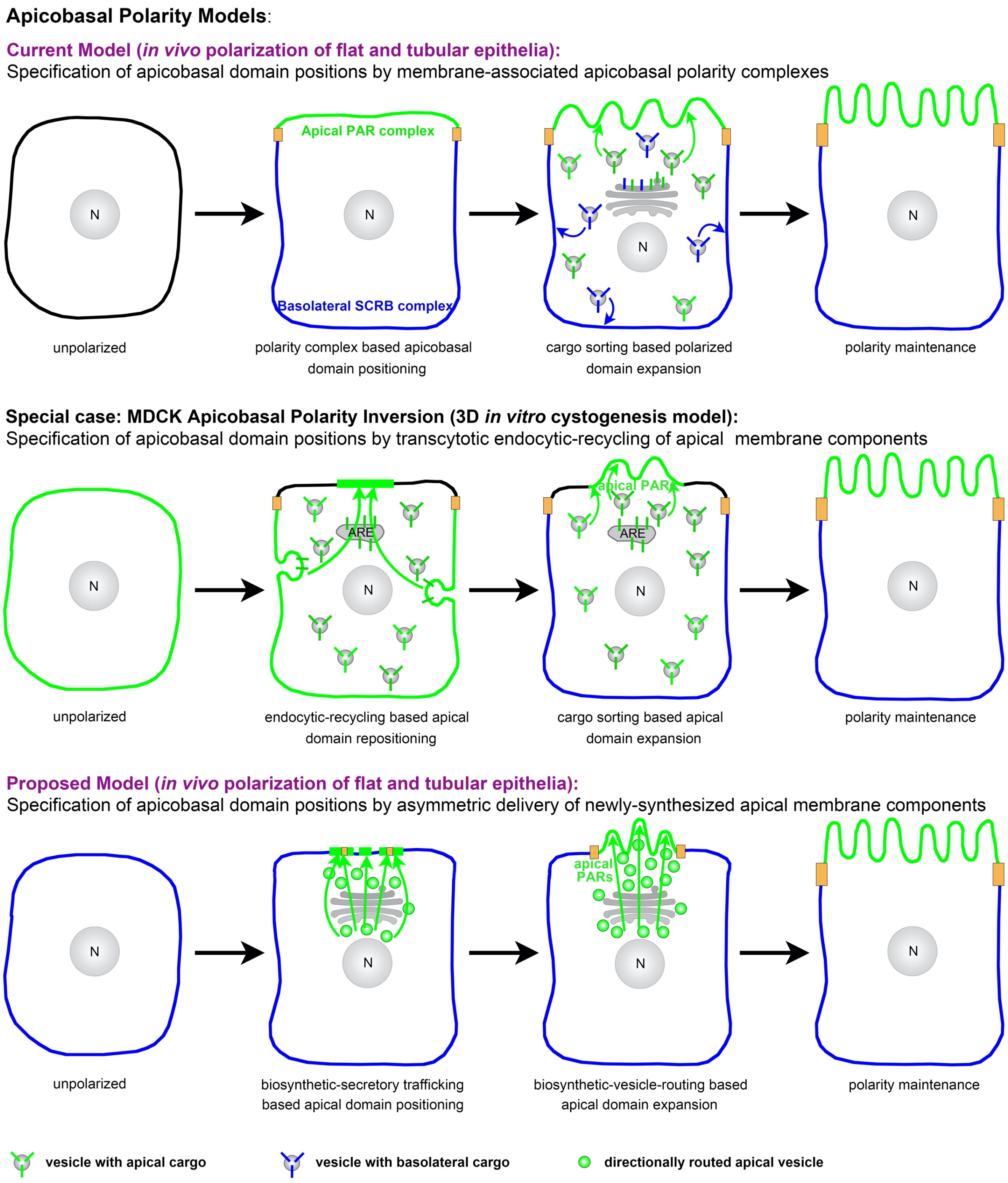
Apicobasal Polarity Models:
Current Model (*in vivo* polarization of flat and tubular epithelia):
Specification of apicobasal domain positions by membrane-associated apicobasal polarity complexes
N
Apical PAR complex
Basolateral SCRB complex
unpolarized
polarity complex based apicobasal domain positioning
cargo sorting based polarized domain expansion
polarity maintenance
Special case: MDCK Apicobasal Polarity Inversion (3D *in vitro* cystogenesis model):
Specification of apicobasal domain positions by transcytotic endocytic-recycling of apical membrane components
ARE
apical PARs
unpolarized
endocytic-recycling based apical domain repositioning
cargo sorting based apical domain expansion
polarity maintenance
Proposed Model (*in vivo* polarization of flat and tubular epithelia):
Specification of apicobasal domain positions by asymmetric delivery of newly-synthesized apical membrane components
apical PARs
unpolarized
biosynthetic-secretory trafficking based apical domain positioning
biosynthetic-vesicle-routing based apical domain expansion
polarity maintenance
vesicle with apical cargo
vesicle with basolateral cargo
directionally routed apical vesicle

Fig. S10. Comparison of epithelial polarity models (related to Fig. 10). Apicobasal membrane domain biogenesis by: domain composition change (current model); domain composition change by transcytosis (MDCK cystogenesis); domain insertion by *de novo* polarized membrane biogenesis (proposed model).

Schematics of polarizing epithelial cells, as shown in Fig. 10, with the addition of the *in vitro* Madin-Darby-Canine-Kidney/MDCK cyst model (row 2; see text; (*20*)). See Fig. 10 legend for the description of cartoons and details of rows 1 and 3 (current and proposed polarity models).

The special case of MDCK cyst polarity inversion (row 2), initiated by a change from 2D to 3D culture conditions, mirrors *C. elegans* intestinal polarity conversion. Note that during MDCK cystogenesis the apical membrane transcytoses from the future basolateral to the new apical location via endocytic-recycling. The apical recycling marker RAB-11/Rab11 is required for apical domain positioning during both MDCK cystogenesis and *C. elegans* intestinal tubulogenesis, suggesting that these different trafficking routes might converge in the vicinity of the nascent apical domain (e.g., at the ARE; see Fig. 10 inset).

Table S1. Vesicular trafficking genes for apical membrane biogenesis/lumen morphogenesis, identified in several *C. elegans* multi- and unicellular tubulogenesis screens (tier-1 genes; related to Fig. 2).

Table S2. Classification of the identified tier-1 trafficking genes by trafficking route/function. Tier-1 trafficking genes previously identified in genetic screens on apical cargo delivery and secretion and tier-1 trafficking genes previously implicated in apical domain positioning (related to Fig. 4).

Table S3. The time course of polarity conversion in double *let-767(-/-)* mutant/suppressor knockdowns (related to Fig. 7, B and C).

Table S4. Reagent and Strain list.

Movie S1. 3D view of apical junction integrity in *copz-1(RNAi)* larval intestine (related to Fig. 4 and fig. S5).
